# Synthesis and bioactivities evaluation of oleanolic acid oxime ester derivatives as *α*-glucosidase and *α*-amylase inhibitors

**DOI:** 10.1080/14756366.2021.2018682

**Published:** 2022-01-10

**Authors:** Xu-Yang Deng, Jun-Jie Ke, Ying-Ying Zheng, Dong-Li Li, Kun Zhang, Xi Zheng, Jing-Ying Wu, Zhuang Xiong, Pan-Pan Wu, Xue-Tao Xu

**Affiliations:** a School of Biotechnology and Health Sciences, Wuyi University, Jiangmen, P.R. China; b Department of Chemical Biology, Ernest Mario School of Pharmacy, Rutgers, The State University of New Jersey, Piscataway, NJ, USA

**Keywords:** Oleanolic acid, structural modification, *α*-glucosidase, *α*-amylase, enzyme inhibition

## Abstract

Different oleanolic acid (OA) oxime ester derivatives (**3a**-**3t**) were designed and synthesised to develop inhibitors against *α*-glucosidase and *α*-amylase. All the synthesised OA derivatives were evaluated against *α*-glucosidase and *α*-amylase *in vitro.* Among them, compound **3a** showed the highest *α*-glucosidase inhibition with an IC_50_ of 0.35 µM, which was ∼1900 times stronger than that of acarbose, meanwhile compound **3f** exhibited the highest *α*-amylase inhibitory with an IC_50_ of 3.80 µM that was ∼26 times higher than that of acarbose. The inhibition kinetic studies showed that the inhibitory mechanism of compounds **3a** and **3f** were reversible and mixed types towards *α*-glucosidase and *α*-amylase, respectively. Molecular docking studies analysed the interaction between compound and two enzymes, respectively. Furthermore, cytotoxicity evaluation assay demonstrated a high level of safety profile of compounds **3a** and **3f** against 3T3-L1 and HepG2 cells.

**Highlights**
1. Oleanolic acid oxime ester derivatives (**3a–3t**) were synthesised and screened against α-glucosidase and α-amylase.
2. Compound **3a** showed the highest α-glucosidase inhibitory with IC50 of 0.35 µM.
3. Compound **3f** presented the highest α-amylase inhibitory with IC50 of 3.80 µM.
4. Kinetic studies and *in silico* studies analysed the binding between compounds and α-glucosidase or α-amylase.

## Graphical abstract

**Figure:**
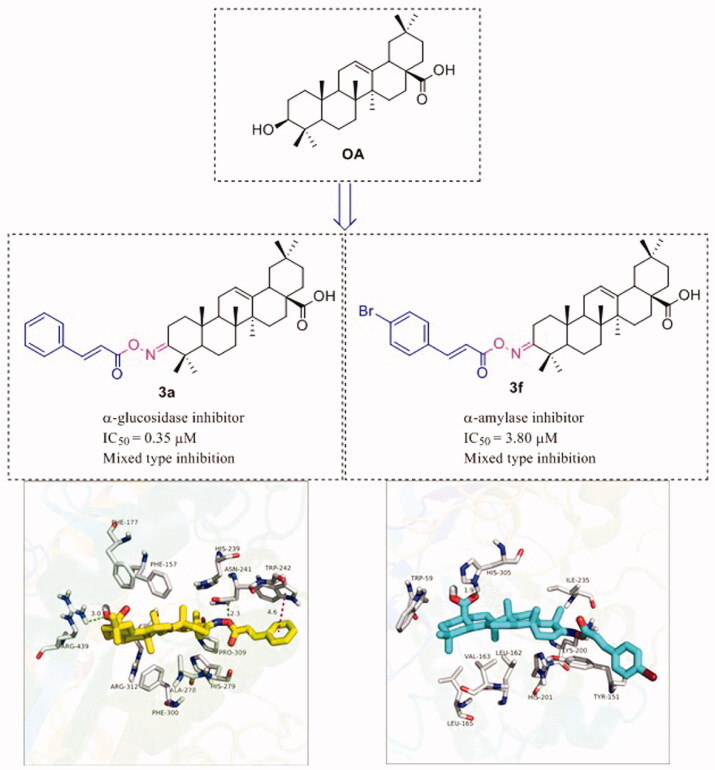

## 1. Introduction

Diabetes mellitus (DM), characterised by chronic hyperglycaemia, has become a serious globalised problem threatening human health^1^. It is estimated that more than 460 million people have been suffering from DM with the number of people with DM reaching more than 570 million by 2030^2^. In the light of the causative mechanism, diabetes can be categorised into four classes as follows: type-1 diabetes (T1D), type-2 diabetes (T2D), gestational diabetes (GD) and specific types of diabetes^3^. As the most common type of diabetes, T2D, resulting from insulin resistance and pancreatic *β*-cell dysfunction, takes up 90% of all diabetes cases^4^. In addition, the excessive intake in carbohydrates can accelerate the outbreak of T2D^5^. Carbohydrates can decompose into glucose by the synergistic action of several intestinal enzymes, including *α*-glucosidase and α-amylase, leading to the elevated blood glucose level in the gastrointestinal tract^6^^,^^7^. When carbohydrates are consumed, α-glucosidase hydrolyses carbohydrates to absorbable glucose and α-amylase catalyses the hydrolysis of starch to maltose and glucose^8^. Therefore, the management of T2D can be achieved by reducing postprandial hyperglycaemia through the inhibition of *α*-glucosidase and α-amylase hydrolysis^9^. Acarbose was the effective clinical hypoglycaemic drugs targeting *α*-glucosidase and α-amylase. But it still had adverse gastrointestinal side effects, inspiring us to develop new inhibitors.

Oleanolic acid (OA), one of the naturally occurring pentacyclic triterpenoid compounds, was widely found in plant-derived food and medicinal herbs^10–14^. OA and its derivatives possess different biological activities such as anti-inflammatory, anti-hyperglycaemic, anti-hyperlipidaemic, anti-oxidant and hepatoprotective^1^. The research found that the introduction of oxime-ester moiety into OA can improve its anti-tumour activity^15^, anti-inflammatory effect^16^, the inhibition of HIV-1 protease dimerisation and the inhibitory activity of glycogen phosphorylase compared with the parent compound^17^^,^^18^ and other activities, which was presumably associated with the enhancement of the hydrophilic performance^19^. Furthermore, our group has been dedicated to the structural modification and derivation of OA skeleton at the C2, C3, C12, C13, or C28 position, simultaneously the inhibition of α-glucosidase and its mechanism have been explored in depth^20^^,^^21^.

Based on the findings above, the conjugation of OA with substituted cinnamic acid and benzoic acid through oxime linkage have been developed as our ongoing work being aimed to obtain the leading compounds in inhibiting glycosidase. In this study, oleanolic acid oxime ester derivatives **3a**-**3t** were synthesised by acylation of oleanolic acid oxime, followed by the inhibitory activity evaluation against α-amylase and α-glucosidase.

## 2. Results and discussion

### 2.1. Chemistry

**Scheme 1.**
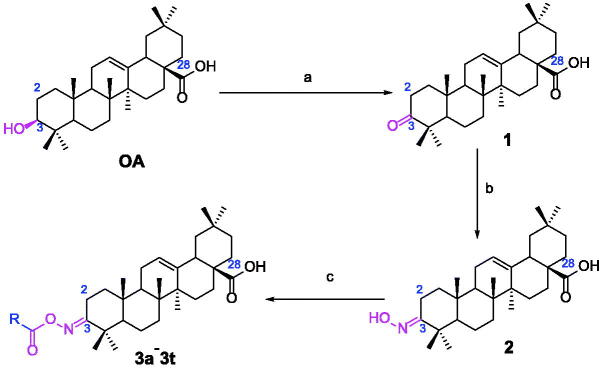
Synthesis of oleanolic acid oxime ester derivatives (**3a**–**3t**). Reagents and conditions: (a) Jones reagent, acetone, 0 °C; (b) NH_2_OH·HCl (1.0 eq.), EtOH, NaOAc (2.0 eq.), rt, 1 h; (c) cinnamoyl chlorides and benzoyl chlorides (2.0 eq.), pyridine, DCM, 0 °C ∼ rt, overnight.

In order to obtain oleanolic acid oxime ester derivatives **3a**–**3t**, the C3 position of OA scaffold was selected as modification site for the derivation according to the synthetic route concretely depicted in Scheme 1. At first, OA was oxidised to 3-Oxo-olean-12-en-28-oic acid by the freshly prepared Jones reagent, further reacting with hydroxylamine hydrochloride to produce oleanolic acid oxime **2**. Then target compound **3a–3t** were prepared through acylation of the oleanolic acid oxime intermediate **2** with a diversity of cinnamoyl chlorides and benzoyl chlorides. All synthetic derivatives were purified *via* column chromatography and their structures were identified by ^1^H NMR, ^13 ^C NMR and HRMS.

### 2.2. Inhibitory effect of OA derivatives against α-glucosidase

#### 2.2.1. α-Glucosidase inhibition assay and SAR analysis

The *α*-glucosidase inhibitory activity of OA derivatives (**3a**–**3t**) was evaluated with acarbose as the standard inhibitor. As shown in Table 1, all the synthesised OA derivatives (**3a**–**3t**) had potent inhibitory activity against *α*-glucosidase with the IC_50_ values ranging from 0.35 to 3.43 µM. It was worth noting that all OA derivatives (**3a**–**3t**) showed stronger inhibitory activity than OA (IC_50_ = 4.09 µM) and acarbose (IC_50_ = 665.56 µM). Compound **3a** exhibited the strongest inhibitory activity (IC_50_ = 0.35 µM), ∼11 times stronger than that of OA and ∼1900 times stronger than that of acarbose. The results indicated that the introduction of cinnamoyloxyimino moiety at OA-C3 position could enhance the anti-*α*-glucosidase activity of OA derivatives.

**Table 1.** Inhibition of all the synthesised OA derivatives against *α*-glucosidase and *α*-amylase. 
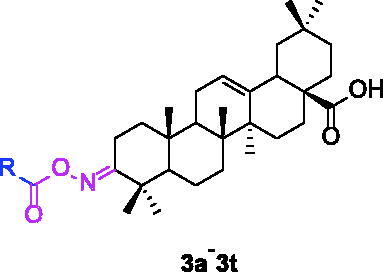

Compounds	R	*α*-Glucosidase inhibition (IC_50_:μM)	*α*-Amylase inhibition (IC_50_:μM)
**3a**	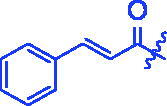	0.35 ± 0.06^a^	7.86 ± 1.24^a^
**3b**	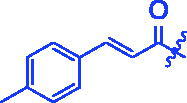	0.68 ± 0.03^a^	15.26 ± 1.41^a^
**3c**	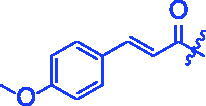	0.89 ± 0.07^a^	25.37 ± 1.17^a^
**3d**	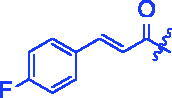	1.45 ± 0.12^a^	17.47 ± 1.52^a^
**3e**	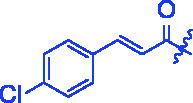	0.95 ± 0.06^a^	5.64 ± 1.29^a^
**3f**	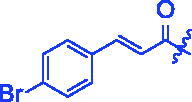	1.28 ± 0.03^a^	3.80 ± 0.65^a^
**3g**	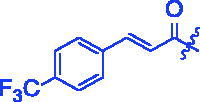	3.13 ± 0.13^a^	12.57 ± 2.37^a^
**3h**	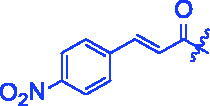	1.36 ± 0.03^a^	18.29 ± 1.92^a^
**3i**	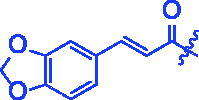	0.71 ± 0.02^a^	17.87 ± 1.21^a^
**3j**	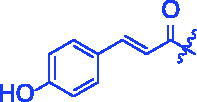	0.82 ± 0.09^a^	70.81 ± 2.31^a^
**3k**	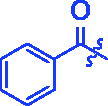	0.67 ± 0.04^a^	25.57 ± 1.66^a^
**3l**	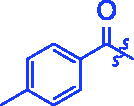	0.99 ± 0.05^a^	55.30 ± 2.39^a^
**3m**	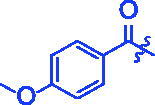	1.10 ± 0.10^a^	35.47 ± 2.41^a^
**3n**	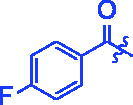	1.96 ± 0.25^a^	70.21 ± 2.53^a^
**3o**	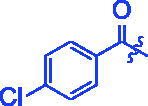	1.74 ± 0.14^a^	26.32 ± 1.61^a^
**3p**	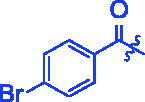	1.81 ± 0.05^a^	15.93 ± 1.75^a^
**3q**	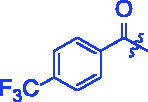	3.43 ± 0.17 ^a^	33.61 ± 1.93^a^
**3r**	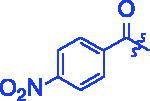	1.41 ± 0.12^a^	70.72 ± 1.25^a^
**3s**	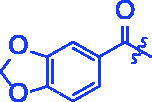	1.37 ± 0.06^a^	18.68 ± 1.29^a^
**3t**	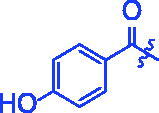	0.91 ± 0.04^a^	152.36 ± 4.24^a^
**OA**		4.09 ± 0.11^b^	94.10 ± 1.72^b^
**Acarbose**		665.56 ± 13.92^c^	100.01 ± 2.73^c^

Values are the mean ± SD. Mean values in the same column having different letters are significantly different (*p* < 0.05).

For better understanding the structure activity relationships (SAR), OA derivatives were categorised into two groups, including **A** group (OA derivatives **3a**–**3j**) and **B** group (OA derivatives **3k**–**3t**). In **A** group, derivative **3a** was selected as template compound, it could be seen that the introduction of functional groups (methyl, methoxy, fluorine, chlorine, bromine, trifluoromethyl, nitro, methylenedioxy and hydroxyl) at C4 position belonging to the cinnamoyl group, weakened inhibitory activity compared with the unsubstituted compounds **3a**. In **B** group, derivative **3k** was selected as template compound, and found the similar results with **A** group that is introduction of functional groups weakened inhibitory activity. Besides, OA derivatives in **A** group had a higher inhibitory activity than OA derivatives with the same substituent in **B** group, respectively. Thence, the incorporation of cinnamoyloxyimino moiety into the OA parent structure could improve the inhibitory activity of OA derivatives better than that of benzoyl groups.

#### 2.2.2. Inhibitory kinetic analysis

**Figure 1.**
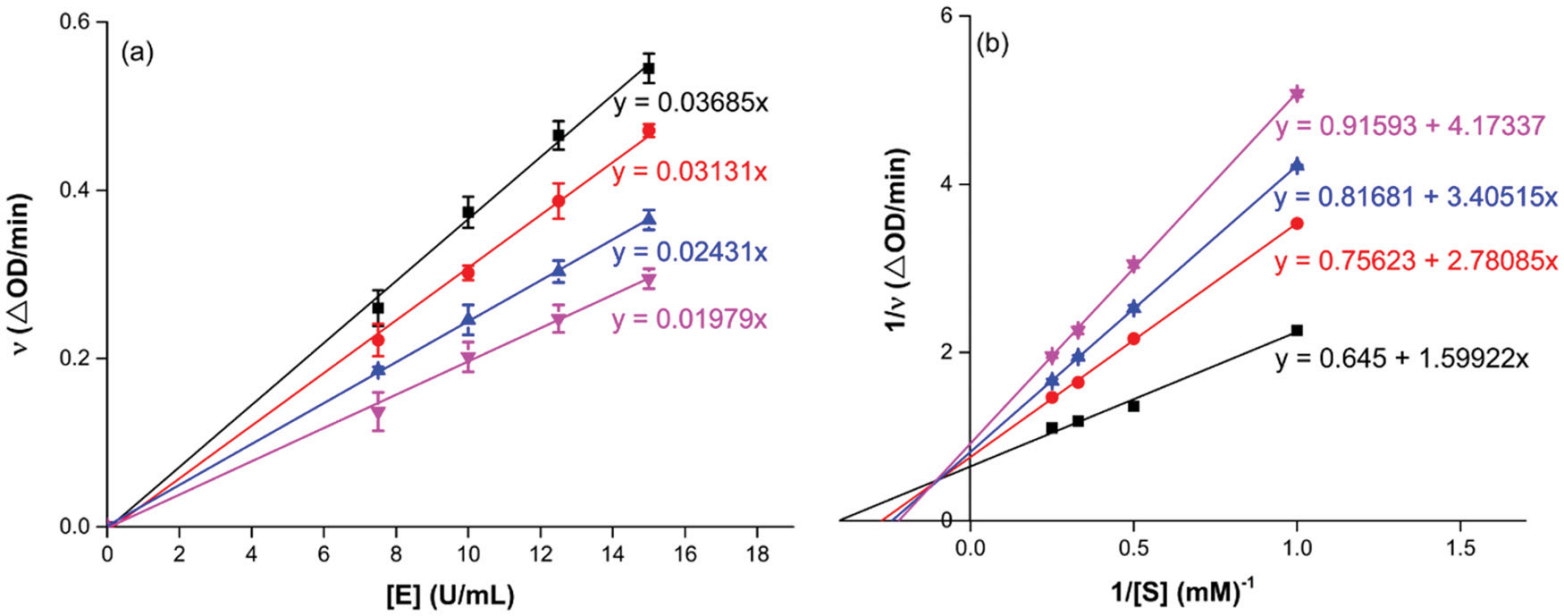
Inhibition kinetics of **3a** on α-glucosidase. (a) The plots of enzymatic reaction rate *vs α*-glucosidase concentration with or without the presence of **3a**; (b) Lineweaver–Burk plots of enzymatic reaction rate versus substrate concentration with or without the presence of **3a** (**3a** concentration: black line, 0 μM; red line, 0.25 μM; blue line, 0.45 μM; pink line, 0.5 μM).

To probe into the inhibition mechanism of OA derivatives (**3a**–**3t**) against *α*-glucosidase, compound **3a** with the strongest inhibitory activity was selected as the representative compound for the enzyme inhibitory kinetic analysis. The plots of *V* vs. [E] with or without the presence of compound **3a** (Figure 1(a)) generated straight lines which all passed through the origin, suggesting that the inhibition of compound **3a** was reversible. The inhibition kinetic behaviours were investigated using the Lineweaver–Burk plots. The plots of 1/*V* vs. 1/[*S*] with or without the presence of compound **3a** gave straight lines intersecting in the second quadrant, indicating compound **3a** was mixed-type inhibitor (Figure 1(b)). These results revealed that compound **3a** inhibited *α*-glucosidase by binding with free enzyme, as well as enzyme–substrate complex.

#### 2.2.3. Docking simulation for α-glucosidase

**Figure 2.**
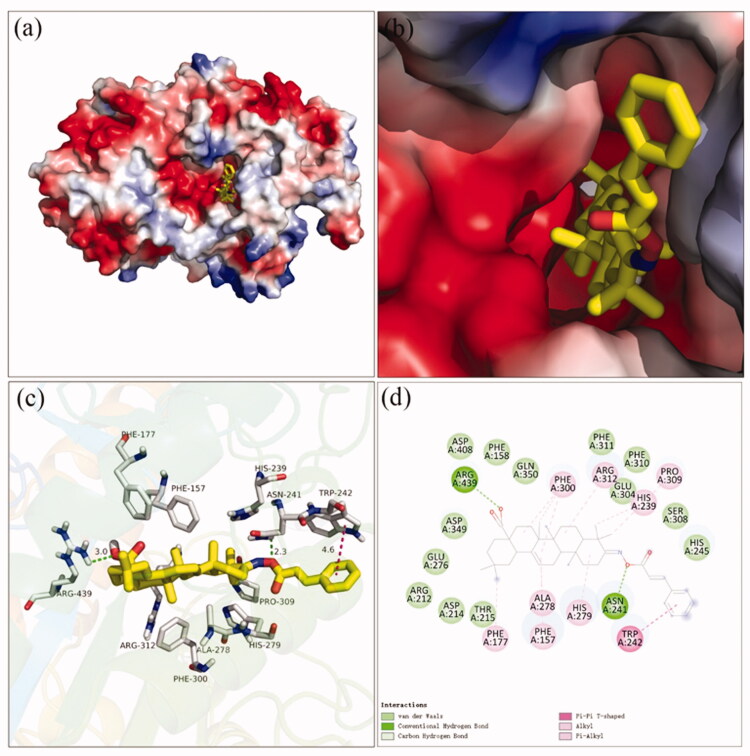
The molecular docking of analogue **3a** with *α*-glucosidase (homology mode): (a) **3a** in the electrostatics active pocket; (b) **3a** in the active pocket; (c) 3D view of **3a** with *α*-glucosidase; (d) 2D view of **3a** with *α*-glucosidase.

Docking simulation was performed to clarify the interactions between compound and active pocket of *α*-glucosidase. The most potent compound **3a** was docked in the active site of *α*-glucosidase. As shown in Figure 2(a,b), compound **3a** was well nested into the active site, and OA part located inside of the active site. The detailed interaction results between compounds **3a** with active site residues were shown in Figure 2(c,d) using 3D and 2D views, respectively. The carboxylic oxygen at OA part formed a hydrogen bond with Arg439 (3.0 Å), esteryl oxygen at cinnamic acid part made a hydrogen bond with Asn241 (2.3 Å), cinnamic acid benzene ring had a π-π interaction with Trp242 (4.6 Å), and hydrophobic interaction with Phe157, Phe177, His239, Ala278, His279, Phe300, Pro309, and Arg312. The binding affinity of compound **3a** regarding *α*-glucosidase was −3.7 kcal/mol. Furthermore, the physicochemical properties of most potent compound **3a** was listed in Table 2.

**Table 2.** The physicochemical properties of compounds **3a** and **3f**.

	Molecular formula	Molecular weight	Rotatable bonds	H-bond acceptoer atoms	H-bond donnor atoms	Polar surface area (Å^2^)	LogP_o/w_	Water solubility
**3a**	C_39_H_53_NO_4_	599.84	5	5	1	75.96	7.82	Poorly soluble
**3f**	C_39_H_52_BrNO_4_	678.74	5	5	1	75.96	8.49	Insoluble

### 2.3. Inhibitory effect of OA derivatives against α-amylase

#### 2.3.1 α-Amylase inhibition assay and SAR analysis

The inhibitory activities of OA derivatives against *α*-amylase were also tested (Table 1). All candidate compounds had potent inhibitory activities towards *α*-amylase with IC_50_ values ranging from 3.80 to 152.36 µM. OA derivatives showed higher inhibitory activities than OA (IC_50_ = 94.10 µM) and acarbose (IC_50_ = 100.01 µM) except compound **3t**. Among them, compound **3f** had the strongest inhibitory activity, ∼25 times better than OA and ∼26 times better than acarbose. Hence, it was an effective way to increase the inhibitory activities of OA against *α*-amylase by embedding the cinnamoyloxyimino moiety into the OA motif.

For SAR analysis, it could be found that the introduction of chloro or bromo group at C4 position of benzene ring belonging to the cinnamoyl group could improve the inhibitory activity against *α*-amylase of OA derivatives (compounds **3e** and **3f**). But when substituents such as methyl, methoxyl, fluoro, trifluoromethyl, nitro, methylenedioxy or hydroxyl installed into C4 position of benzene ring, the inhibitory activity against *α*-amylase of OA derivatives had decreased. In addition, that the icinnamoyloxyimino substructure was merged with the OA parent structure (compounds **3a**–**3j**) had positive effect on the improvement of anti-*α*-amylase activity.

#### 2.3.2. Inhibitory kinetic analysis

**Figure 3.**
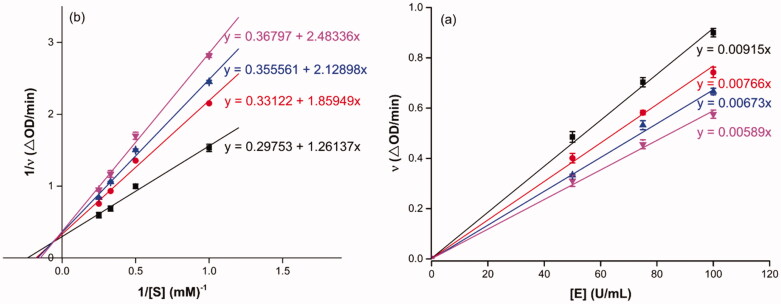
Inhibition kinetics of **3f** against α-glucosidase. (a) The plots of enzymatic reaction rate *vs α*-glucosidase concentration with or without the presence of **3f**; (b) Lineweaver–Burk plots of enzymatic reaction rate *vs* substrate concentration with or without the presence of **3f** (**3f** concentration: black line, 0 μM; red line, 5 μM; blue line, 7.5 μM; pink line, 15.0 μM).

Compound **3f** with the strongest inhibitory activity against *α*-amylase was selected as the representative compound for the enzyme inhibitory kinetic analysis. As shown in Figure 3, the plots of *V* vs. *α*-amylase concentration with or without the presence of compound **3f** all passed through the origin (Figure 3(a)) and the Lineweaver–Burk plots of 1/*V* vs. 1/[*S*] with or without the presence of compound **3f** intersected in the second quadrant (Figure 3(b)). The results indicated compound **3f** was reversible and mixed-type inhibitor.

#### 2.3.3. Docking simulation for α-amylase

**Figure 4.**
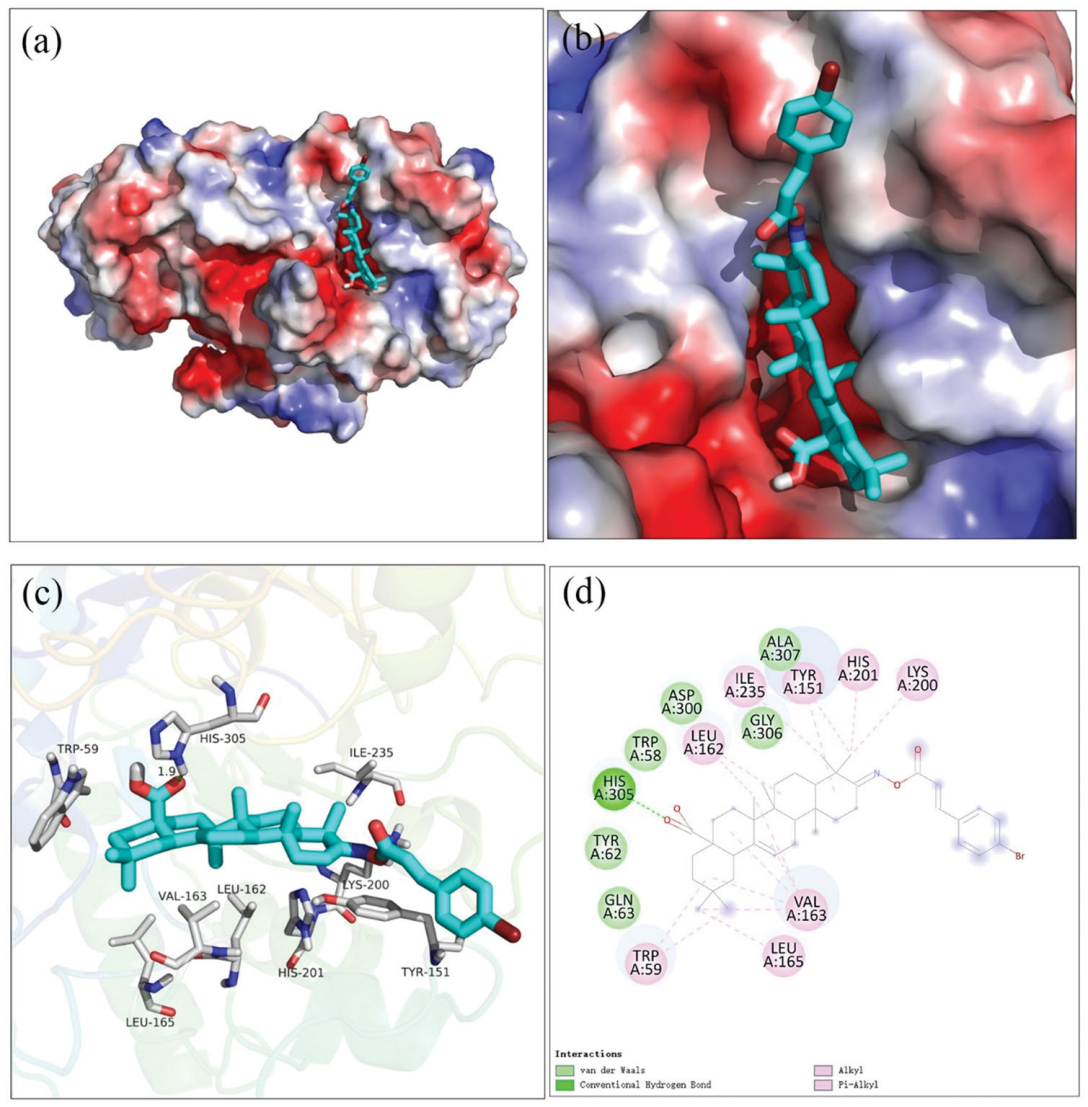
The molecular docking of analogue **3f** and *α*-amylase (3BAJ): (a) **3f** in the electrostatics active pocket; (b) **3f** in the active pocket; (c) 3D view of **3f** and *α*-amylase; (d) 2D view of **3f** and *α*-amylase.

The docking simulation results between compound **3f** and *α*-amylase were shown in Figure 4. It was observed that OA part of compound **3f** located into the active site of α-amylase and cinnamic acid part located near the edge of the active site (Figure 4(a,b)). The carboxylic oxygen at OA part formed a hydrogen bond with His305 (1.9 Å), and hydrophobic interaction with Trp59, Leu162, Val163, Leu165, Tyr151, Lys200, His201, and Ile235. The binding affinity of compound **3f** regarding *α*-amylase was −2.1 kcal/mol. The physicochemical properties of **3f** was also shown in Table 2.

### 2.4. Cells cytotoxicity assay

**Figure 5.**
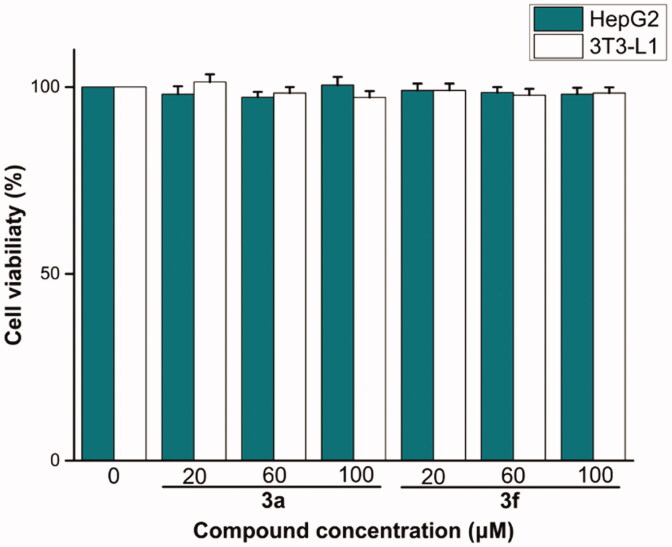
Cells cytotoxicity of compounds **3a** and **3f** against 3T3-L1 and HepG2 cells.

The preliminary *in vitro* cells cytotoxicity of most potent compounds **3a** and **3f** were evaluated against 3T3-L1 and HepG2 using MTT method. Compounds **3a** and **3f** had non-cytotoxic to 3T3–L1 and HepG2 cells under concentration of 100 µM (Figure 5), indicating that the compounds had a high level of safety profile.

## 3. Conclusion

In summary, OA oxime ester derivatives (**3a**–**3t**) were synthesised and evaluated for inhibitory activities against *α*-glucosidase and *α*-amylase. All the synthesised OA derivatives had potent inhibitory activities against *α*-glucosidase and *α*-amylase. The introduction of cinnamoyloxyimino moiety into the OA parent structure had better *α*-glucosidase and α-amylase inhibitory activities than that of benzoyloxyimino moiety. Compound **3a** had highest inhibitory activity against *α*-glucosidase with IC_50_ value of 0.35 µM, ∼1900 times stronger than acarbose, meanwhile compound **3f** had the highest *α*-amylase inhibitory activity with IC_50_ value of 3.80 µM, ∼26 times higher than acarbose. Furthermore, the inhibition kinetic studies and docking simulation indicated the binding between compounds with protein and the low toxicity against 3T3-L1 and HepG2 cells provided the foundation for further *in vivo* assay. Considering all these experimental data, OA oxime ester derivatives could be developed into the leading compound in the management of T2D.

## 4. Experimental

### 4.1. Materials and methods

*α*-Glucosidase from *Saccharomyces cerevisiae* (EC 3.2.1.20), *α*-amylase from hog pancreas (EC3.2.1.1), *p*-Nitrophenyl-*α*-D-galactopyranoside (*p*-NPG). Water-soluble starch was obtained from Shanghai Yuanye Biological Technology Co., Ltd. OA was purchased from Macklin Co., Ltd. Other chemical reagents were obtained from Titan (Shanghai) Technology Co., Ltd. 3T3-L1 cells and HepG2 cells were supplied by ATCC. 3–(4,5-Dimethythiazol-2-yl)-2,5-diphenyl-tetrazoliumbromide (MTT) were supported by Sigma-Aldrich. Dulbecco’s Modified Eagle’s Medium (DMEM), Foetal bovine serum (FBS), penicillin and streptomycin were obtained from Gibco.

^1^H NMR and ^13 ^C NMR spectra were recorded in CDCl3 on 500 MHz instruments. High-resolution mass spectral analysis (HRMS) data were measured on the Apex II by means of the ESI technique.

### 4.2. *Synthesis of* OA derivatives *3a*–*3t*

To a solution of oleanolic acid (1.0 mmol) in acetone (10 ml), freshly Jones reagent was added dropwise in an ice bath. The reaction mixture allowed to warm to room temperature and stirred for 4 h. Firstly, MeOH was added and then water to the reaction mixture. The organic solvent was removed under vacuum and the aqueous residue was extracted with EtOAc. The organic phase was washed with water and brine, dried by MgSO_4_ and evaporated under vacuum. Then, the crude product was purified by silica gel column chromatograph to obtain compound **1**.

Subsequently, to a solution of NH_2_OH**·**HCl (1.5 mmol) in ethanol (10 ml) was added NaOAc (2.0 mmol) and the mixture was stirred for 15 min at room temperature. Compound **1** was added to the reaction mixture and the mixture was stirred for 24 h at 50 °C. After ethanol was removed under vacuum, the residue was treated with water and filtrated to afford compound **2**.

Finally, freshly prepared substituted cinnamoyl chloride or benzoyl chloride (1.2 mmol) was added into the solution of compound **2** (1.0 mmol) and pyridine in DCM at 0 °C, and then, the mixture was stirred at room temperature until completion. The reaction was quenched with saturated NaHCO_3_ and extracted by EtOAc. The organic phase was washed with water and brine, dried by MgSO_4_ and removed under vacuum to obtain the residue followed by purification through silica gel column chromatograph to produce the corresponding oleanolic acid oxime ester derivatives **3a**–**3t**.

**8a** (C_39_H_53_NO_4_). White powder; Yield 68%. ^1^H NMR (500 MHz, Chloroform-*d*) *δ* 7.78 (d, *J* = 16.0 Hz, 1H), 7.55 (dt, *J* = 6.7, 3.9 Hz, 2H), 7.42 − 7.36 (*m*, 3H), 6.58 (d, *J* = 16.0 Hz, 1H), 5.29 (*t*, *J* = 3.7 Hz, 1H), 3.07 − 2.99 (*m*, 1H), 2.86 − 2.79 (*m*, 1H), 2.37 (ddd, *J* = 15.0, 12.2, 5.8 Hz, 1H), 1.97 (qd, *J* = 15.6, 14.7, 3.6 Hz, 2H), 1.91 − 1.82 (*m*, 1H), 1.82 − 1.66 (*m*, 3H), 1.64 − 1.55 (*m*, 5H), 1.51 − 1.42 (*m*, 2H), 1.35 (*s*, 2H), 1.32 (*s*, 3H), 1.24 (d, *J* = 10.0 Hz, 1H), 1.22 − 1.07 (*m*, 9H), 1.05 (*s*, 3H), 0.91 (d, *J* = 12.2 Hz, 7H), 0.80 (*s*, 3H). ^13 ^C NMR (126 MHz, Chloroform-*d*) *δ* 183.51, 175.45, 165.31, 145.48, 143.77, 134.48, 130.46, 128.94, 128.20, 122.33, 116.35, 55.86, 47.18, 46.57, 45.81, 41.71, 41.53, 41.02, 39.33, 38.75, 37.07, 33.79, 33.07, 32.36 (d, *J* = 9.5 Hz), 30.69, 27.64, 27.09, 25.88, 23.53 (d, *J* = 13.2 Hz), 23.16, 22.91, 19.64, 18.95, 17.07, 15.16. HRMS (ESI-MS) *m*/*z*: [M + H]^+^ calcd for C_39_H_53_NO_4_: 600.4047; found: 600.4026.

**8b** (C_40_H_55_NO_4_). White powder; Yield 60%. ^1^H NMR (500 MHz, Chloroform-*d*) *δ* 7.75 (d, *J* = 16.0 Hz, 1H), 7.45 (d, *J* = 7.9 Hz, 2H), 7.20 (d, *J* = 7.8 Hz, 2H), 6.53 (d, *J* = 16.0 Hz, 1H), 5.29 (*t*, *J* = 3.7 Hz, 1H), 3.03 (dt, *J* = 15.2, 4.5 Hz, 1H), 2.82 (dd, *J* = 14.0, 4.6 Hz, 1H), 2.38 (*s*, 4H), 2.42 − 2.32 (*m*, 1H), 2.03 − 1.82 (*m*, 4H), 1.82 − 1.72 (*m*, 2H), 1.72 − 1.55 (*m*, 5H), 1.47 (ddt, *J* = 18.4, 13.5, 7.8 Hz, 2H), 1.35 (ddd, *J* = 13.0, 9.0, 3.2 Hz, 3H), 1.30 − 1.21 (*m*, 2H), 1.21 − 1.07 (*m*, 9H), 1.05 (*s*, 3H), 0.99 − 0.85 (*m*, 7H), 0.80 (*s*, 3H). ^13 ^C NMR (126 MHz, Chloroform-*d*) *δ* 183.36, 175.33, 165.51, 145.50, 143.77, 140.92, 131.75, 129.67, 128.20, 122.33, 115.21, 55.86, 47.17, 46.56, 45.82, 41.72, 41.52, 41.03, 39.33, 38.75, 37.07, 33.80, 33.08, 32.37 (d, *J* = 9.1 Hz), 30.69, 27.64, 27.09, 25.88, 23.53 (d, *J* = 13.5 Hz), 23.17, 22.92, 21.53, 19.62, 18.95, 17.06, 15.16. HRMS (ESI-MS) *m*/*z*: [M + K]^+^ calcd for C_40_H_55_NO_4_: 652.3763; found: 652.3740.

**8c** (C_40_H_55_NO_5_). White powder; Yield 70%. ^1^H NMR (500 MHz, Chloroform-*d*) *δ* 7.73 (d, *J* = 15.9 Hz, 1H), 7.54 − 7.47 (*m*, 2H), 6.94 − 6.88 (*m*, 2H), 6.44 (d, *J* = 15.9 Hz, 1H), 5.29 (*t*, *J* = 3.7 Hz, 1H), 3.84 (*s*, 3H), 3.03 (ddd, *J* = 15.2, 5.2, 3.6 Hz, 1H), 2.82 (dd, *J* = 14.2, 4.6 Hz, 1H), 2.36 (ddd, *J* = 15.2, 12.3, 5.9 Hz, 1H), 2.00 (dd, *J* = 13.4, 3.8 Hz, 1H), 1.98 − 1.91 (*m*, 1H), 1.87 (ddd, *J* = 18.3, 6.8, 4.1 Hz, 1H), 1.82 − 1.66 (*m*, 3H), 1.66 − 1.54 (*m*, 3H), 1.51 − 1.41 (*m*, 2H), 1.44 − 1.31 (*m*, 3H), 1.31 (*s*, 3H), 1.26 (*s*, 2H), 1.22 (d, *J* = 29.2 Hz, 2H), 1.16 (*s*, 3H), 1.12 (d, *J* = 2.5 Hz, 4H), 1.05 (*s*, 3H), 0.91 (d, *J* = 12.2 Hz, 7H), 0.80 (*s*, 3H). ^13 ^C NMR (126 MHz, Chloroform-*d*) *δ* 183.23, 175.21, 165.70, 161.51, 145.17, 143.77, 129.89, 127.24, 122.34, 114.35, 113.71, 113.47, 55.86, 55.43, 47.17, 46.55, 45.81, 41.72, 41.50, 41.03, 39.32, 37.07, 33.79, 33.07, 32.36 (d, *J* = 8.5 Hz), 30.69, 27.64, 27.08, 25.87, 23.53 (d, *J* = 13.0 Hz), 23.16, 19.60, 18.94, 17.06, 15.15. HRMS (ESI-MS) m/z: [M + H]^+^ calcd for C_40_H_55_NO_5_: 630.4153; found: 630.4124.

**8d** (C_39_H_52_FNO_4_). Light yellow powder; Yield 55%. ^1^H NMR (500 MHz, Chloroform-*d*) *δ* 7.73 (d, *J* = 15.9 Hz, 1H), 7.54 (dd, *J* = 8.5, 5.4 Hz, 2H), 7.08 (*t*, *J* = 8.4 Hz, 2H), 6.50 (d, *J* = 15.9 Hz, 1H), 5.29 (d, *J* = 3.6 Hz, 1H), 3.02 (dt, *J* = 15.3, 4.6 Hz, 1H), 2.82 (dd, *J* = 13.9, 4.6 Hz, 1H), 2.37 (ddd, *J* = 15.0, 12.2, 5.8 Hz, 1H), 2.02 − 1.91 (*m*, 2H), 1.87 (ddd, *J* = 18.5, 6.7, 4.0 Hz, 1H), 1.74 (dtd, *J* = 27.9, 13.6, 4.4 Hz, 3H), 1.59 (dd, *J* = 15.2, 10.1 Hz, 5H), 1.47 (ddt, *J* = 18.0, 13.3, 7.5 Hz, 2H), 1.35 (q, *J* = 5.2 Hz, 2H), 1.31 (*s*, 3H), 1.24 (dd, *J* = 11.7, 4.4 Hz, 1H), 1.21 − 1.07 (*m*, 9H), 1.05 (*s*, 3H), 0.91 (d, *J* = 12.1 Hz, 7H), 0.79 (*s*, 3H). ^13 ^C NMR (126 MHz, Chloroform-*d*) *δ* 183.59, 175.51, 165.10 (d, *J* = 25.8 Hz), 144.18, 143.79, 130.73 (d, *J* = 3.6 Hz), 130.09 (d, *J* = 8.3 Hz), 122.31, 116.19, 116.05 (d, *J* = 8.7 Hz), 55.85, 47.17, 46.56, 45.81, 41.71, 41.54, 41.01, 39.32, 38.74, 37.07, 33.78, 33.07, 32.36 (d, *J* = 10.7 Hz), 30.69, 27.64, 27.09, 25.88, 23.53 (d, *J* = 13.0 Hz), 23.02 (d, *J* = 31.5 Hz), 19.65, 18.94, 17.07, 15.16. HRMS (ESI-MS) *m*/*z*: [M + Na]^+^ calcd for C_39_H_52_FNO_4_: 640.3773; found: 640.3751.

**8e** (C_39_H_52_ClNO_4_). Yellow powder; Yield 63%. ^1^H NMR (500 MHz, Chloroform-*d*) *δ* 7.71 (d, *J* = 16.0 Hz, 1H), 7.48 (d, *J* = 8.2 Hz, 2H), 7.36 (d, *J* = 8.2 Hz, 2H), 6.54 (d, *J* = 16.0 Hz, 1H), 5.28 (*t*, *J* = 3.7 Hz, 1H), 3.01 (dt, *J* = 15.1, 4.5 Hz, 1H), 2.82 (dd, *J* = 13.9, 4.6 Hz, 1H), 2.37 (ddd, *J* = 15.0, 12.2, 5.8 Hz, 1H), 2.03 − 1.90 (*m*, 2H), 1.86 (ddd, *J* = 18.4, 6.7, 4.0 Hz, 1H), 1.76 (ddd, *J* = 14.1, 9.5, 4.6 Hz, 2H), 1.69 (dd, *J* = 13.8, 4.2 Hz, 1H), 1.59 (ddd, *J* = 17.3, 9.0, 3.6 Hz, 5H), 1.46 (dd, *J* = 10.6, 6.5 Hz, 2H), 1.33 (d, *J* = 18.7 Hz, 6H), 1.24 (d, *J* = 11.1 Hz, 1H), 1.14 (d, *J* = 17.7 Hz, 8H), 1.05 (*s*, 3H), 0.91 (d, *J* = 12.0 Hz, 7H), 0.79 (*s*, 3H). ^13 ^C NMR (126 MHz, Chloroform-*d*) *δ* 184.19, 175.57, 165.07, 143.91 (d, *J* = 29.5 Hz), 136.36, 132.95, 129.29 (d, *J* = 17.5 Hz), 122.29, 116.93, 55.84, 47.17, 46.58, 45.81, 41.62 (d, *J* = 17.7 Hz), 40.97, 39.32, 38.73, 37.07, 33.78, 33.08, 32.35 (d, *J* = 13.0 Hz), 30.69, 29.71 (d, *J* = 5.6 Hz), 27.64, 27.09, 25.89, 23.53 (d, *J* = 14.7 Hz), 23.14, 22.87, 19.65, 18.94, 17.07, 15.16. HRMS (ESI-MS) *m*/*z*: [M + Na]^+^ calcd for C_39_H_52_ClNO_4_: 656.3477; found: 656.3451.

**8f** (C_39_H_52_BrNO_4_). White powder; Yield 42%. ^1^H NMR (500 MHz, Chloroform-*d*) *δ* 7.70 (d, *J* = 16.0 Hz, 1H), 7.53 (dd, *J* = 8.7, 2.4 Hz, 2H), 7.46 − 7.39 (m, 2H), 6.56 (d, *J* = 16.0 Hz, 1H), 5.29 (d, *J* = 3.6 Hz, 1H), 3.01 (dt, *J* = 15.1, 4.6 Hz, 1H), 2.82 (dd, *J* = 14.3, 4.9 Hz, 1H), 2.37 (ddd, *J* = 14.8, 12.2, 5.8 Hz, 1H), 2.03 − 1.91 (*m*, 2H), 1.86 (ddd, *J* = 18.4, 6.5, 3.9 Hz, 1H), 1.81 − 1.66 (*m*, 3H), 1.60 (qd, *J* = 11.9, 10.6, 5.5 Hz, 5H), 1.47 (dd, *J* = 10.1, 5.7 Hz, 1H), 1.43 − 1.33 (*m*, 3H), 1.31 (d, *J* = 5.1 Hz, 3H), 1.26 (d, *J* = 15.7 Hz, 1H), 1.24 − 1.18 (*m*, 1H), 1.16 (d, *J* = 3.5 Hz, 5H), 1.12 (*s*, 3H), 1.05 (*s*, 3H), 0.97 (d, *J* = 7.7 Hz, 1H), 0.91 (d, *J* = 12.2 Hz, 6H), 0.80 (d, *J* = 3.6 Hz, 3H). ^13 ^C NMR (126 MHz, Chloroform-*d*) *δ* 182.97, 175.63, 165.06, 144.10, 143.80, 133.37, 132.19, 124.72, 122.30, 117.05, 55.84, 47.17, 46.55, 45.82, 41.73, 41.55, 41.05, 39.32, 38.74, 37.07, 33.79, 33.07, 32.39, 30.69, 27.64, 27.09, 25.87, 23.53 (d, *J* = 13.0 Hz), 23.15, 22.92, 19.66, 18.95, 17.05, 15.16. HRMS (ESI-MS) *m*/*z*: [M + H]^+^ calcd for C_39_H_52_BrNO_4_: 678.3152; found: 678.3130.

**8g** (C_40_H_52_F_3_NO_4_). White powder; Yield 46%. ^1^H NMR (500 MHz, Chloroform-*d*) *δ* 7.78 (dd, *J* = 16.0, 1.6 Hz, 1H), 7.65 (d, *J* = 1.5 Hz, 4H), 6.64 (dd, *J* = 16.0, 1.6 Hz, 1H), 5.29 (*s*, 1H), 3.02 (dd, *J* = 15.4, 4.7 Hz, 1H), 2.83 (dd, *J* = 13.9, 4.6 Hz, 1H), 2.44 − 2.33 (*m*, 1H), 1.97 (dt, *J* = 18.5, 12.1 Hz, 3H), 1.91 − 1.84 (*m*, 1H), 1.74 (ddt, *J* = 31.2, 16.6, 8.3 Hz, 3H), 1.60 (dd, *J* = 9.9, 5.4 Hz, 5H), 1.53 − 1.41 (*m*, 2H), 1.33 (d, *J* = 12.7 Hz, 6H), 1.17 (*s*, 4H), 1.13 (*s*, 3H), 1.05 (*s*, 3H), 0.99 − 0.85 (*m*, 7H), 0.80 (*s*, 3H). ^13 ^C NMR (126 MHz, Chloroform-*d*) *δ* 205.76, 184.26, 178.48, 175.78, 174.94, 164.65 (d, *J* = 20.9 Hz), 143.69 (d, *J* = 30.1 Hz), 137.79, 132.10 − 131.47 (*m*), 128.33 (d, *J* = 3.2 Hz), 125.91 (*t*, *J* = 3.8 Hz), 124.91, 122.74, 122.25, 118.98, 118.81, 91.03, 55.82, 55.33, 50.68, 47.17, 46.56, 45.81, 44.10, 43.97, 43.71, 42.50, 41.65 (d, *J* = 4.8 Hz), 41.56, 40.95, 39.31, 38.73 (d, *J* = 5.2 Hz), 37.50, 37.29, 37.06, 34.12, 33.78, 33.12 (d, *J* = 13.8 Hz), 32.53 − 32.23 (*m*), 31.58, 30.68, 27.63, 27.28 − 26.91 (*m*), 25.86 (d, *J* = 4.1 Hz), 23.77, 23.52 (d, *J* = 15.0 Hz), 23.13, 22.82 (d, *J* = 8.4 Hz), 20.65, 19.60 (d, *J* = 20.3 Hz), 18.93, 18.36, 18.09 (d, *J* = 13.2 Hz), 17.07, 15.70, 15.14. HRMS (ESI-MS) m/z: [M + K]^+^ calcd for C_40_H_52_F_3_NO_4_: 706.3480; found: 706.3448.

**8h** (C_39_H_52_N_2_O_6_). Yellow powder; Yield 55%. ^1^H NMR (500 MHz, Chloroform-*d*) *δ* 8.29 − 8.23 (*m*, 2H), 7.79 (d, *J* = 16.0 Hz, 1H), 7.70 (dd, *J* = 8.9, 2.7 Hz, 2H), 6.73 − 6.64 (*m*, 1H), 5.30 − 5.25 (*m*, 1H), 3.00 (dt, *J* = 15.1, 4.7 Hz, 1H), 2.82 (dd, *J* = 13.8, 4.6 Hz, 1H), 2.39 (ddd, *J* = 15.3, 12.3, 6.0 Hz, 1H), 2.02 − 1.91 (*m*, 2H), 1.89 − 1.76 (*m*, 1H), 1.75 − 1.66 (*m*, 1H), 1.64 − 1.55 (*m*, 6H), 1.47 (dd, *J* = 10.5, 6.1 Hz, 1H), 1.44 − 1.29 (*m*, 7H), 1.26 (d, *J* = 16.3 Hz, 2H), 1.17 (d, *J* = 12.8 Hz, 5H), 1.12 (*s*, 3H), 1.05 (*s*, 3H), 1.01 − 0.94 (*m*, 1H), 0.91 (d, *J* = 12.0 Hz, 6H), 0.79 (*s*, 3H). ^13 ^C NMR (126 MHz, Chloroform-*d*) *δ* 184.08, 176.01, 164.35, 148.58, 143.82, 142.46, 140.53, 128.78, 124.22, 122.25, 120.76, 55.83, 47.18, 46.57, 45.82, 41.65 (d, *J* = 11.1 Hz), 40.98, 39.32, 38.73, 37.07, 33.78, 33.07, 32.34 (d, *J* = 13.2 Hz), 30.69, 29.72, 27.63, 27.12, 25.88, 23.52 (d, *J* = 13.8 Hz), 23.13, 22.87, 19.73, 18.95, 17.06, 15.17. HRMS (ESI-MS) *m*/*z*: [M + Na]^+^ calcd for C_39_H_52_N_2_O_6_: 667.3718; found: 667.3717.

**8i** (C_40_H_53_NO_6_). White powder; Yield 45%. ^1^H NMR (500 MHz, Chloroform-*d*) *δ* 7.68 (d, *J* = 15.9 Hz, 1H), 7.09 − 7.00 (*m*, 2H), 6.82 (d, *J* = 8.1 Hz, 1H), 6.40 (d, *J* = 15.9 Hz, 1H), 6.01 (*s*, 2H), 5.28 (t, *J* = 3.6 Hz, 1H), 3.02 (dt, *J* = 15.1, 4.5 Hz, 1H), 2.82 (dd, *J* = 13.7, 4.7 Hz, 1H), 2.36 (ddd, *J* = 15.1, 12.3, 6.1 Hz, 1H), 1.97 (qd, *J* = 15.2, 14.4, 3.6 Hz, 2H), 1.86 (ddd, *J* = 18.5, 6.5, 3.8 Hz, 1H), 1.81 − 1.66 (*m*, 3H), 1.61 − 1.55 (*m*, 1H), 1.53 − 1.45 (*m*, 1H), 1.44 − 1.34 (*m*, 1H), 1.37 − 1.24 (*m*, 9H), 1.16 (d, *J* = 5.5 Hz, 7H), 1.12 (*s*, 3H), 1.05 (*s*, 3H), 0.91 (d, *J* = 12.3 Hz, 7H), 0.79 (*s*, 3H). ^13 ^C NMR (126 MHz, Chloroform-*d*) *δ* 183.61, 175.29, 165.57, 149.75, 148.38, 145.22, 143.77, 128.93, 124.72, 122.33, 114.16, 108.59, 106.54, 101.62, 55.85, 47.17, 46.57, 45.81, 41.71, 41.51, 41.02, 39.32, 38.74, 37.07, 33.79, 33.08, 32.36 (d, *J* = 9.9 Hz), 30.69, 29.73, 27.63, 27.08, 25.88, 23.53 (d, *J* = 14.0 Hz), 23.03 (d, *J* = 31.3 Hz), 19.61, 18.94, 17.04, 15.15. HRMS (ESI-MS) *m*/*z*: [M + Na]^+^ calcd for C_40_H_53_NO_6_: 666.3765; found: 666.3736.

**8j** (C_39_H_53_NO_5_). White powder; Yield 40%. ^1^H NMR (500 MHz, Chloroform-*d*) *δ* 7.73 (d, *J* = 15.9 Hz, 1H), 7.43 (d, *J* = 8.3 Hz, 2H), 6.89 (d, *J* = 8.3 Hz, 2H), 6.42 (d, *J* = 15.9 Hz, 1H), 5.27 (d, *J* = 3.8 Hz, 1H), 2.99 (dt, *J* = 15.3, 4.8 Hz, 1H), 2.82 (dd, *J* = 13.9, 4.6 Hz, 1H), 2.40 (ddd, *J* = 15.3, 12.1, 6.2 Hz, 1H), 2.03 − 1.82 (*m*, 3H), 1.73 (ddt, *J* = 25.4, 15.8, 8.4 Hz, 3H), 1.60 (*q*, *J* = 11.3, 9.5 Hz, 6H), 1.50 − 1.29 (*m*, 3H), 1.28 − 1.06 (*m*, 12H), 1.02 (*s*, 3H), 0.98 − 0.86 (*m*, 8H), 0.77 (*s*, 3H). ^13 ^C NMR (126 MHz, Chloroform-*d*) *δ* 184.23, 175.58, 166.74, 158.90, 146.21, 143.74, 130.19, 126.56, 122.37, 116.16, 112.68, 55.62, 47.11, 46.59, 45.78, 41.67, 41.46, 40.99, 39.30, 38.59, 37.04, 33.77, 33.09, 32.34 (d, *J* = 23.0 Hz), 30.69, 27.63, 27.19, 25.90, 23.52 (d, *J* = 16.9 Hz), 22.98 (d, *J* = 32.1 Hz), 19.70, 18.96, 17.09, 15.18. HRMS (ESI-MS) m/z: [M + K]^+^ calcd for C_39_H_53_NO_5_: 654.3555; found: 654.3525.

**8k** (C_37_H_51_NO_4_). White powder; Yield 70%. ^1^H NMR (500 MHz, Chloroform-*d*) *δ* 8.08 − 8.03 (*m*, 2H), 7.58 (td, *J* = 7.3, 1.3 Hz, 1H), 7.46 (*t*, *J* = 7.8 Hz, 2H), 5.29 (d, *J* = 3.8 Hz, 1H), 3.11 − 3.03 (*m*, 1H), 2.83 (dd, *J* = 13.9, 4.6 Hz, 1H), 2.44 (ddd, *J* = 15.2, 12.2, 5.9 Hz, 1H), 1.97 (qd, *J* = 14.9, 14.2, 3.6 Hz, 2H), 1.90 − 1.82 (*m*, 1H), 1.75 (ddt, *J* = 28.0, 13.9, 4.9 Hz, 3H), 1.62 (td, *J* = 12.2, 10.2, 5.1 Hz, 5H), 1.54 − 1.44 (*m*, 2H), 1.35 (*s*, 6H), 1.26 − 1.20 (*m*, 3H), 1.13 (d, *J* = 5.3 Hz, 5H), 1.07 (*s*, 3H), 0.91 (d, *J* = 12.1 Hz, 7H), 0.81 (*s*, 3H). ^13 ^C NMR (126 MHz, Chloroform-*d*) *δ* 183.76, 176.24, 164.32, 143.78, 133.04, 129.54, 128.50, 122.31, 55.82, 47.16, 46.58, 45.80, 41.67 (d, *J* = 10.9 Hz), 41.01, 39.33, 38.74, 37.09, 33.79, 33.07, 32.36 (d, *J* = 10.6 Hz), 30.69, 27.64, 27.14, 25.89, 23.53 (d, *J* = 13.9 Hz), 23.21, 22.89, 19.88, 18.95, 17.07, 15.16. HRMS (ESI-MS) *m*/*z*: [M + K]^+^ calcd for C_37_H_51_NO_4_: 612.3450; found: 612.3423.

**8l** (C_38_H_53_NO_4_). White powder; Yield 68%. ^1^H NMR (500 MHz, Chloroform-*d*) *δ* 7.93 (d, *J* = 8.1 Hz, 2H), 7.23 (d, *J* = 8.0 Hz, 2H), 5.26 (d, *J* = 3.9 Hz, 1H), 3.06 (dt, *J* = 15.3, 4.6 Hz, 1H), 2.82 (dd, *J* = 14.0, 4.6 Hz, 1H), 2.40 (*s*, 4H), 2.01 − 1.90 (*m*, 2H), 1.90 − 1.81 (*m*, 1H), 1.74 (tdd, *J* = 20.0, 18.2, 9.8, 3.9 Hz, 3H), 1.59 (td, *J* = 17.0, 14.9, 8.6 Hz, 5H), 1.51 − 1.40 (*m*, 2H), 1.34 (*s*, 5H), 1.24 (*s*, 1H), 1.18 (d, *J* = 5.2 Hz, 5H), 1.11 (*s*, 4H), 1.05 (*s*, 3H), 0.90 (d, *J* = 12.3 Hz, 7H), 0.79 (*s*, 3H). ^13 ^C NMR (126 MHz, Chloroform-*d*) *δ* 184.64, 176.00, 164.39, 143.76 (d, *J* = 5.5 Hz), 129.57, 129.22, 126.86, 122.30, 55.81, 47.15, 46.58, 45.80, 41.63 (d, *J* = 10.8 Hz), 40.95, 39.32, 38.71, 37.08, 33.79, 33.10, 32.36 (d, *J* = 14.3 Hz), 30.68, 27.63, 27.15, 25.91, 23.53 (d, *J* = 17.4 Hz), 23.22, 22.86, 21.75, 19.85, 18.94, 17.07, 15.14. HRMS (ESI-MS) *m*/*z*: [M + H]^+^ calcd for C_38_H_53_NO_4_: 588.4047; found: 588.4018.

**8m** (C_38_H_53_NO_5_). Yellow powder; Yield 44%. ^1^H NMR (500 MHz, Chloroform-*d*) *δ* 8.07 − 7.97 (m, 2H), 6.94 (dd, *J* = 9.2, 2.4 Hz, 2H), 5.28 (*t*, *J* = 3.7 Hz, 1H), 3.87 (*s*, 3H), 3.11 − 3.02 (*m*, 1H), 2.82 (dd, *J* = 14.0, 4.6 Hz, 1H), 2.47 − 2.35 (*m*, 1H), 2.03 − 1.83 (*m*, 3H), 1.81 − 1.70 (*m*, 3H), 1.62 (ddd, *J* = 12.7, 9.6, 5.1 Hz, 5H), 1.51 − 1.30 (*m*, 8H), 1.25 (d, *J* = 2.2 Hz, 2H), 1.19 − 1.10 (*m*, 7H), 1.06 (*s*, 3H), 0.92 (dd, *J* = 11.1, 5.3 Hz, 7H), 0.80 (*s*, 3H). ^13 ^C NMR (126 MHz, Chloroform-*d*) *δ* 183.58, 175.85, 164.09, 163.42, 143.78, 131.58, 122.32, 121.96, 113.77, 55.82, 55.48, 47.15, 46.57, 45.80, 41.65 (d, *J* = 17.4 Hz), 41.02, 39.33, 38.73, 37.08, 33.79, 33.08, 32.36 (d, *J* = 9.5 Hz), 30.69, 27.63, 27.13, 25.88, 23.53 (d, *J* = 14.5 Hz), 23.22, 22.90, 19.83, 18.94, 17.07, 15.14. HRMS (ESI-MS) *m*/*z*: [M + H]^+^ calcd for C_38_H_53_NO_5_: 604.3997; found: 604.3997.

**8n** (C_37_H_50_FNO_4_). White powder; Yield 56%. ^1^H NMR (500 MHz, Chloroform-*d*) *δ* 8.10 − 8.03 (*m*, 2H), 7.13 (*t*, *J* = 8.6 Hz, 2H), 5.28 (*t*, *J* = 3.7 Hz, 1H), 3.04 (ddd, *J* = 15.3, 5.4, 3.7 Hz, 1H), 2.82 (dd, *J* = 13.8, 4.6 Hz, 1H), 2.44 (ddd, *J* = 15.2, 12.2, 5.9 Hz, 1H), 2.03 − 1.91 (*m*, 2H), 1.90 − 1.67 (*m*, 4H), 1.65 − 1.55 (*m*, 5H), 1.47 (qd, *J* = 14.1, 13.2, 7.4 Hz, 2H), 1.39 − 1.30 (*m*, 5H), 1.27 − 1.20 (*m*, 2H), 1.18 (*s*, 3H), 1.17 − 1.09 (*m*, 4H), 1.06 (*s*, 4H), 0.91 (d, *J* = 11.9 Hz, 7H), 0.79 (*s*, 3H). ^13 ^C NMR (126 MHz, Chloroform-*d*) *δ* 184.23, 176.31, 166.81, 164.78, 163.38, 143.80, 132.07 (d, *J* = 9.2 Hz), 125.89 (d, *J* = 3.4 Hz), 122.28, 115.71 (d, *J* = 22.0 Hz), 55.79, 47.15, 46.58, 45.80, 41.67 (d, *J* = 6.6 Hz), 40.97, 39.32, 38.71, 37.08, 33.78, 33.07, 32.35 (d, *J* = 13.4 Hz), 30.69, 27.63, 27.15, 25.89, 23.52 (d, *J* = 14.7 Hz), 23.20, 22.87, 19.90, 18.94, 17.07, 15.16. HRMS (ESI-MS) *m*/*z*: [M + K]^+^ calcd for C_37_H_50_FNO_4_: 630.3355; found: 630.3326.

**8o** (C_37_H_50_ClNO_4_). White powder; Yield 65%. ^1^H NMR (500 MHz, Chloroform-*d*) *δ* 8.01 − 7.96 (m, 2H), 7.46 − 7.40 (*m*, 2H), 5.28 (*q*, *J* = 3.9 Hz, 1H), 3.03 (ddd, *J* = 15.3, 5.4, 3.7 Hz, 1H), 2.82 (dd, *J* = 13.8, 4.7 Hz, 1H), 2.43 (ddd, *J* = 15.2, 12.2, 5.9 Hz, 1H), 2.03 − 1.90 (*m*, 2H), 1.86 (ddd, *J* = 18.3, 6.5, 3.9 Hz, 1H), 1.82 − 1.67 (*m*, 3H), 1.66 − 1.54 (*m*, 6H), 1.53 − 1.42 (*m*, 2H), 1.41 − 1.30 (*m*, 6H), 1.29 − 1.20 (*m*, 2H), 1.18 (*s*, 3H), 1.12 (*s*, 3H), 1.06 (*s*, 3H), 0.91 (d, *J* = 11.9 Hz, 7H), 0.79 (*s*, 3H). ^13 ^C NMR (126 MHz, Chloroform-*d*) *δ* 184.13, 176.45, 163.49, 143.80, 139.50, 130.92, 128.89, 128.12, 122.27, 55.79, 47.15, 46.58, 45.80, 41.68 (d, *J* = 3.8 Hz), 40.98, 39.32, 38.70, 37.08, 33.78, 33.08, 32.35 (d, *J* = 13.5 Hz), 30.69, 27.63, 27.15, 25.89, 23.52 (d, *J* = 14.8 Hz), 23.20, 22.87, 19.92, 18.94, 17.07, 15.16. HRMS (ESI-MS) *m*/*z*: [M + H]^+^ calcd for C_37_H_50_ClNO_4_: 608.3501; found: 608.3482.

**8p** (C_37_H_50_BrNO_4_). White powder; Yield 42%. ^1^H NMR (500 MHz, Chloroform-*d*) *δ* 7.93 − 7.88 (*m*, 2H), 7.62 − 7.56 (*m*, 2H), 5.28 (*q*, *J* = 3.8 Hz, 1H), 3.02 (ddd, *J* = 15.3, 5.3, 3.7 Hz, 1H), 2.82 (dd, *J* = 13.8, 4.6 Hz, 1H), 2.43 (ddd, *J* = 15.1, 12.1, 5.9 Hz, 1H), 2.03 − 1.91 (*m*, 2H), 1.86 (ddd, *J* = 18.3, 6.6, 4.0 Hz, 1H), 1.82 − 1.67 (*m*, 3H), 1.67 − 1.54 (*m*, 5H), 1.53 − 1.39 (*m*, 3H), 1.38 − 1.30 (*m*, 5H), 1.26 − 1.20 (*m*, 2H), 1.18 (*s*, 3H), 1.12 (*s*, 3H), 1.08 (*s*, 1H), 1.06 (*s*, 3H), 0.91 (d, *J* = 11.8 Hz, 7H), 0.79 (d, *J* = 3.6 Hz, 3H). ^13 ^C NMR (126 MHz, Chloroform-*d*) *δ* 184.26, 176.46, 163.60, 143.78, 131.86, 131.03, 128.57, 128.15, 122.26, 55.79, 47.15, 46.58, 45.80, 41.67 (d, *J* = 3.0 Hz), 40.98 (d, *J* = 4.0 Hz), 39.32, 38.70, 37.08, 33.78, 33.08, 32.34 (d, *J* = 13.8 Hz), 30.69, 27.63, 27.14, 25.89, 23.52 (d, *J* = 15.2 Hz), 23.20, 22.86, 19.93, 18.94, 17.07, 15.15. HRMS (ESI-MS) *m*/*z*: [M + K]^+^ calcd for C_37_H_50_BrNO_4_: 690.2555; found: 690.2524.

**8q** (C_38_H_50_F_3_NO_4_). White powder; Yield 43%. ^1^H NMR (500 MHz, Chloroform-*d*) *δ* 8.16 (d, *J* = 8.1 Hz, 2H), 7.72 (d, *J* = 8.2 Hz, 2H), 5.28 (*t*, *J* = 3.6 Hz, 1H), 3.08 − 2.99 (*m*, 1H), 2.82 (dd, *J* = 13.9, 4.6 Hz, 1H), 2.46 (ddd, *J* = 15.2, 12.1, 5.9 Hz, 1H), 2.03 − 1.91 (*m*, 2H), 1.90 − 1.75 (*m*, 2H), 1.75 − 1.67 (*m*, 1H), 1.60 (ddt, *J* = 18.0, 13.5, 6.8 Hz, 5H), 1.53 − 1.40 (*m*, 2H), 1.35 (*s*, 5H), 1.29 − 1.22 (*m*, 3H), 1.20 (*s*, 3H), 1.17 − 1.04 (*m*, 8H), 0.91 (d, *J* = 12.0 Hz, 7H), 0.80 (*s*, 3H). ^13 ^C NMR (126 MHz, Chloroform-*d*) *δ* 184.40, 176.83, 163.14, 143.80, 134.52 (d, *J* = 32.3 Hz), 132.99, 129.93, 126.11 − 125.38 (*m*), 122.25, 55.80, 47.16, 46.59, 45.80, 41.70 (d, *J* = 4.6 Hz), 40.96, 39.32, 38.70, 37.09, 33.78, 33.07, 32.34 (d, *J* = 14.6 Hz), 30.69, 27.63, 27.15, 25.89, 23.52 (d, *J* = 14.6 Hz), 23.19, 22.86, 19.98, 18.94, 17.07, 15.17. HRMS (ESI-MS) *m*/*z*: [M + H]^+^ calcd for C_38_H_50_F_3_NO_4_: 642.3765; found: 642.3736.

**8r** (C_37_H_50_N_2_O_6_). Yellow powder; Yield 57%. ^1^H NMR (500 MHz, Chloroform-*d*) *δ* 8.31 (d, *J* = 8.5 Hz, 2H), 8.22 (d, *J* = 8.6 Hz, 2H), 5.28 (*t*, *J* = 3.6 Hz, 1H), 3.03 (dt, *J* = 15.2, 4.7 Hz, 1H), 2.82 (dd, *J* = 13.8, 4.6 Hz, 1H), 2.47 (ddd, *J* = 15.1, 12.0, 6.0 Hz, 1H), 1.97 (qd, *J* = 11.8, 3.3 Hz, 2H), 1.90 − 1.78 (*m*, 2H), 1.77 − 1.70 (*m*, 1H), 1.65 − 1.54 (*m*, 5H), 1.51 − 1.40 (*m*, 2H), 1.39 − 1.27 (*m*, 7H), 1.23 − 1.22 (*m*, 1H), 1.20 (*s*, 4H), 1.12 (*s*, 4H), 1.07 (d, *J* = 6.9 Hz, 3H), 0.91 (d, *J* = 12.1 Hz, 7H), 0.80 (*s*, 3H). ^13 ^C NMR (126 MHz, Chloroform-*d*) *δ* 184.33, 177.14, 162.47, 150.56, 143.82, 135.19, 130.65, 123.72, 122.22, 55.78, 47.16, 46.58, 45.81, 41.73 (d, *J* = 10.1 Hz), 40.96, 39.32, 38.69, 37.09, 33.78, 33.07, 32.33 (d, *J* = 15.3 Hz), 30.69, 29.71 (d, *J* = 5.3 Hz), 27.63, 27.18, 25.89, 23.52 (d, *J* = 14.7 Hz), 23.20, 22.79 (d, *J* = 17.3 Hz), 20.07, 18.95, 17.05, 15.18. HRMS (ESI-MS) *m*/*z*: [M + H]^+^ calcd for C_37_H_50_N_2_O_6_: 619.3742; found: 619.3724.

**8s** (C_38_H_51_NO_6_). White powder; Yield 44%. ^1^H NMR (500 MHz, Chloroform-*d*) *δ* 7.66 (dd, *J* = 8.1, 1.7 Hz, 1H), 7.48 (d, *J* = 1.7 Hz, 1H), 6.86 (d, *J* = 8.2 Hz, 1H), 6.05 (*s*, 2H), 5.29 (*t*, *J* = 3.7 Hz, 1H), 3.04 (ddd, *J* = 15.3, 5.4, 3.7 Hz, 1H), 2.83 (dd, *J* = 13.8, 4.6 Hz, 1H), 2.42 (ddd, *J* = 15.2, 12.2, 5.9 Hz, 1H), 2.05 − 1.92 (*m*, 2H), 1.90 − 1.83 (*m*, 1H), 1.82 − 1.73 (*m*, 2H), 1.72 − 1.67 (*m*, 1H), 1.66 − 1.56 (*m*, 6H), 1.53 − 1.35 (*m*, 3H), 1.33 (*s*, 3H), 1.22 − 1.16 (*m*, 5H), 1.13 (*s*, 4H), 1.06 (*s*, 4H), 0.91 (d, *J* = 12.1 Hz, 7H), 0.80 (*s*, 3H). ^13 ^C NMR (126 MHz, Chloroform-*d*) *δ* 182.65, 175.97, 163.70, 151.70, 147.79, 143.77, 125.33, 123.53, 122.33, 109.45, 108.15, 101.87, 55.82, 47.15, 46.54, 45.81, 41.74, 41.59, 41.08, 39.32, 38.73, 37.07, 33.79, 33.07, 32.36 (d, *J* = 7.6 Hz), 30.70, 29.73, 27.64, 27.14, 25.87, 23.52 (d, *J* = 13.0 Hz), 23.22, 22.94, 19.87, 18.96, 17.04, 15.15. HRMS (ESI-MS) *m*/*z*: [M + H]^+^ calcd for C_38_H_51_NO_6_: 618.3789; found: 618.3760.

**8t** (C_37_H_51_NO_5_). Yellow powder; Yield 45%. ^1^H NMR (500 MHz, DMSO-d_6_) *δ* 12.07 (*s*, 1H), 10.41 (*s*, 1H), 7.87 − 7.81 (*m*, 2H), 6.91 − 6.84 (*m*, 2H), 5.20 − 5.15 (*m*, 1H), 2.96 (dt, *J* = 15.2, 4.8 Hz, 1H), 2.74 (dd, *J* = 14.0, 4.5 Hz, 1H), 1.96 − 1.81 (*m*, 2H), 1.69 (dd, *J* = 12.1, 6.3 Hz, 1H), 1.66 − 1.53 (*m*, 3H), 1.51 − 1.40 (*m*, 4H), 1.37 − 1.25 (*m*, 3H), 1.22 (d, *J* = 7.4 Hz, 7H), 1.18 − 1.08 (*m*, 9H), 0.99 (*s*, 3H), 0.87 (*s*, 7H), 0.76 (*s*, 3H). ^13 ^C NMR (126 MHz, DMSO-d_6_) *δ* 179.10, 175.68, 163.52, 162.63, 144.35, 131.88, 121.86, 119.84, 116.03, 55.43, 46.91, 46.02 (d, *J* = 17.4 Hz), 41.90, 41.36 (d, *J* = 11.9 Hz), 38.51, 37.03, 33.77, 33.29, 32.49 (d, *J* = 11.9 Hz), 30.87, 27.67 (d, *J* = 5.7 Hz), 25.95, 23.83, 23.43, 23.07, 19.73, 19.03, 17.22, 15.24. HRMS (ESI-MS) *m*/*z*: [M + K]^+^ calcd for C_37_H_51_NO_5_: 628.3399; found: 628.3378.

### 4.3. α-Glucosidase inhibition and kinetics assay

The *α*-glucosidase inhibition assay of all the synthesised OA derivatives (**3a**–**3t**) was performed according to previously reported method with minor modifications^22–24^. Briefly, *α*-glucosidase (10 µL, final concentration 0.1 U/mL, dissolved in PBS), test compound (10 µL, dissolved in DMSO), and PBS (130 µL, 0.1 M phosphate, pH 6.8) were added into a 96-well plate and the mixture was incubated at 37 °C for 10 min. After *p*-NPG (50 µL, final concentration 0.25 mM) was added, the absorbance of the mixture was measured at 405 nm at every 10 minutes using Microplate reader. DMSO working concentration in enzyme inhibition test was 5%. Acarbose was used as a positive control for *α*-glucosidase inhibition assay. Inhibition rate (%) = [(A_1_ – A_0_)/A_0_] × 100%, where A_1_ and A_0_ represent the absorbance of the blank and test compound, respectively. IC50 value of each compound were obtained from the inhibition curve. All samples were assayed at least in triplicate.

The enzyme inhibition kinetics analysis was investigated using previously reported method with minor modifications^25^^,^^26^. In brief, inhibitory effect was determined from the plots of absorbance change of OA derivatives at different concentrations with enzyme at different concentrations. The inhibition type was measured based on the Lineweaver–Burk plot of absorbance change of OA derivatives at different concentrations with p-NPG substrate at different concentrations.

### 4.4. α-Amylase inhibition and kinetics assay

The *α*-amylase inhibition of OA derivatives (**3a**-**3t**) was also assayed using previously reported method with minor modifications^27–29^. Briefly, *α*-amylase (10 µL, final concentration 0.25 U/mL) and test compound (10 µL) were added into PBS (80 µL, 20 mM phosphate, pH 6.9) and the mixture was incubated at 37 °C for 10 min. After starch solution (100 µL, final concentration 0.5%) was added, the mixture was incubation at 37 °C for 10 min. Then DNS (100 µL, containing 1 M potassium sodium tartrate and 48 mM 3,5-Dinitrosalicylic acid) was added and the mixture was boiled for 15 min. Then, the absorbance was measured at 540 nm. The inhibition rate (%) = [(A_1_ – A_0_)/A_0_] × 100%, where A_1_ and A_0_ represent the absorbance of the blank and test compound, respectively. The IC50 values of all the synthesised compounds were obtained from the inhibition curve. All samples were assayed at least in triplicate.

The enzyme inhibition kinetics analysis was investigated. The operation method was similar with the assay of *α*-glucosidase, except the concentrations of test compound, enzyme and substrate.

### 4.5. Molecular docking

**Figure 6.**
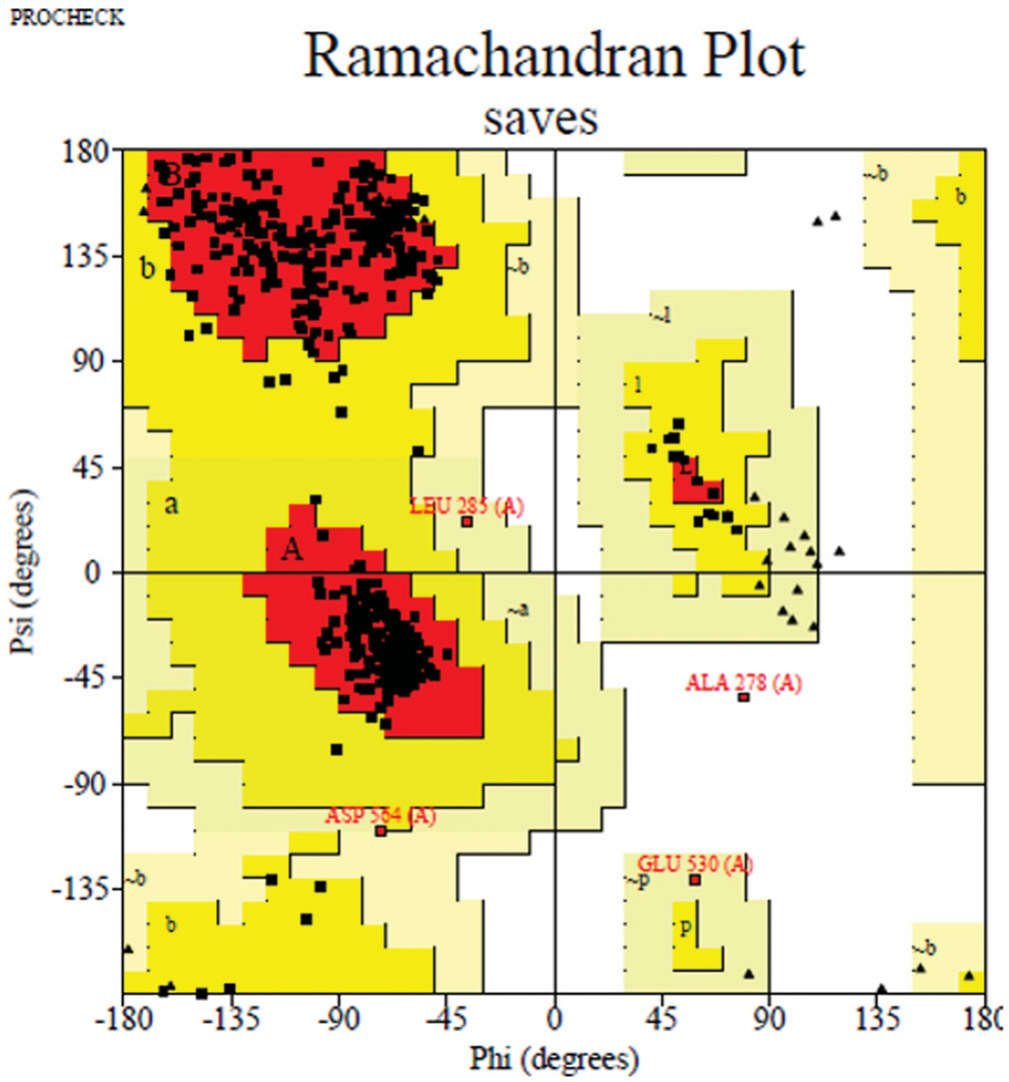
Ramachandran plot results of most reasonable homology mode.

Molecular docking was simulated to show the binding between inhibitor with target protein using Sybyl software (Tripos, US)^21^. The crystal structure of *Saccharomyces cerevisiae isomaltase* has not been reported, then the homology mode of α-glucosidase was constructed. The sequence in FASTA format of α-glucosidase (access code P53341) was obtained from UniProt. *Saccharomyces cerevisiae* isomaltase (PDB ID: 3AJ7) showing 72.4% of sequence identity with α-glucosidase was selected as the template^30–32^. Then, modeller 10.1 software (http://salilab.org/modeller/) was used to establish homology mode and Ramachandran plot (http://services.mbi.ucla.edu/PROCHECK/) was used to verify their quality^33^. The most reasonable homology mode (Figure 6) was used for subsequent docking experiments. The human pancreatic α-amylase (PDB: 3BAJ) were obtained from the Protein Data Bank^34^^,^^35^. The protein was prepared following standard operating procedures and the active pocket was generated using blind or ligand mode, respectively. Compound **3a** for *α*-glucosidase and compound **3f** for *α*-amylase were respectively prepared with energy minimisation program. After that, the molecular docking of between inhibitor and target protein was operated in the default format. The interactions were visualised by Pymol software and Discovery studio software.

### 4.6. Cells cytotoxicity assay

Cells cytotoxicity of the most potent compounds (**3a** and **3f**) on 3T3–L1 and HepG2 cells were evaluated *via* MTT method^36^^,^^37^. 3T3–L1 or HepG2 cells were cultured in Dulbecco’s modified Eagle medium (DMEM) with 10% foetal bovine serum (FBS), 100 IU/mL penicillin and 100 IU/mL streptomycin at 37 °C under 5% CO_2_. 3T3-L1 cells (2 × 10^3^) or HepG2 cells (5 × 10^5^) were plated onto 96-well plate (100 ml/well) and incubated for 24 h. After treated with compound (**3a** or **3f**) for 24 h, the cells were added 100 ml MTT (0.5 mg/mL) and incubated for 4 h. Then 100 ml DMSO was added to dissolve the blue insoluble MTT formazan. Finally, the absorbance was measured at 570 nm using a microplate reader. Each sample was performed in triplicate.

### 4.7. Statistical analysis

All data were presented as mean ± SD. One-way ANOVA was performed to evaluate the difference between groups. *p* < 0.05 was considered significant.

## Supplementary Material

Supplemental Material

## References

[1] Silva FSG, Oliveira PJ, Duarte MF. Oleanolic, ursolic, and betulinic acids as food supplements or pharmaceutical agents for type 2 diabetes: promise or illusion? J Agric Food Chem 2016;64:2991–3008. 27012451 10.1021/acs.jafc.5b06021

[2] Saxena AR, Gorman DN, Esquejo RM, et al. Danuglipron (PF-06882961) in type 2 diabetes: a randomized, placebo-controlled, multiple ascending-dose phase 1 trial. Nat Med 2021;27:1079–87. 34127852 10.1038/s41591-021-01391-w

[3] Proença C, Ribeiro D, Freitas M, Fernandes E. Flavonoids as potential agents in the management of type 2 diabetes through the modulation of α-amylase and α-glucosidase activity: a review. Crit Rev Food Sci Nutr 2021;1–71.DOI:10.1080/10408398.2020.1862755 33427491

[4] Kokil GR, Veedu RN, Ramm GA, et al. Type 2 diabetes mellitus: limitations of conventional therapies and intervention with nucleic acid-based therapeutics. Chem Rev 2015;115:4719–43. 25918949 10.1021/cr5002832

[5] Forouhi NG, Wareham NJ. Epidemiology of diabetes. Medicine 2014;42:698–702. 25568613 10.1016/j.mpmed.2014.09.007 PMC4282306

[6] Brás NF, Santos-Martins D, Fernandes PA, Ramos MJ. Mechanistic pathway on human α-glucosidase maltase-glucoamylase unveiled by QM/MM calculations. J Phys Chem B 2018;122:3889–99. 29548257 10.1021/acs.jpcb.8b01321

[7] Dhital S, Warren FJ, Butterworth PJ, et al. Mechanisms of starch digestion by α-amylase-structural basis for kinetic properties. Crit Rev Food Sci Nutr 2017;57:875–92. 25751598 10.1080/10408398.2014.922043

[8] Shahzad D, Saeed A, Larik FA, et al. Novel C-2 symmetric molecules as α-glucosidase and α-amylase inhibitors: design, synthesis, kinetic evaluation, molecular docking and pharmacokinetics. Molecules 2019;24:1511. 10.3390/molecules24081511 PMC6515238 30999646

[9] Elferink H, Bruekers JPJ, Veeneman GH, Boltje TJ. A comprehensive overview of substrate specificity of glycoside hydrolases and transporters in the small intestine: "a gut feeling". Cell Mol Life Sci 2020;77:4799–826. 32506169 10.1007/s00018-020-03564-1 PMC7658089

[10] Liu J. Oleanolic acid and ursolic acid: Research perspectives. J Ethnopharmacol 2005;100:92–4. 15994040 10.1016/j.jep.2005.05.024

[11] Sultana N, Ata A. Oleanolic acid and related derivatives as medicinally important compounds. J Enzyme Inhib Med Chem 2008;23:739–56. 18618318 10.1080/14756360701633187

[12] Pollier J, Goossens A. Oleanolic acid. Phytochemistry 2012;77:10–5. 22377690 10.1016/j.phytochem.2011.12.022

[13] Castellano JM, Guinda A, Delgado T, et al. Biochemical basis of the antidiabetic activity of oleanolic acid and related pentacyclic triterpenes. Diabetes 2013;62:1791–9. 23704520 10.2337/db12-1215 PMC3661625

[14] Loza-Rodríguez H, Estrada-Soto S, Alarcón-Aguilar FJ, et al. Oleanolic acid induces a dual agonist action on PPARγ/α and GLUT4 translocation: a pentacyclic triterpene for dyslipidemia and type 2 diabetes. Eur J Pharmacol 2020;883:173252. 32534078 10.1016/j.ejphar.2020.173252

[15] Meng YQ, Nie HH, Wang XC, et al. Synthesis and anti-tumor activity of oleanolic acid derivatives. Acta Pharm Sin 2011;46:1215–20. 22242453

[16] Bednarczyk-Cwynar B, Zaprutko L, Marciniak J, et al. The analgesic and anti-inflammatory effect of new oleanolic acid acyloxyimino derivative. Eur J Pharm Sci 2012;47:549–55. 22867936 10.1016/j.ejps.2012.07.017

[17] Ma CM, Nakamura N, Hattori M. Chemical modification of oleanene type triterpenes and their inhibitory activity against HIV-1 protease dimerization. Chem Pharm Bull 2000;48:1681–8. 10.1248/cpb.48.1681 11086896

[18] Chen J, Liu J, Zhang LY, et al. Pentacyclic triterpenes. Part 3: synthesis and biological evaluation of oleanolic acid derivatives as novel inhibitors of glycogen phosphorylase. Bioorg Med Chem Lett 2006;16:2915–9. 16546381 10.1016/j.bmcl.2006.03.009

[19] Bednarczyk-Cwynar B, Zaprutko L. Recent advances in synthesis and biological activity of triterpenic acylated oximes. Phytochem Rev 2015;14:203–31. 25859175 10.1007/s11101-014-9353-5 PMC4379416

[20] Zhong YY, Chen HS, Wu PP, et al. Synthesis and biological evaluation of novel oleanolic acid analogues as potential α-glucosidase inhibitors. Eur J Med Chem 2019;164:706–16. 30677669 10.1016/j.ejmech.2018.12.046

[21] Wu PP, He H, Ma H, et al. Oleanolic acid indole derivatives as novel *α*-glucosidase inhibitors: synthesis, biological evaluation, and mechanistic analysis. Bioorg Chem 2021;107:104580. 33418317 10.1016/j.bioorg.2020.104580

[22] Chen X, Gao M, Jian R, et al. Design, synthesis and α-glucosidase inhibition study of novel embelin derivatives. J Enzyme Inhib Med Chem 2020;35:565–73. 31969031 10.1080/14756366.2020.1715386 PMC7006637

[23] Chen P, Zhang Q, Dang H, et al. Screening for potential new probiotic based on probiotic properties and α-glucosidase inhibitory activity. Food Control 2014;35:65–72.

[24] Matsui T, Ueda T, Oki T, et al. Alpha-Glucosidase inhibitory action of natural acylated anthocyanins. 1. Survey of natural pigments with potent inhibitory activity. J Agric Food Chem 2001;49:1948–51. 11308351 10.1021/jf001251u

[25] Han L, Fang C, Zhu R, et al. Inhibitory effect of phloretin on α-glucosidase: kinetics, interaction mechanism and molecular docking. Int J Biol Macromol 2017;95:520–7. 27894824 10.1016/j.ijbiomac.2016.11.089

[26] Chi G, Wang L, Chen B, et al. Polyoxometalates: study of inhibitory kinetics and mechanism against α-glucosidase. J Inorg Biochem 2019;199:110784. 31351380 10.1016/j.jinorgbio.2019.110784

[27] Taha M, Imran S, Ismail NH, et al. Biology-oriented drug synthesis (BIODS) of 2-(2-methyl-5-nitro-1H-imidazol-1-yl)ethyl aryl ether derivatives, *in vitro* α-amylase inhibitory activity and *in silico* studies. Bioorg Chem 2017;74:1–9. 28719801 10.1016/j.bioorg.2017.07.001

[28] Taha M, Javid MT, Imran S, et al. Synthesis and study of the α-amylase inhibitory potential of thiadiazole quinoline derivatives. Bioorg Chem 2017;74:179–86. 28826047 10.1016/j.bioorg.2017.08.003

[29] Hameed S, Kanwal F, Seraj R, et al. Synthesis of benzotriazoles derivatives and their dual potential as α-amylase and α-glucosidase inhibitors *in vitro*: Structure-activity relationship, molecular docking, and kinetic studies. Eur J Med Chem 2019;183:111677. 31514061 10.1016/j.ejmech.2019.111677

[30] Wang GC, Wang J, Xie ZZ, et al. Discovery of 3,3-di(indolyl)indolin-2-one as a novel scaffold for α-glucosidase inhibitors: in silico studies and SAR predictions. Bioorg Chem 2017;72:228–33. 28482263 10.1016/j.bioorg.2017.05.006

[31] Lin P, Zeng JC, Chen JG, et al. Z.P. Yin, Synthesis, in vitro inhibitory activity, kinetic study and molecular docking of novel N-alkyl-deoxynojirimycin derivatives as potential α-glucosidase inhibitors. J Enzym Inhib Med Ch 2020;35:1879–90. 10.1080/14756366.2020.1826941 PMC7580737 33003963

[32] Floris S, Fais A, Medda R, et al. Washingtonia filifera seed extracts inhibit the islet amyloid polypeptide fibrils formations and α-amylase and α-glucosidase activity. J Enzyme Inhib Med Chem 2021;36:517–24. 33494628 10.1080/14756366.2021.1874945 PMC7850368

[33] Wang GC, Peng ZY, Wang J, et al. Synthesis, in vitro evaluation and molecular docking studies of novel triazine-triazole derivatives as potential α-glucosidase inhibitors. Eur J Med Chem 2017;125:423–9. 27689725 10.1016/j.ejmech.2016.09.067

[34] Ali F, Khan KM, Salar U, et al. Hydrazinyl arylthiazole based pyridine scaffolds: Synthesis, structural characterization, *in vitro* α-glucosidase inhibitory activity, and in silico studies. Eur J Med Chem 2017;138:255–72. 28672278 10.1016/j.ejmech.2017.06.041

[35] Saleem F Kanwal KM, Khan S, Chigurupati , et al. Synthesis of azachalcones, their α-amylase, α-glucosidase inhibitory activities, kinetics, and molecular docking studies. Bioorg. Chem 2020;106:104489. 33272713 10.1016/j.bioorg.2020.104489

[36] Liu D, He WG, Wang ZH, et al. Design, synthesis and biological evaluation of 3'-benzylated analogs of 3'-epi-neoponkoranol as potent α-glucosidase inhibitors. Eur J Med Chem 2016;110:224–36. 26840363 10.1016/j.ejmech.2016.01.029

[37] Islam MS, Barakat A, Al-Majid AM, et al. A concise synthesis and evaluation of new malonamide derivatives as potential α-glucosidase inhibitors. Bioorg Med Chem 2016;24:1675–82. 26972921 10.1016/j.bmc.2016.02.037

