# Supplementary material for: Synthesis and bioactivities evaluation of oleanolic acid oxime ester derivatives as α-glucosidase and α-amylase inhibitors
Source: J Enzyme Inhib Med Chem. 2022 Jan 10;37(1):451–61. doi: 10.1080/14756366.2021.2018682 (PMC8757604; doi:10.1080/14756366.2021.2018682)
Supplement: Supplemental Material [file IENZ_A_2018682_SM6712.pdf]

Supporting Information

Synthesis and bioactivities evaluation of oleanolic acid oxime ester derivatives as α-glucosidase and α-amylase inhibitors

Xu-Yang Deng[a], Jun-Jie Ke [a], Ying-Ying Zheng [a], Dong-Li Li [a], Kun Zhang [a], Xi Zheng [b], Zhuang Xiong [a,*], Pan-Pan Wu [a,*], Xue-Tao Xu [a,*]

[a]*School of biotechnology and health sciences, Wuyi University, Jiangmen 529020, P.R. China*

[b]*Department of Chemical Biology, Ernest Mario School of Pharmacy, Rutgers, The State University of New Jersey, Piscataway, NJ 08854, USA*

1. NMR of compounds……………………………………………………...….2-21
2. ESI-MS of compounds…………………………………………………...…22-31

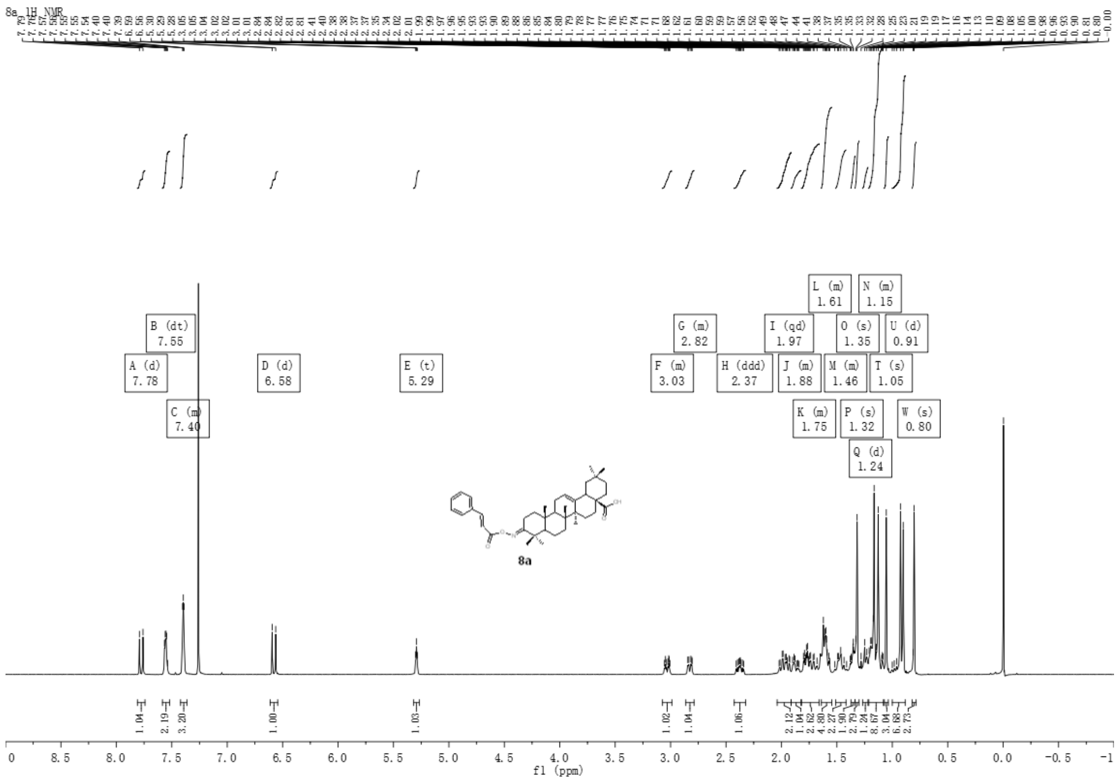

Fig.1 **3a** (^{1}H NMR)

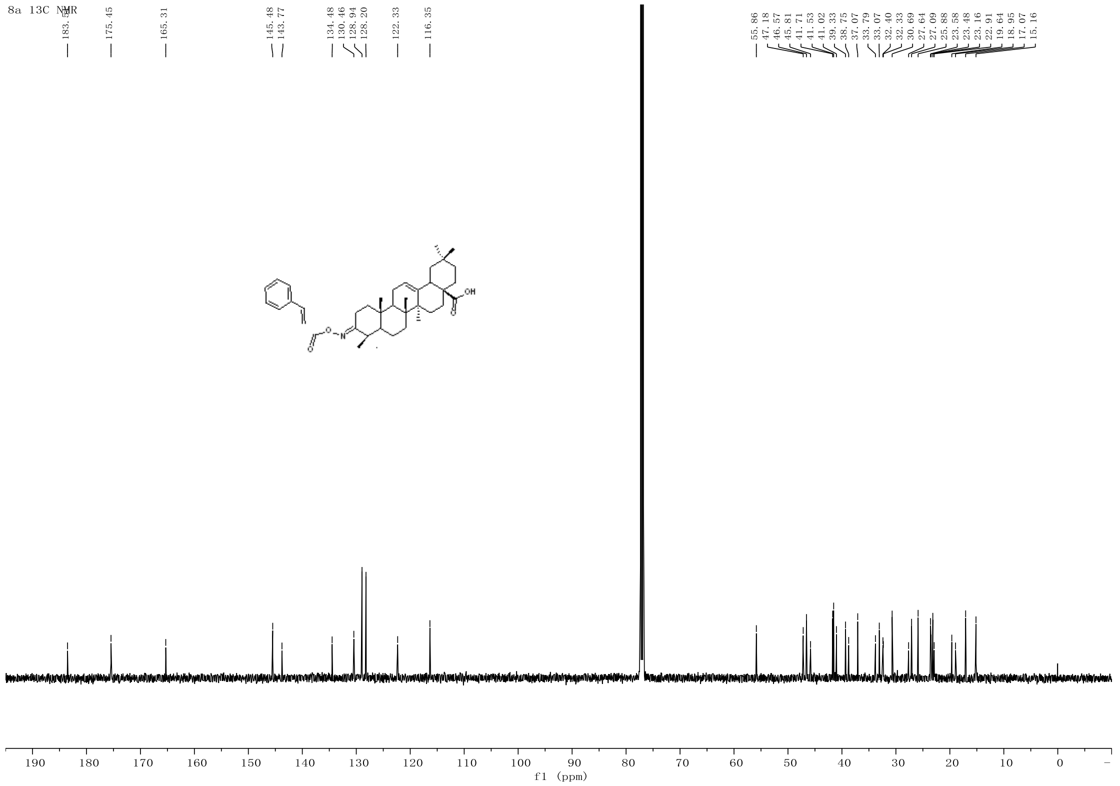

Fig.2 **3a** (^{13}C NMR)

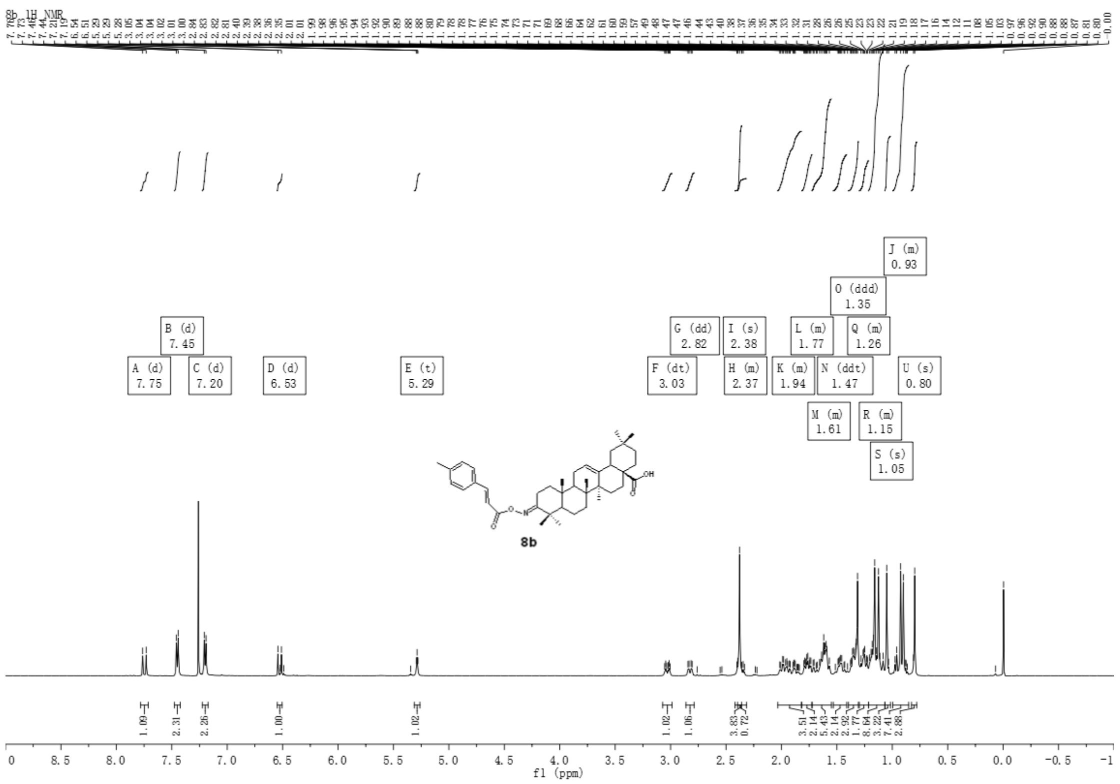

Fig.3 **3b (**^{1}H NMR)

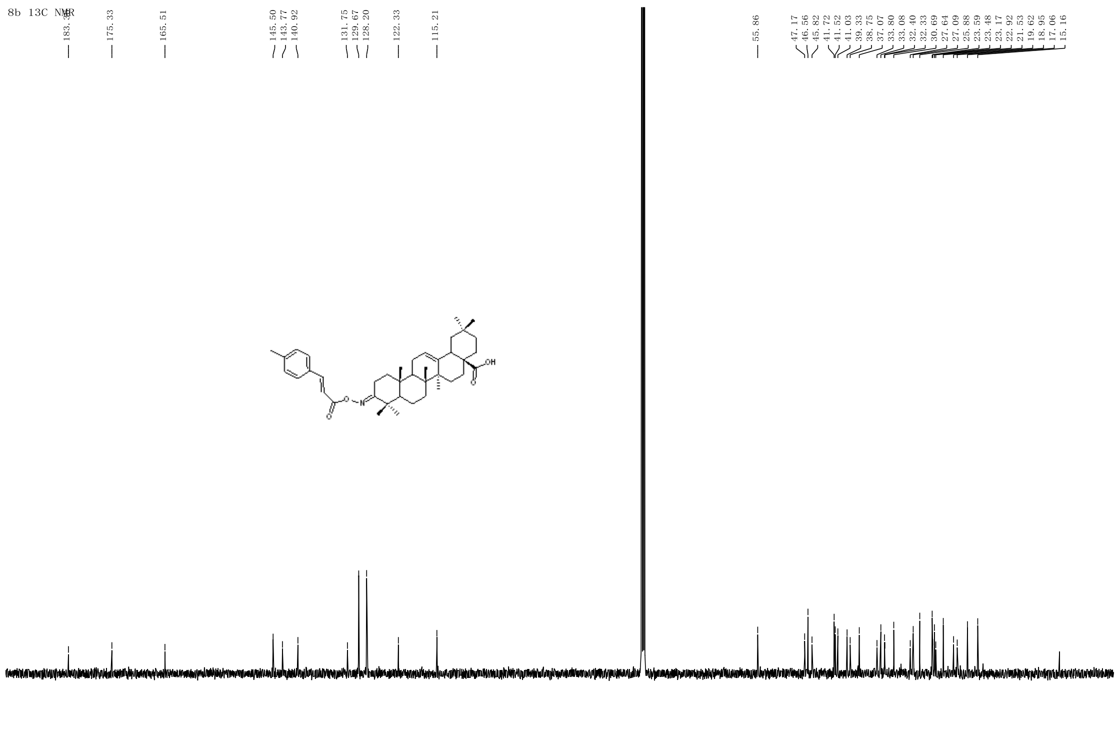

Fig.4 **3b (**^{13}C NMR)

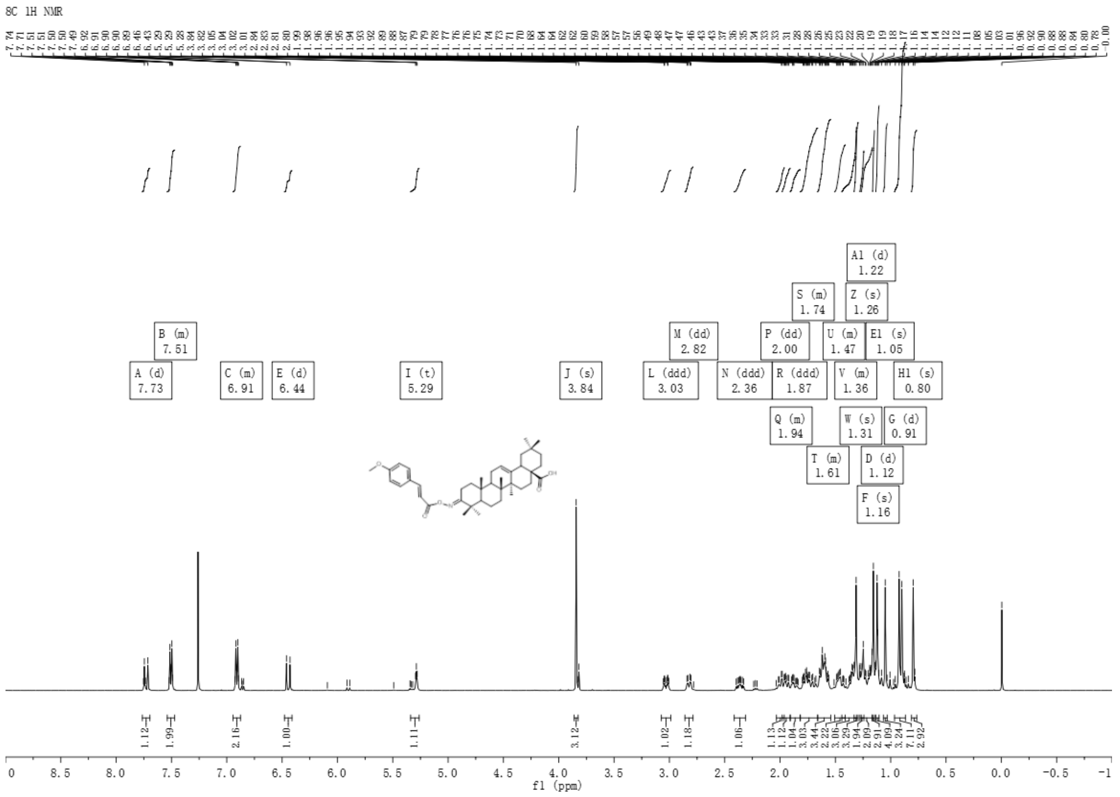

Fig.5 **3c (**^{1}H NMR)

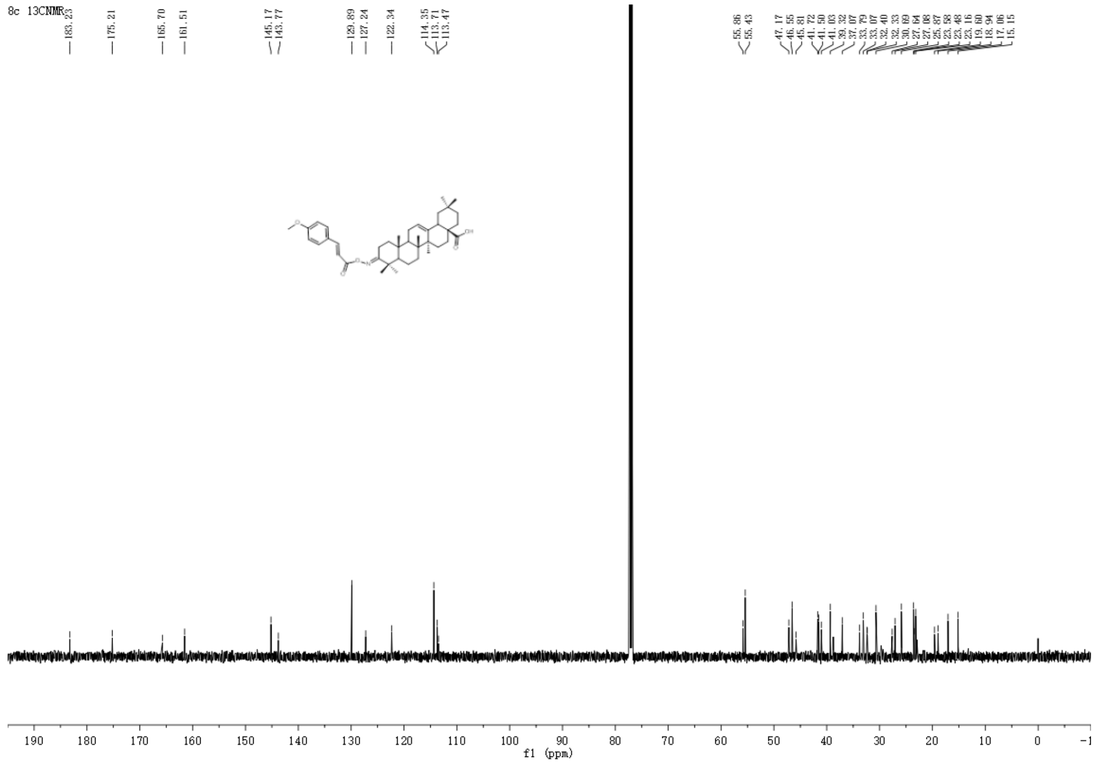

Fig.6 **3c (**^{13}C NMR)

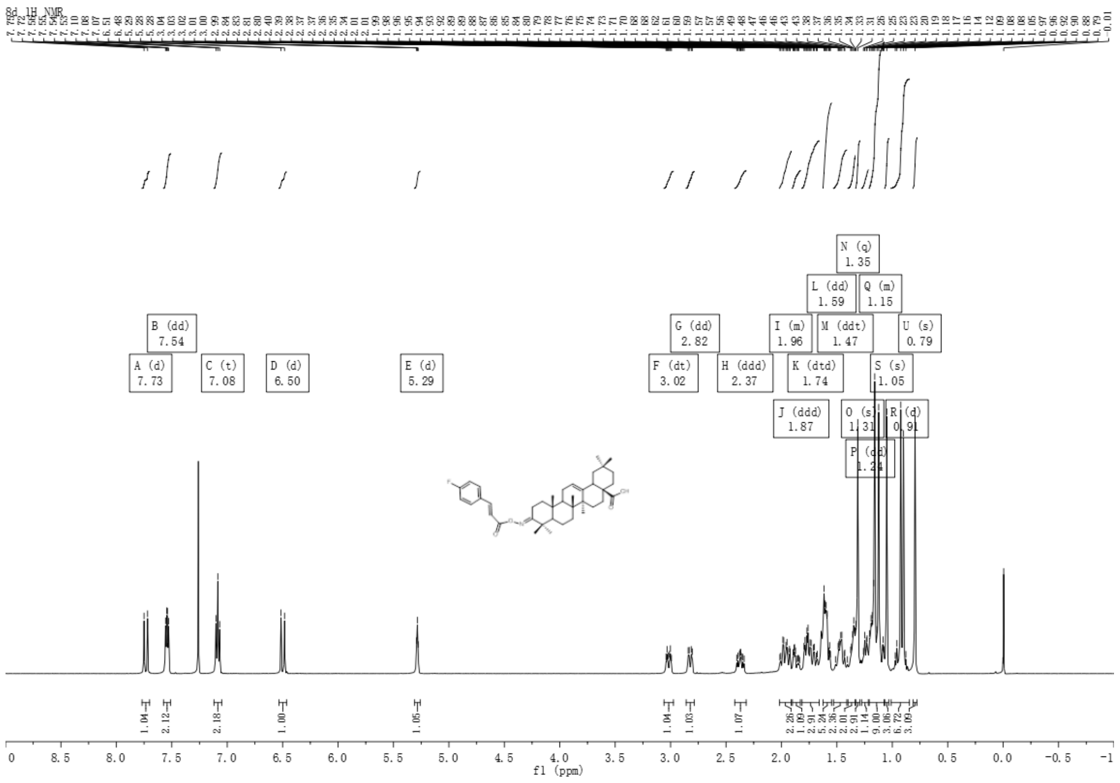

Fig.7 **3d (**^{1}H NMR)

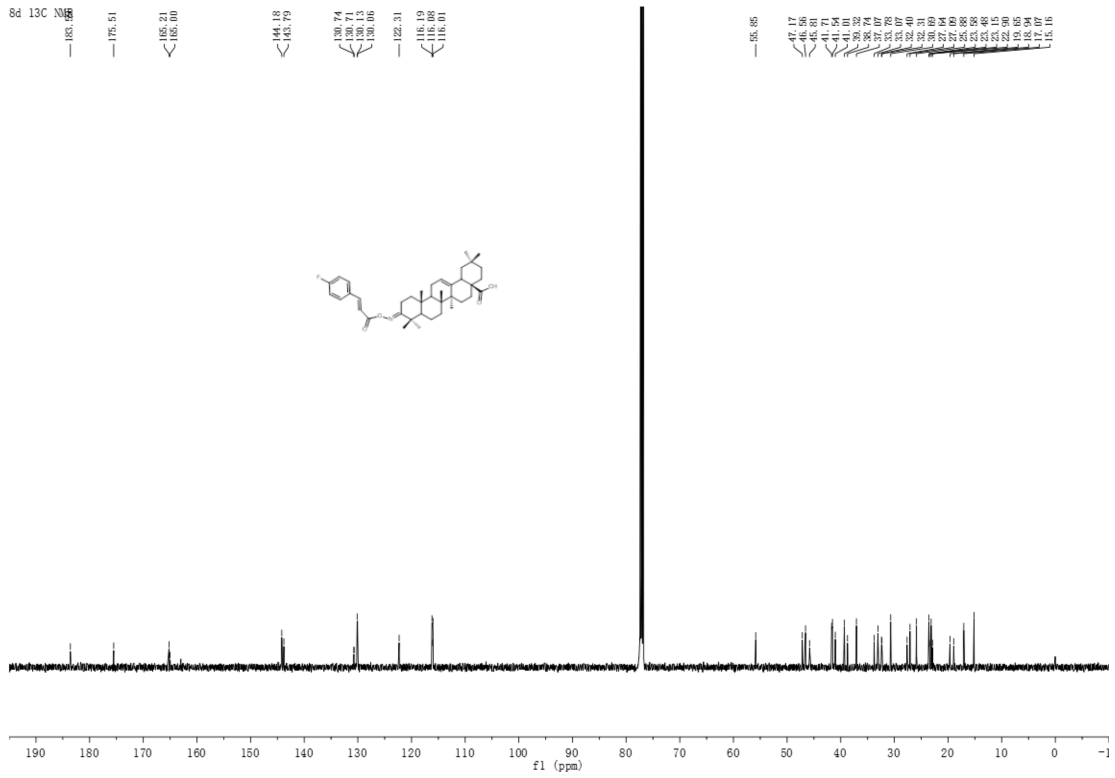

Fig.8 **3d (**^{13}C NMR)

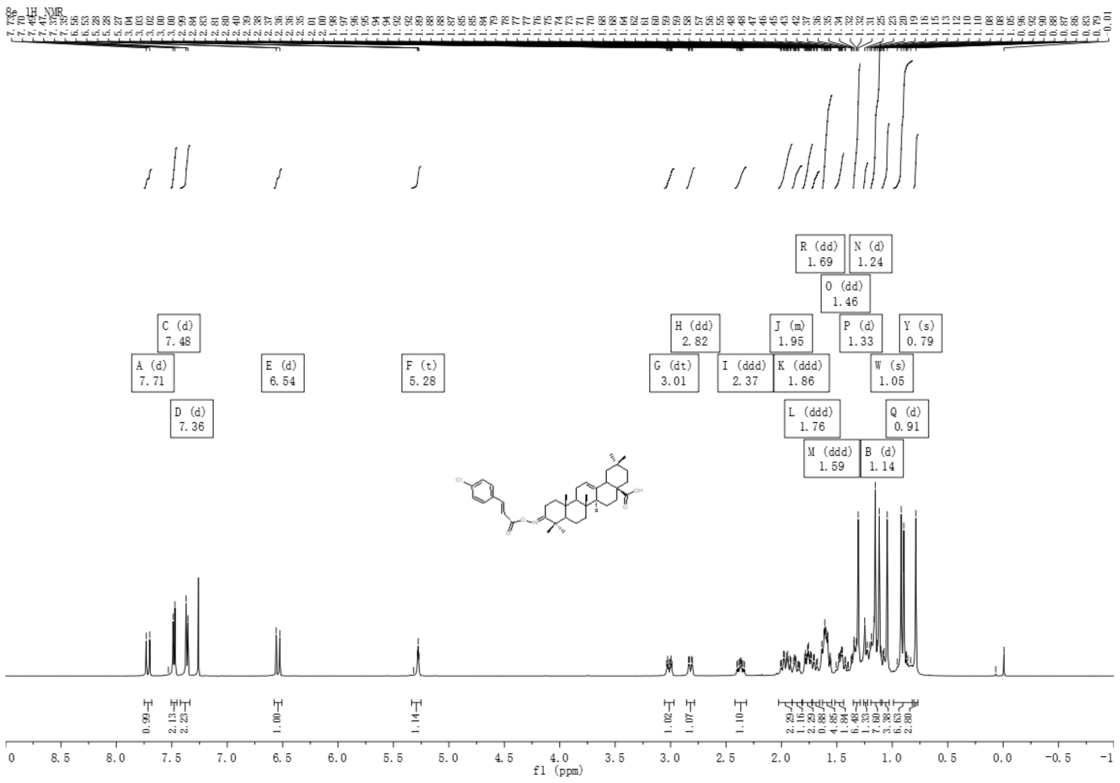

Fig.9 **3e (**^{1}H NMR)

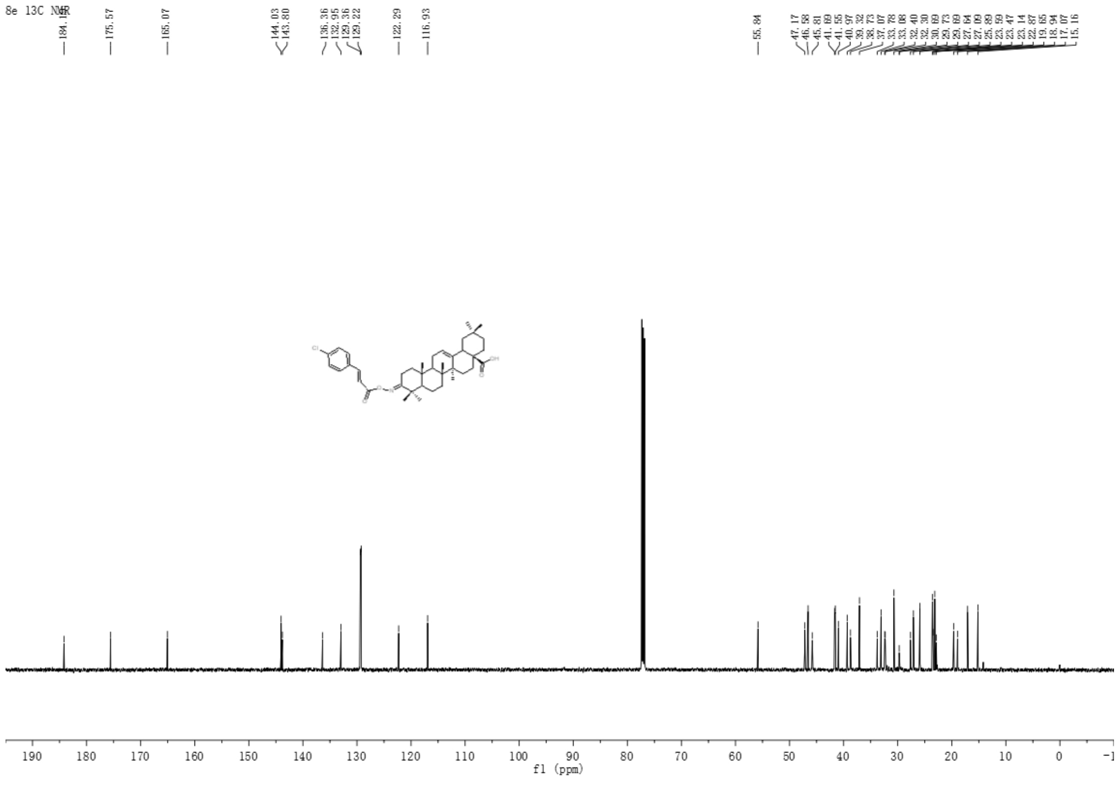

Fig.10 **3e (**^{13}C NMR)

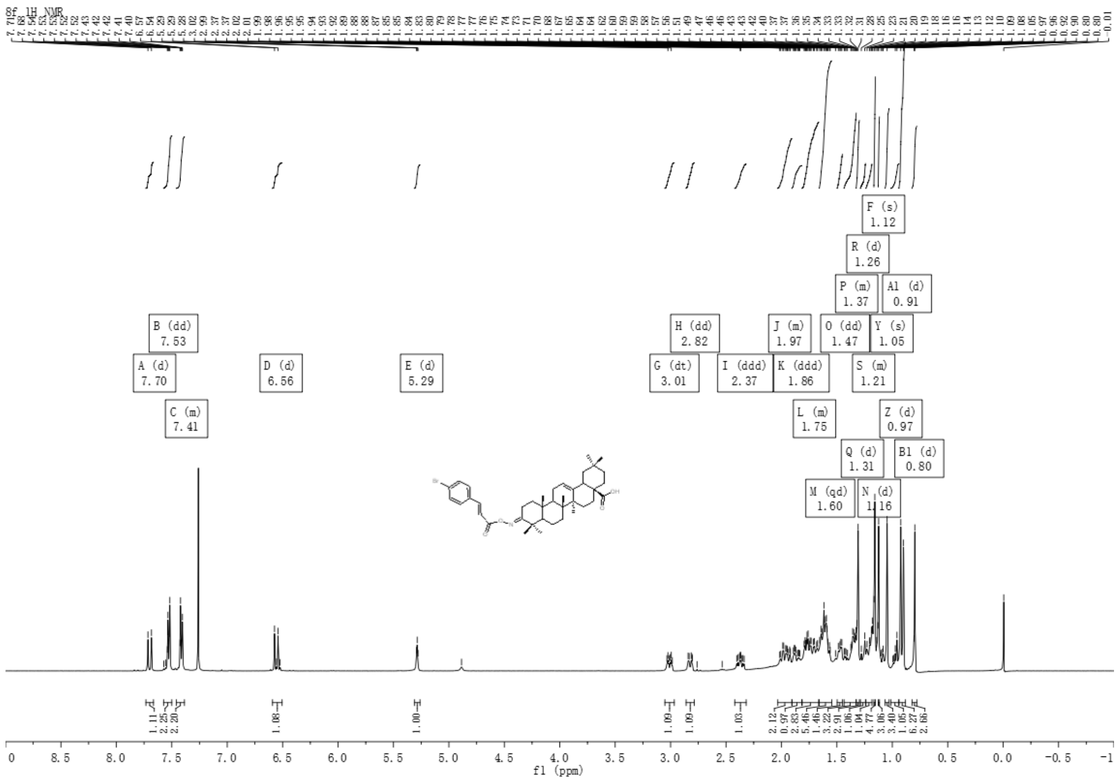

Fig.11 **3f (**^{1}H NMR)

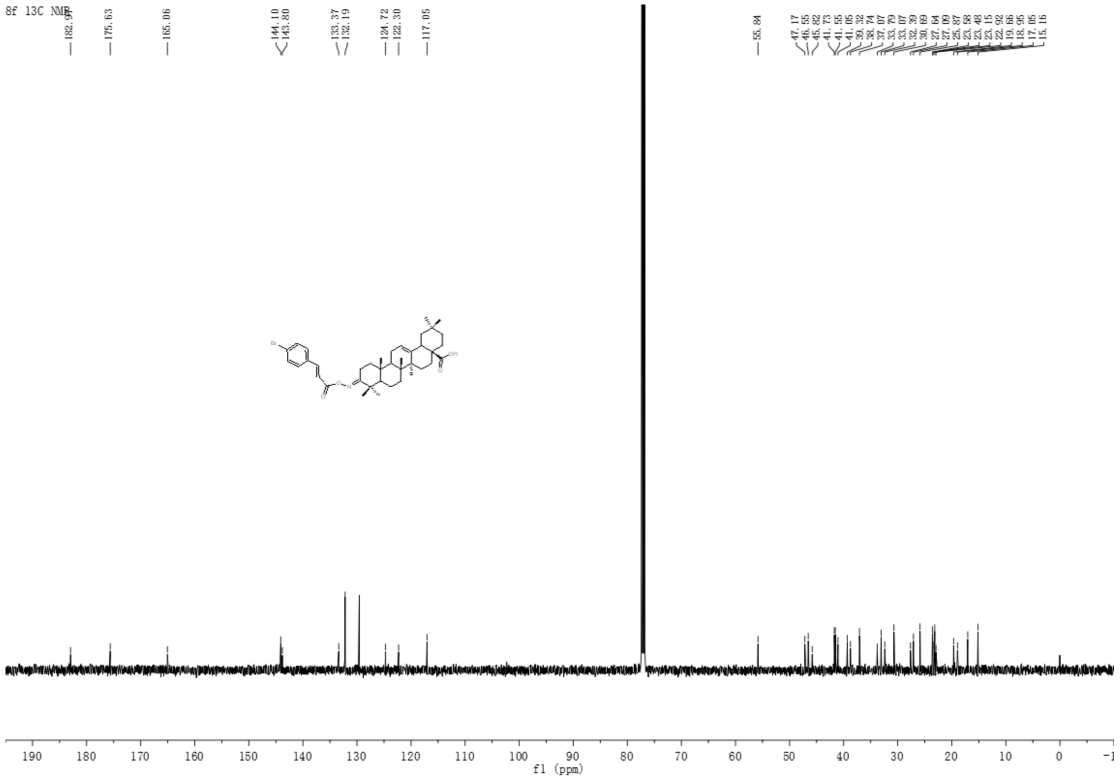

Fig.12 **3f (**^{13}C NMR)

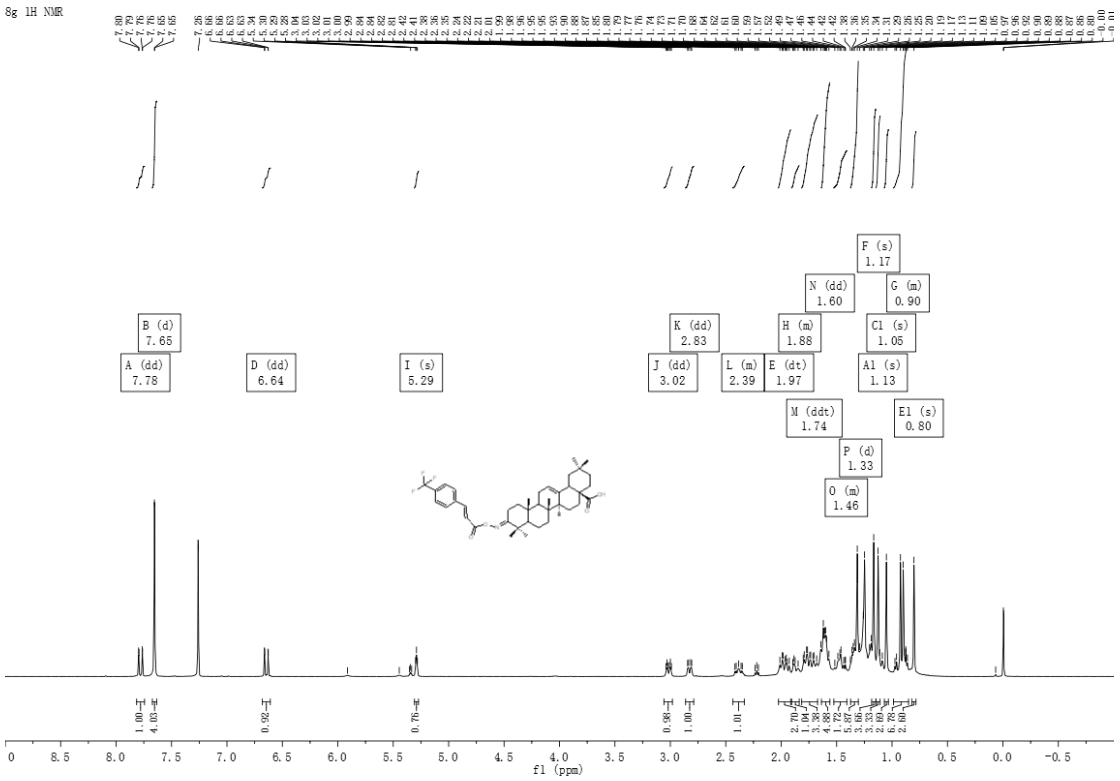

Fig.13 **3g** (^{1}H NMR)

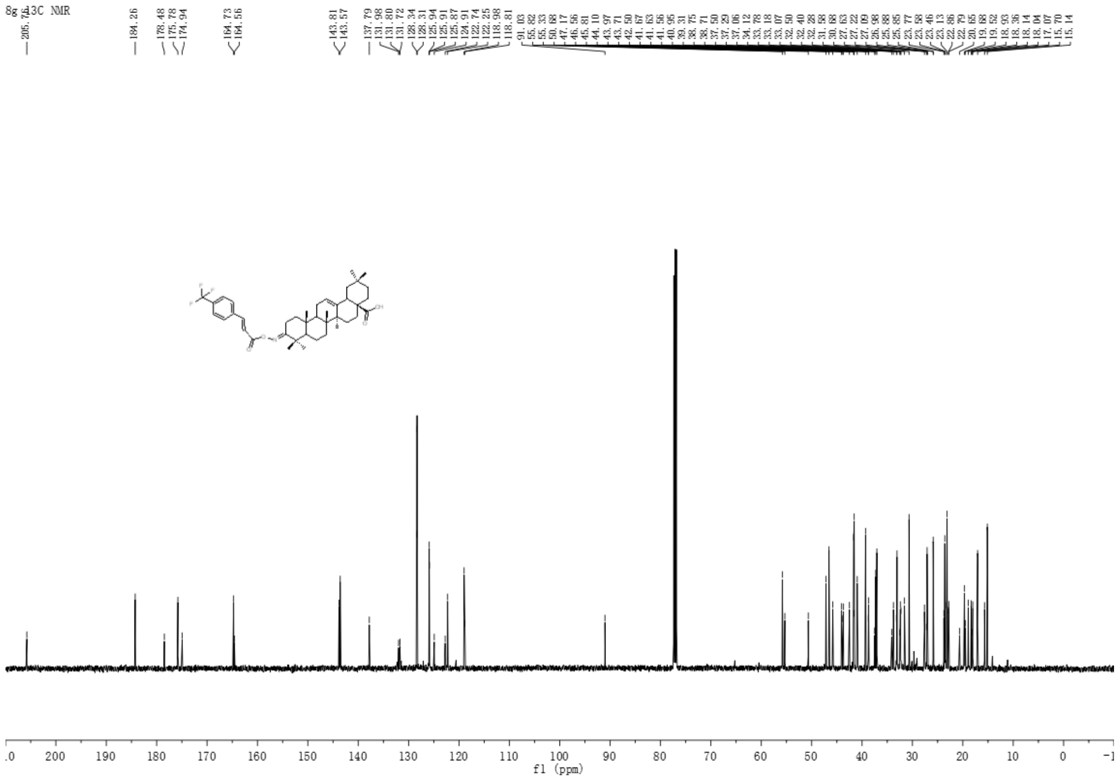

Fig.14 **3g** (^{13}C NMR)

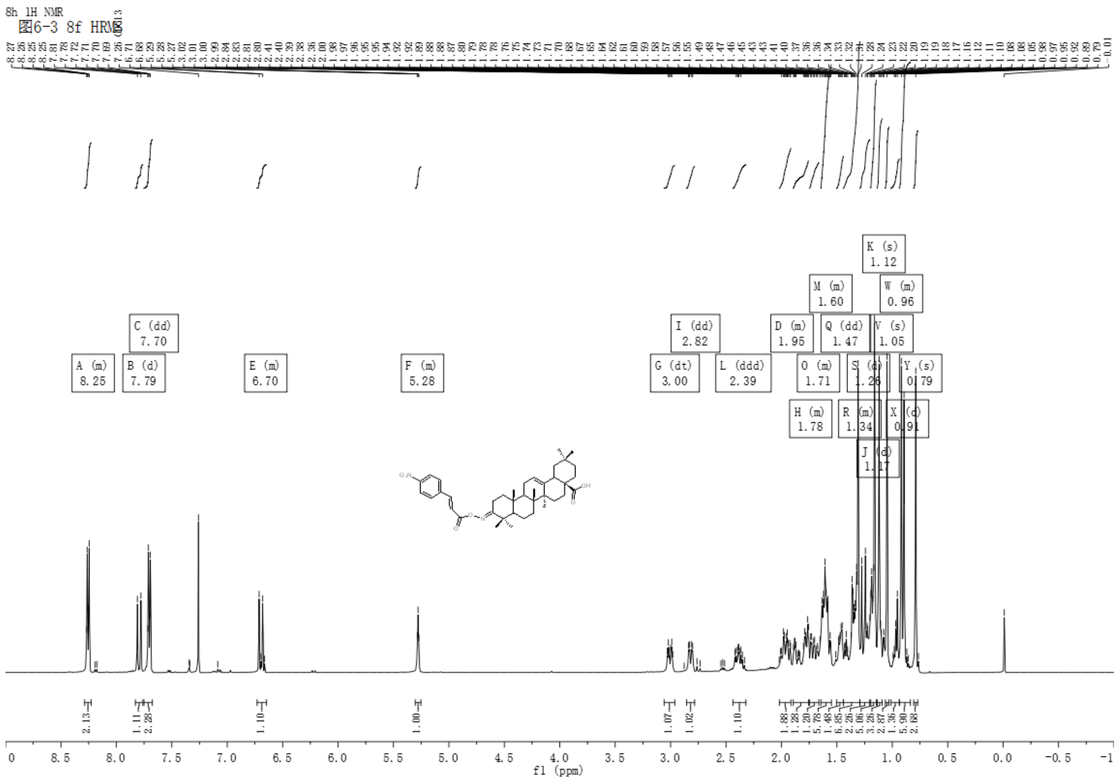

Fig.15 **3h** (^{1}H NMR)

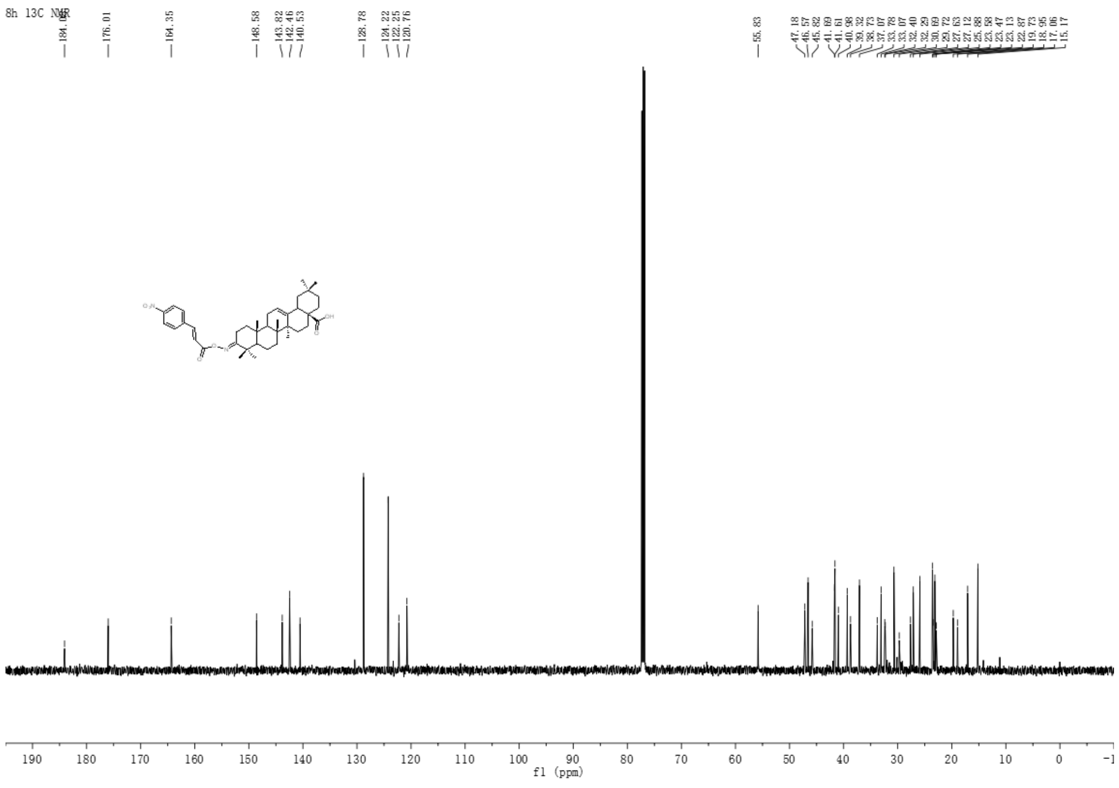

Fig.16 **3h** (^{13}C NMR)

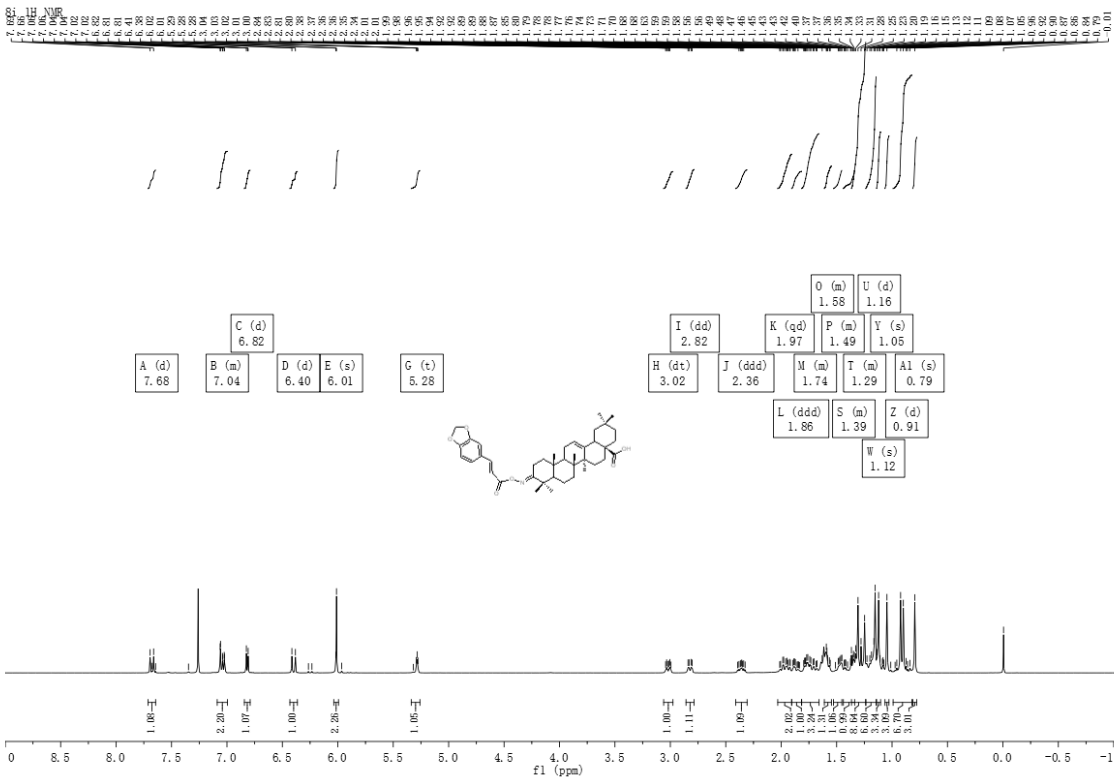

Fig.17 **3i (**^{1}H NMR)

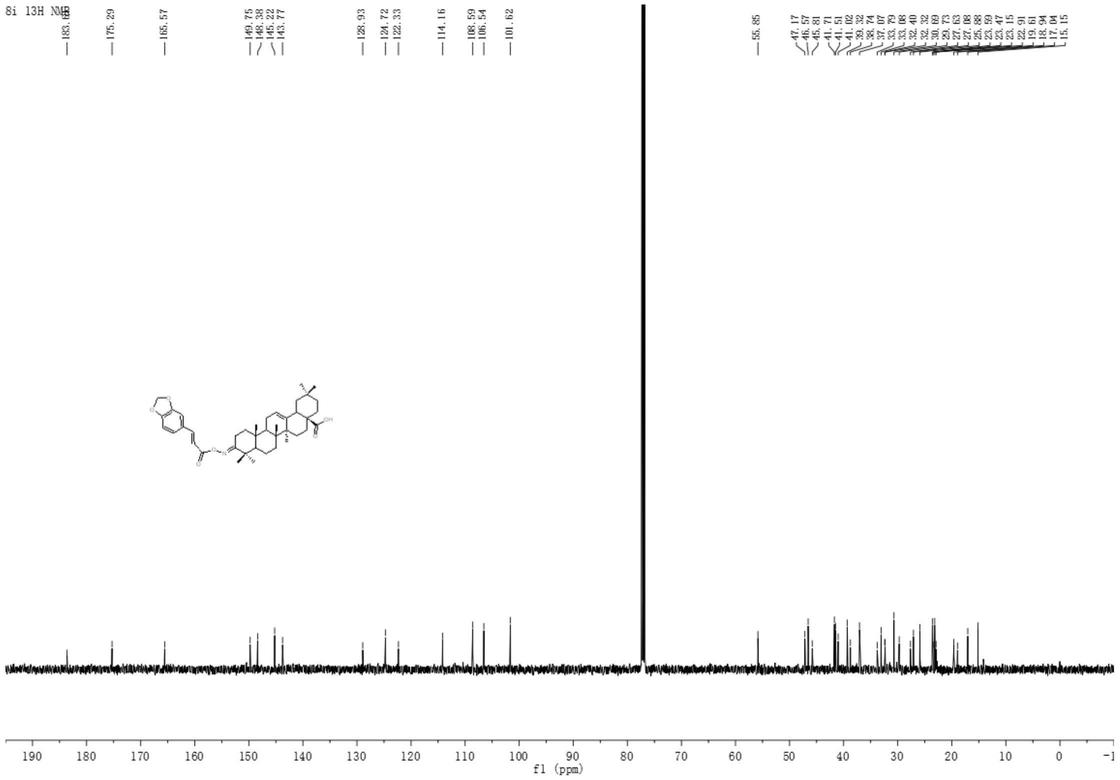

Fig.18 **3i (**^{13}C NMR)

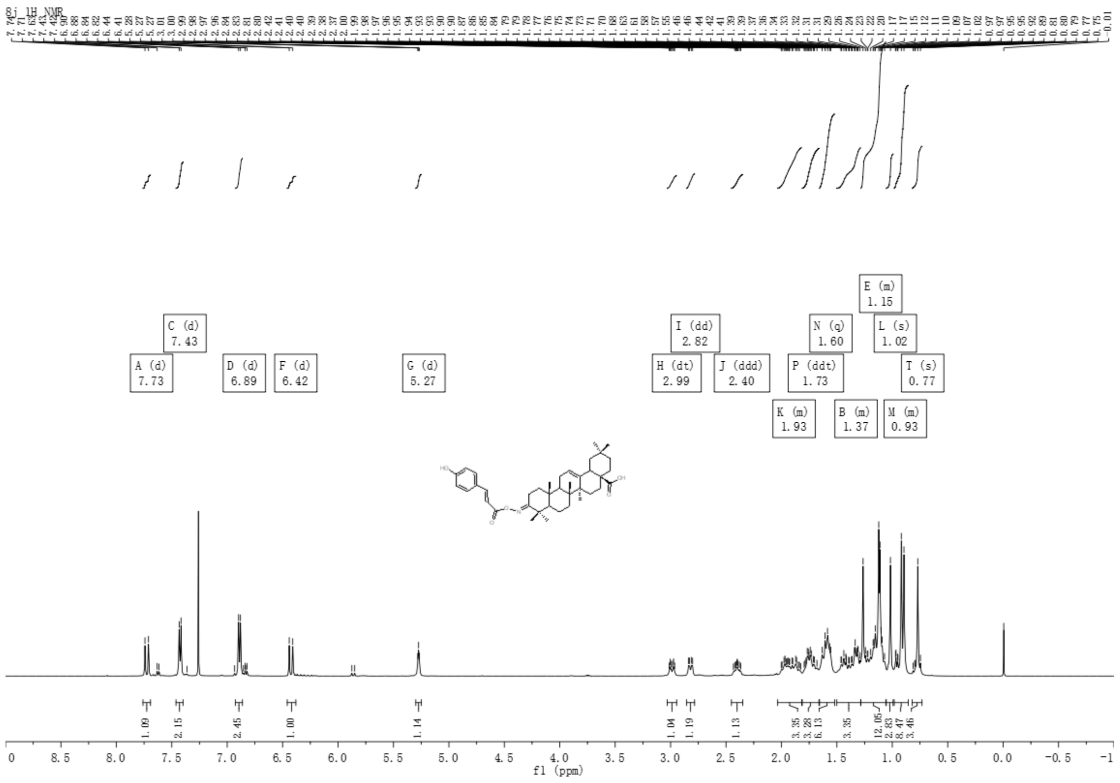

Fig.19 **3j** (^{1}H NMR)

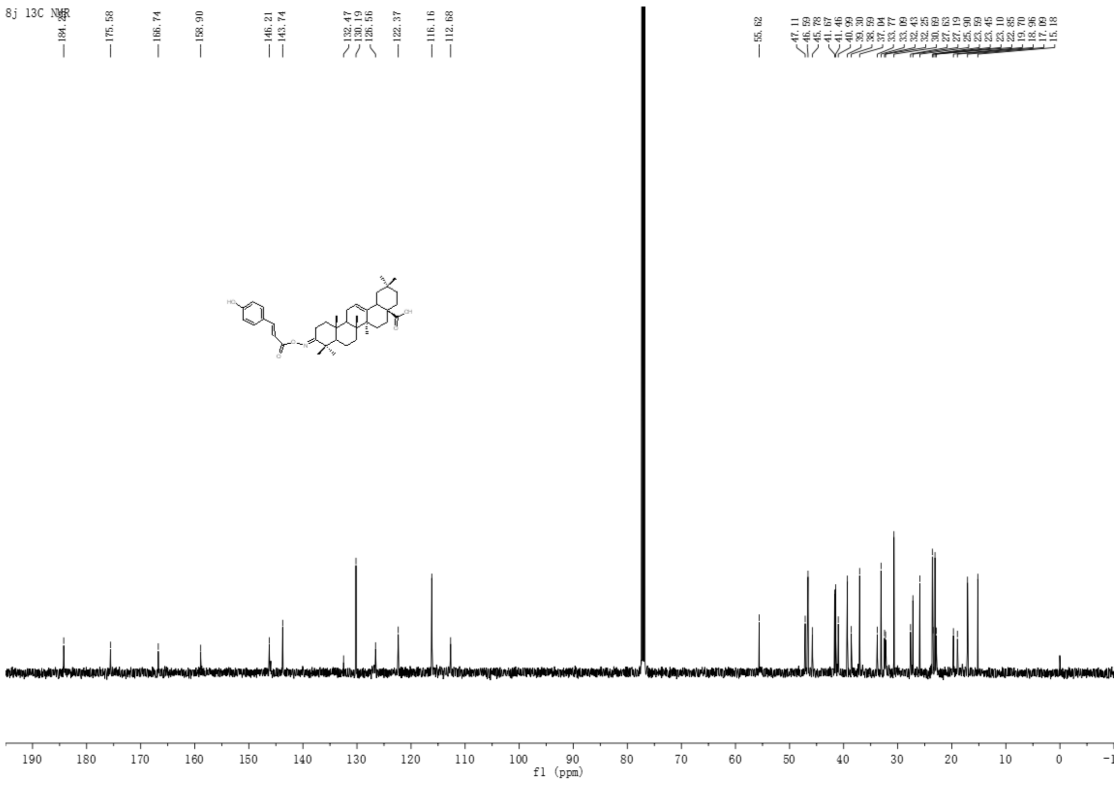

Fig.20 **3j** (^{13}C NMR)

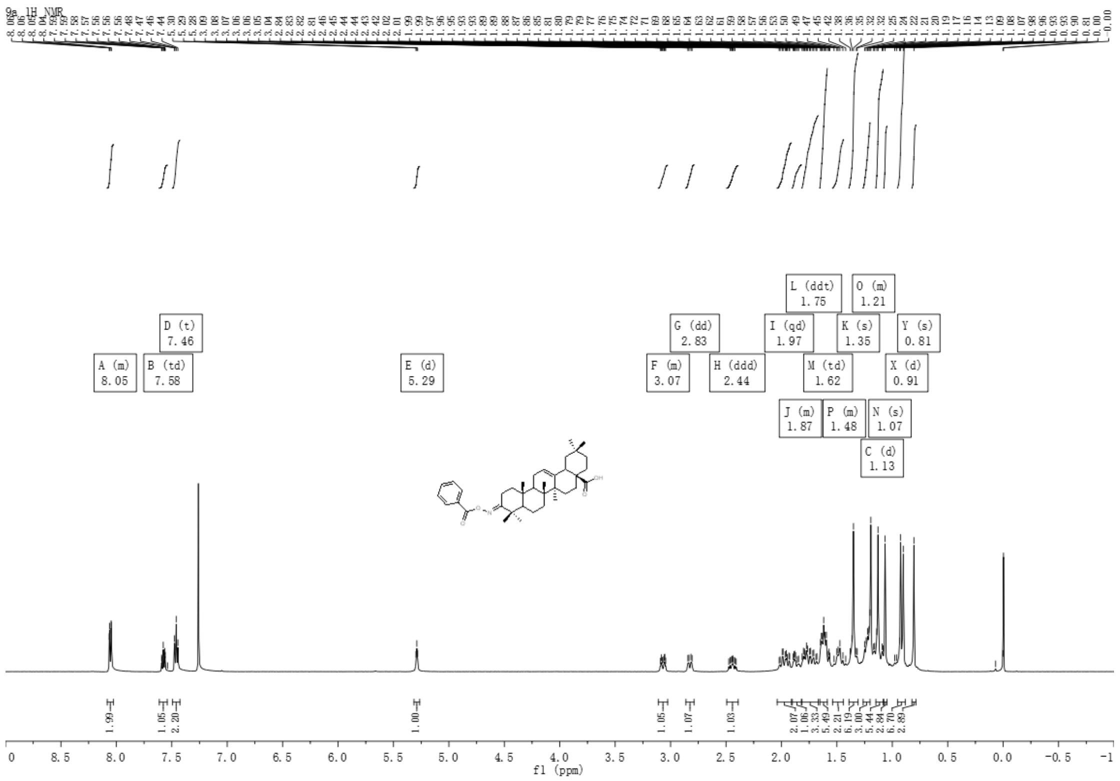

Fig.21 **3k** (^{1}H NMR)

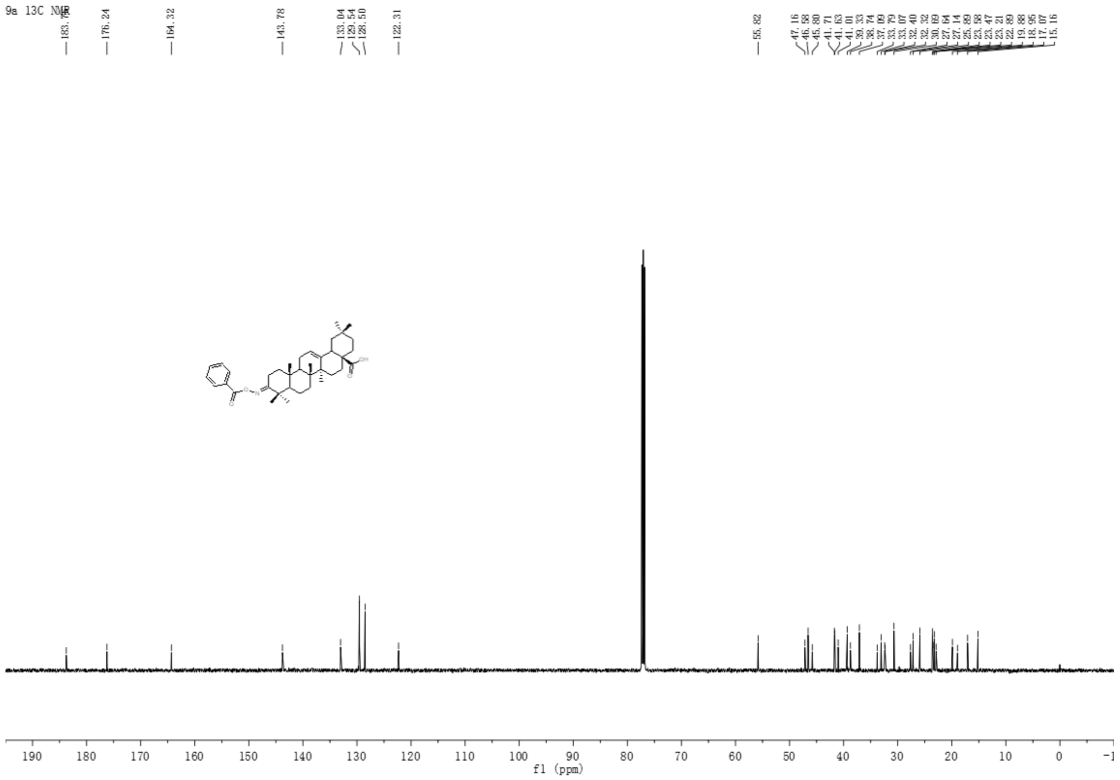

Fig.22 **3k** (^{13}C NMR)

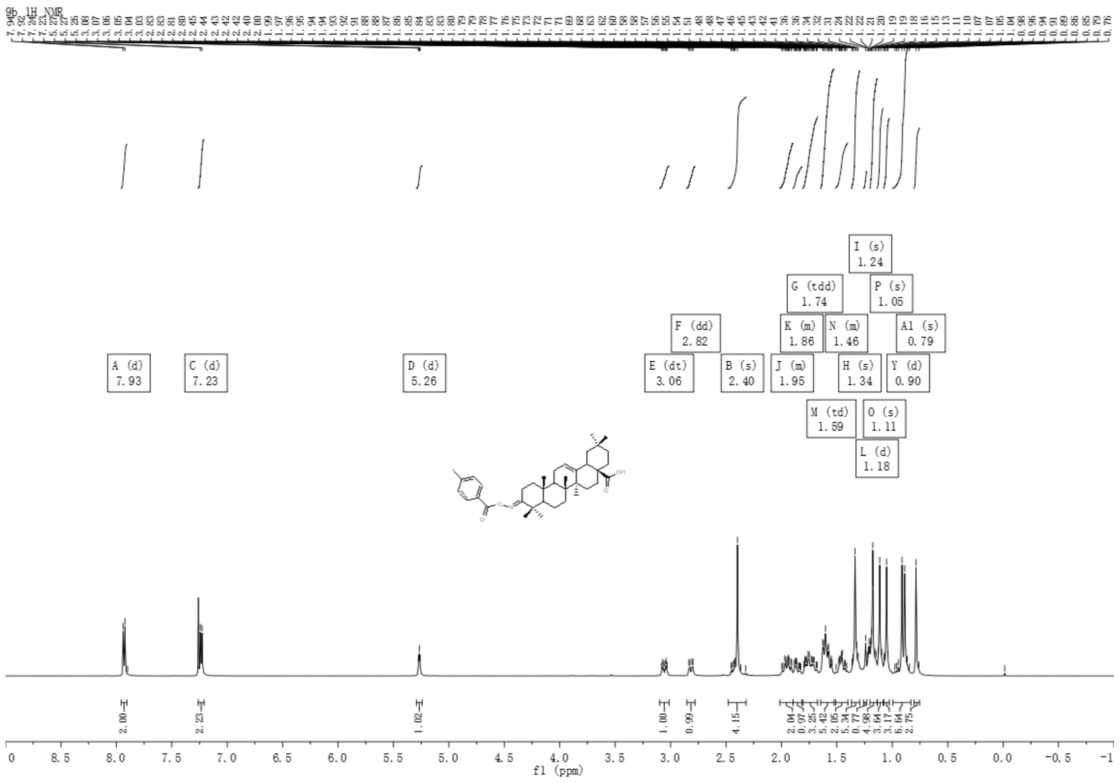

Fig.23 **3l (**^{1}H NMR)

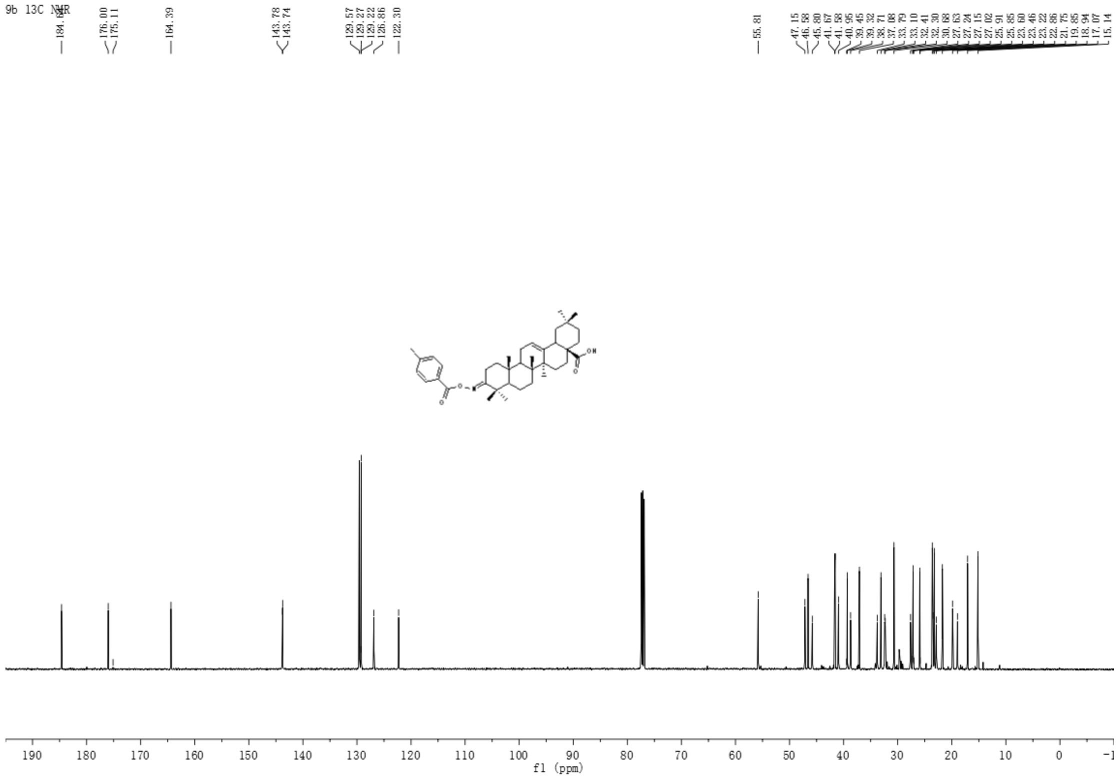

Fig.24 **3l (**^{13}C NMR)

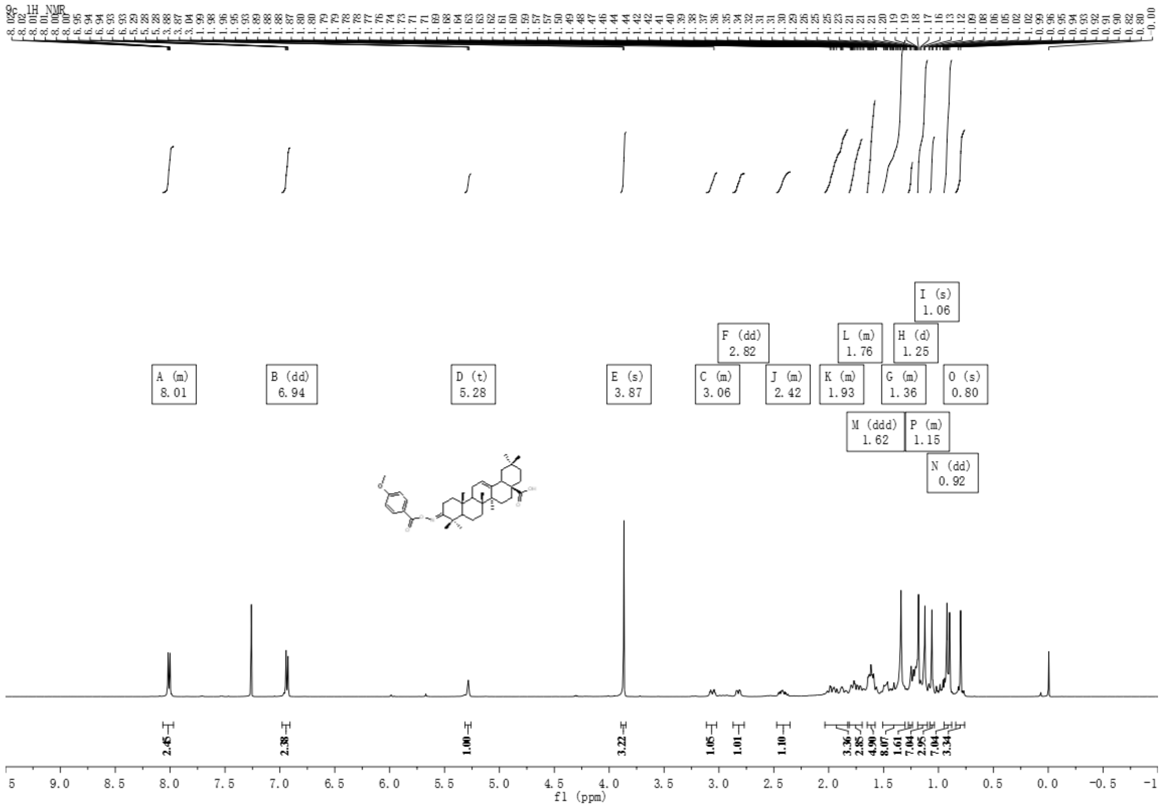

Fig.25 **3m (**^{1}H NMR)

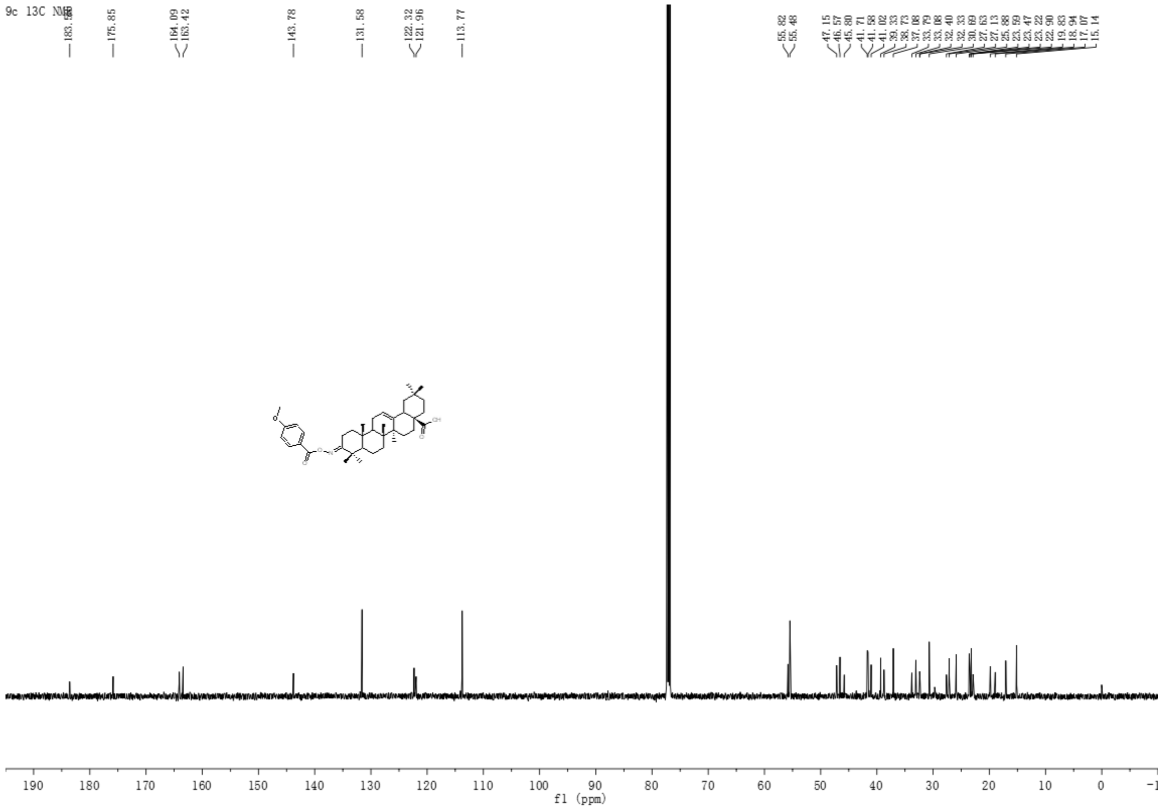

Fig.26 **3m (**^{13}C NMR)

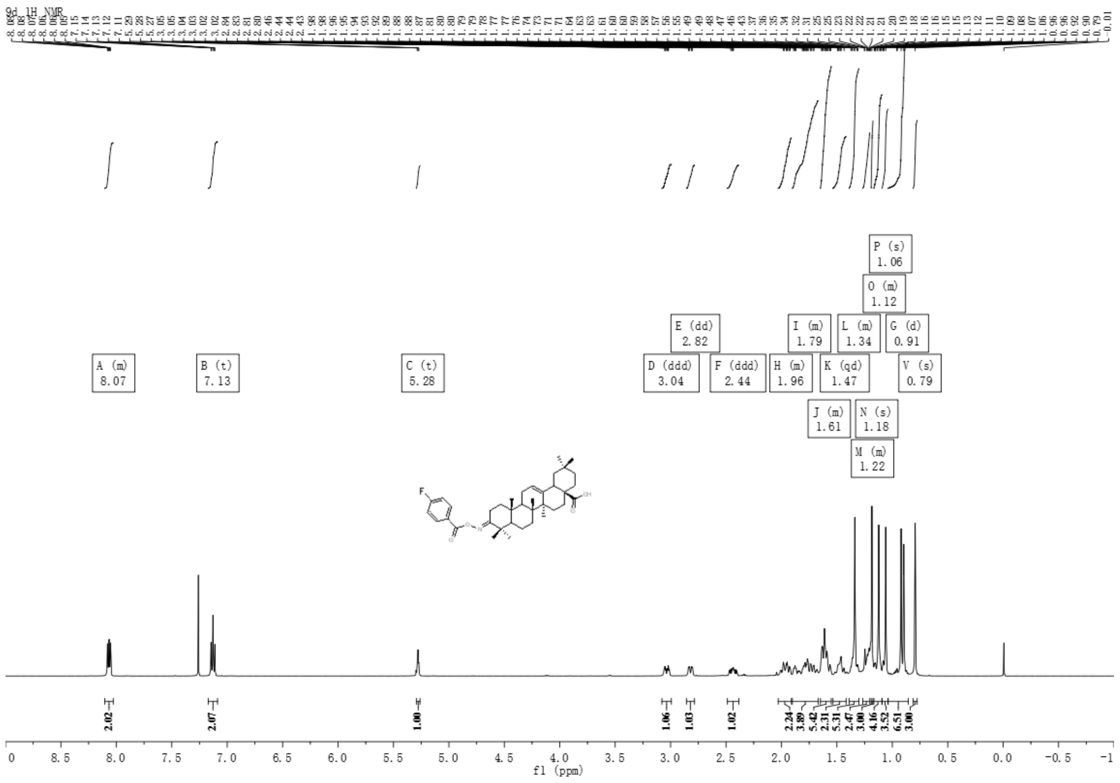

Fig.27 **3n** (^{1}H NMR)

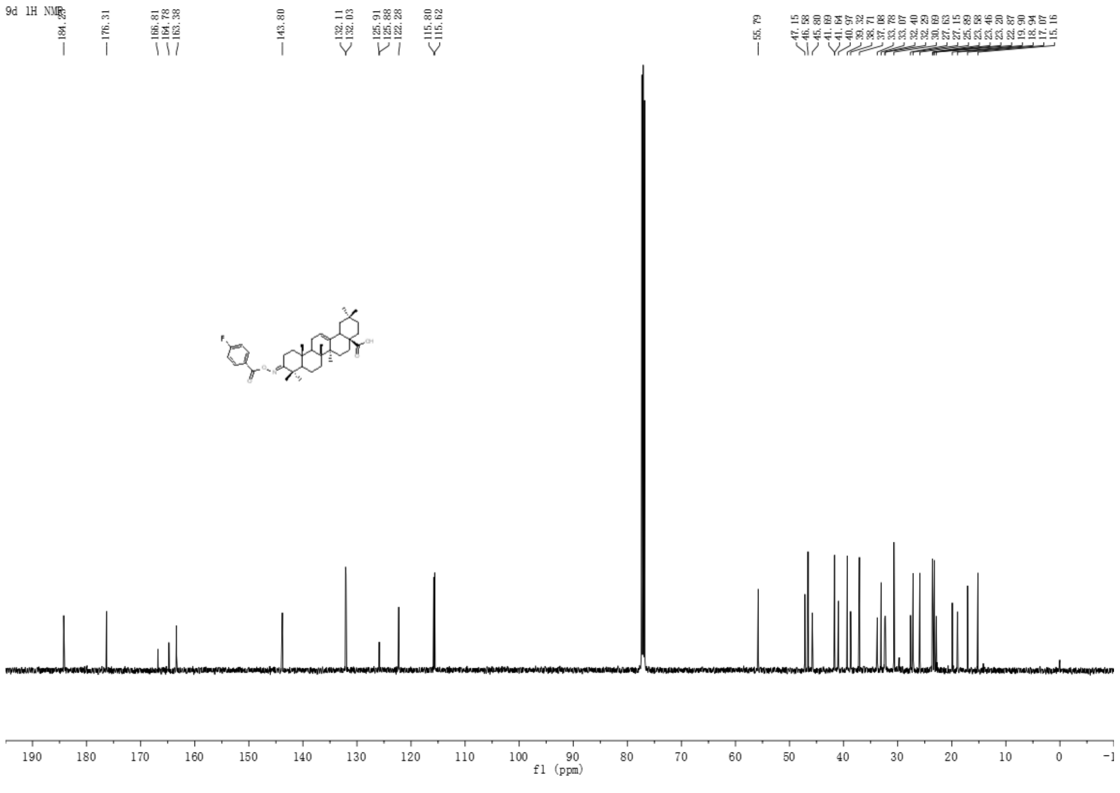

Fig.28 **3n** (^{13}C NMR)

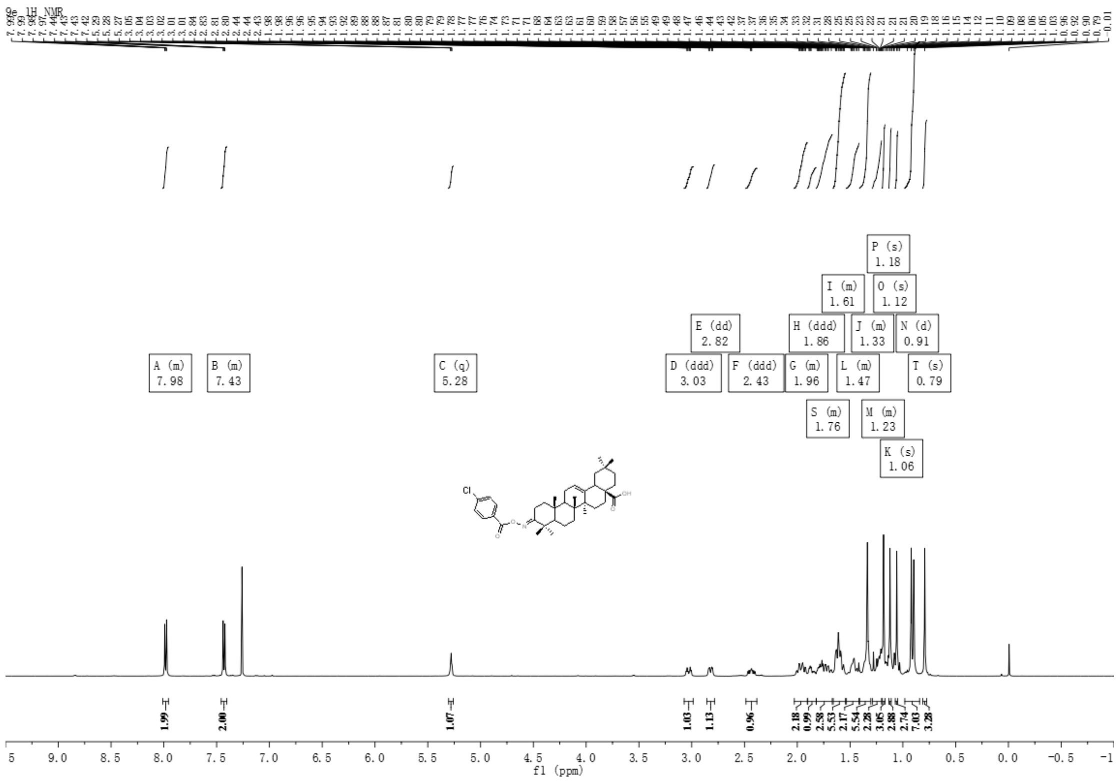

Fig.29 **3o** (^{1}H NMR)

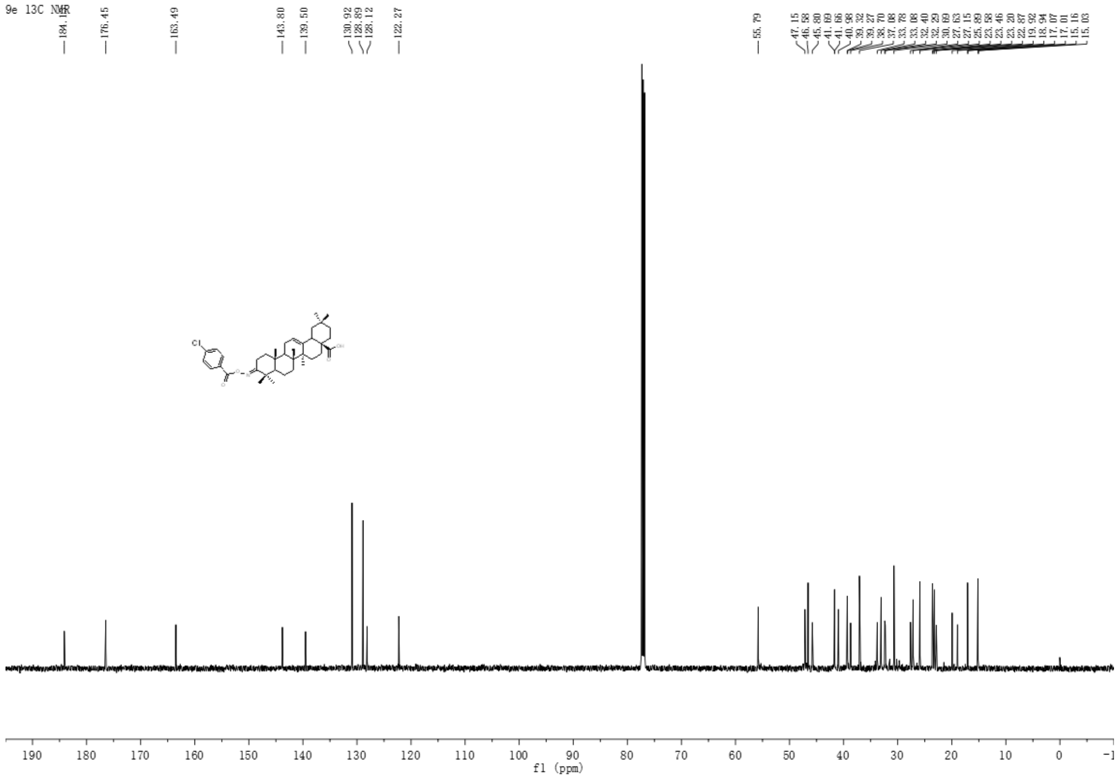

Fig.30 **3o** (^{13}C NMR)

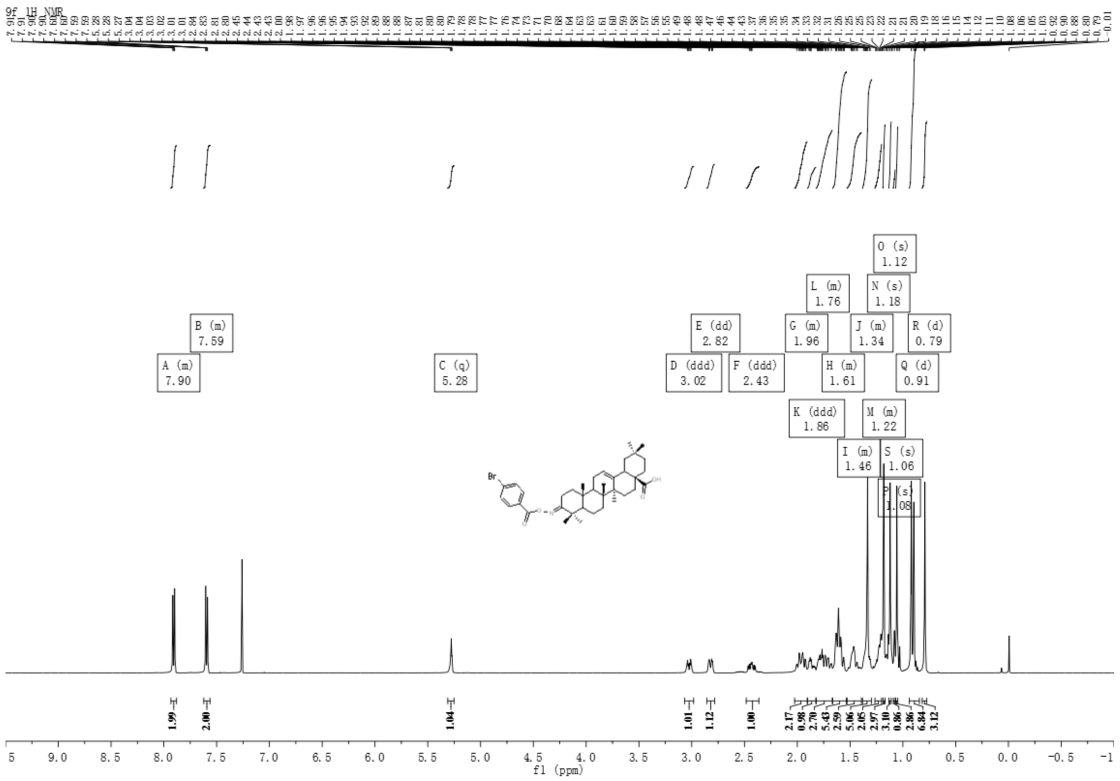

Fig.31 **3p (**^{1}H NMR)

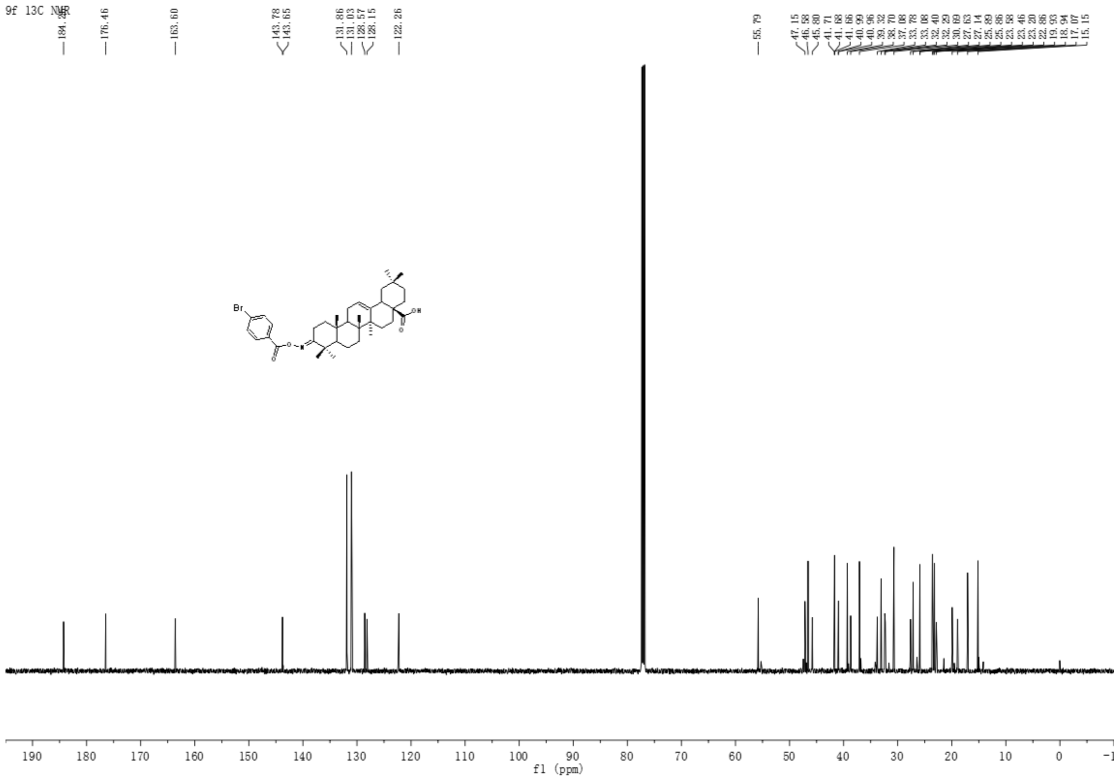

Fig.32 **3p (**^{13}C NMR)

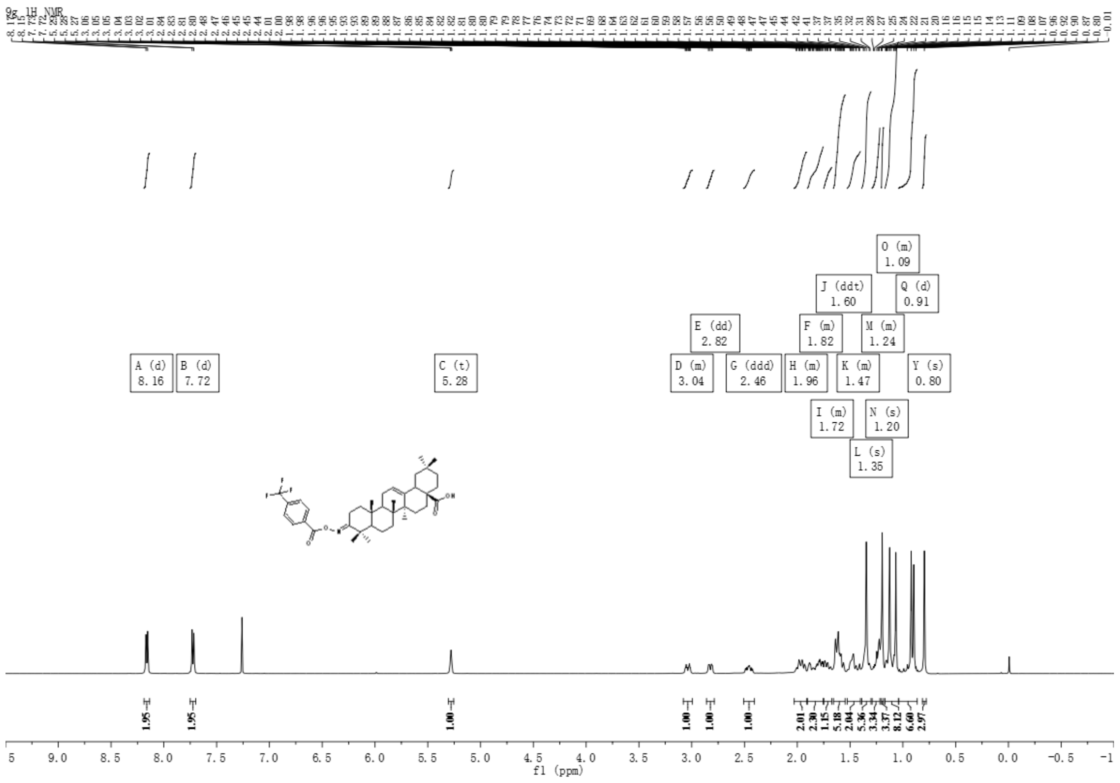

Fig.33 **3q** (^{1}H NMR)

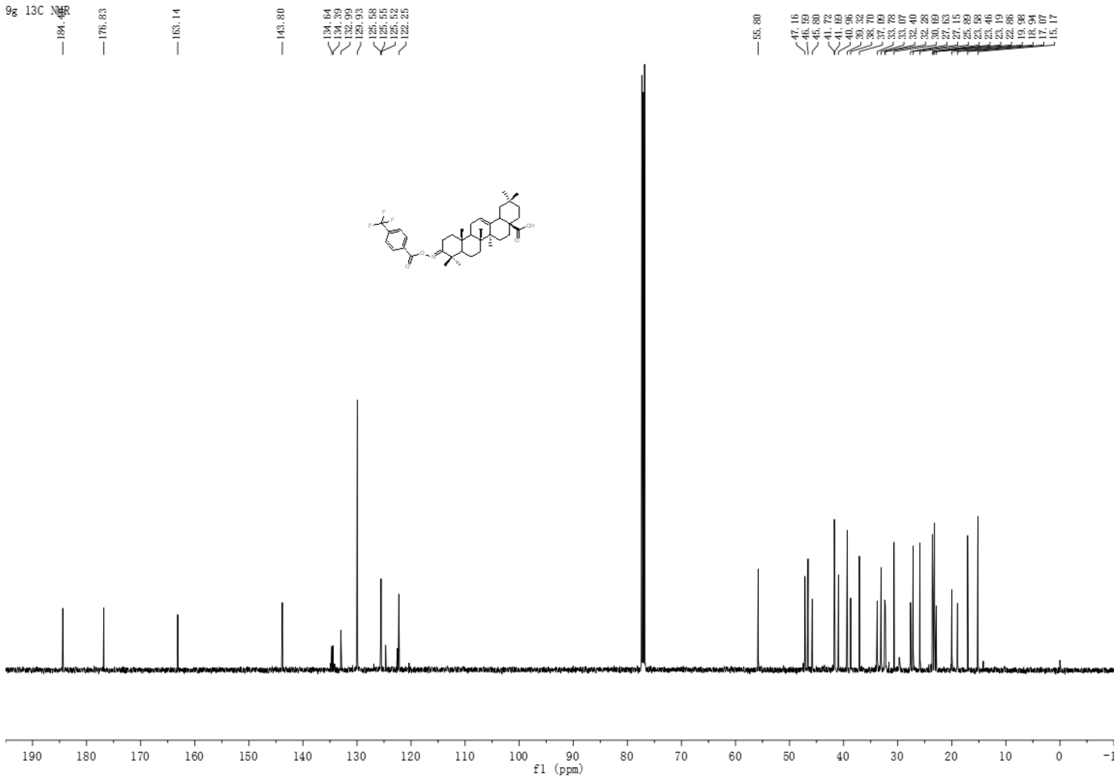

Fig.34 **3q** (^{13}C NMR)

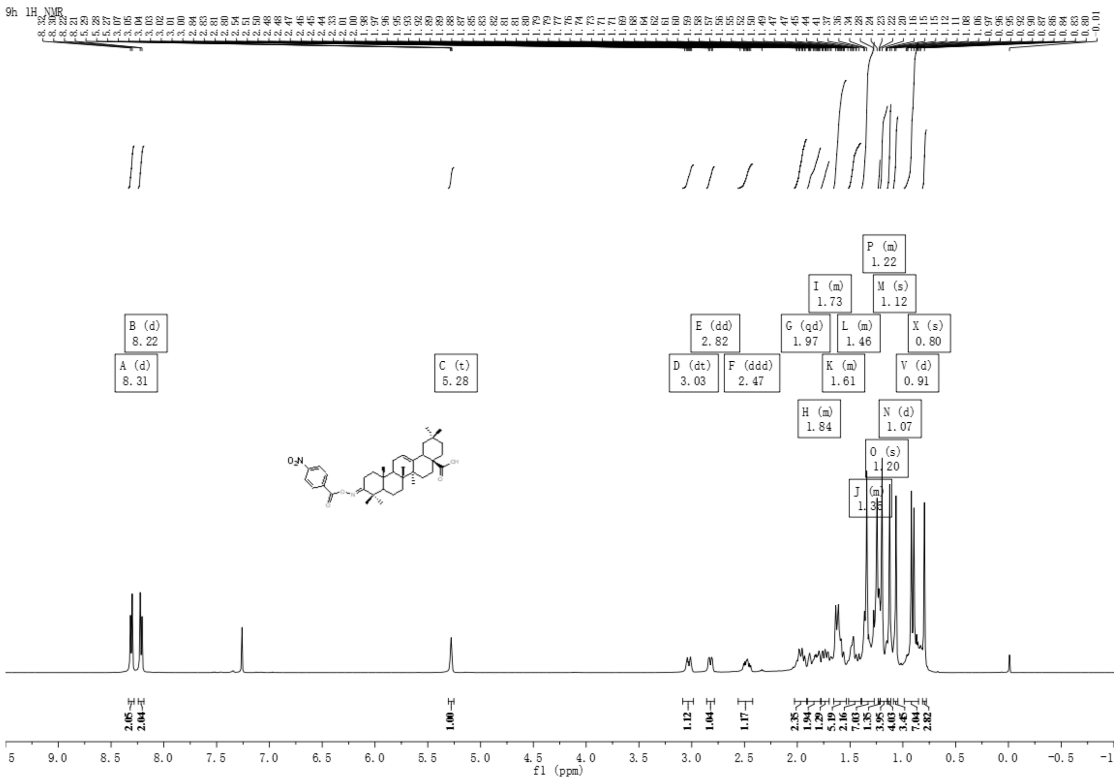

Fig.35 **3r** (^{1}H NMR)

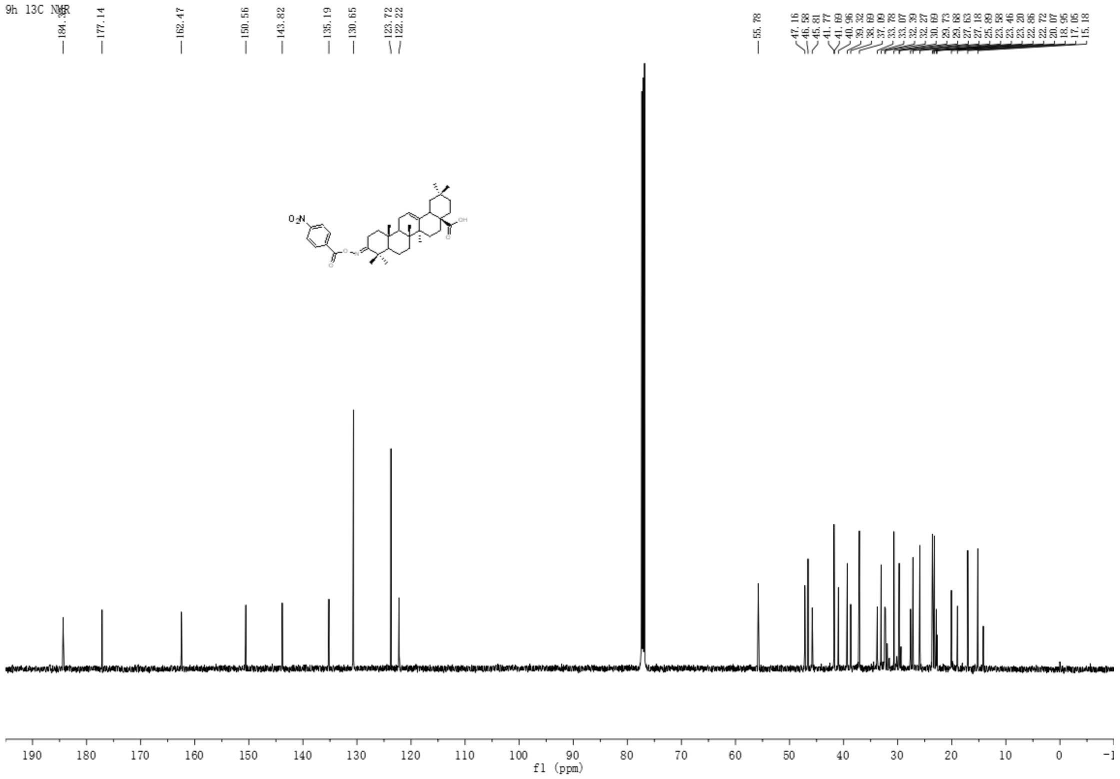

Fig.36 **3r** (^{13}C NMR)

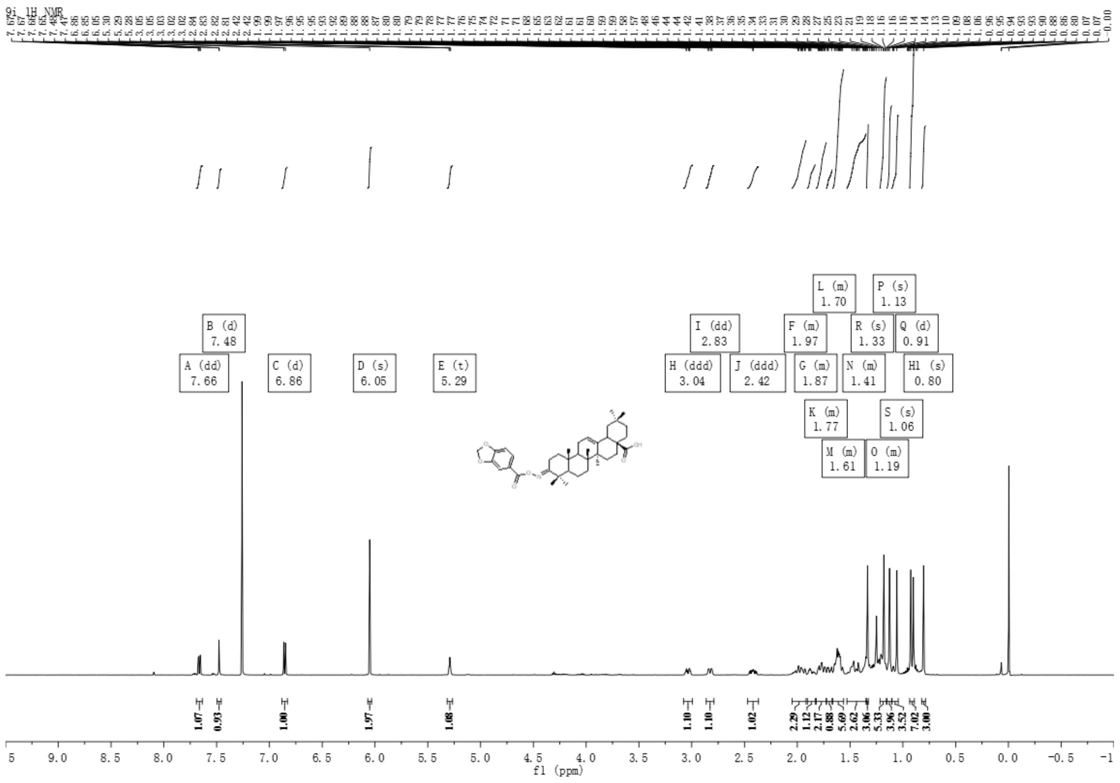

Fig.37 **3s (**^{1}H NMR)

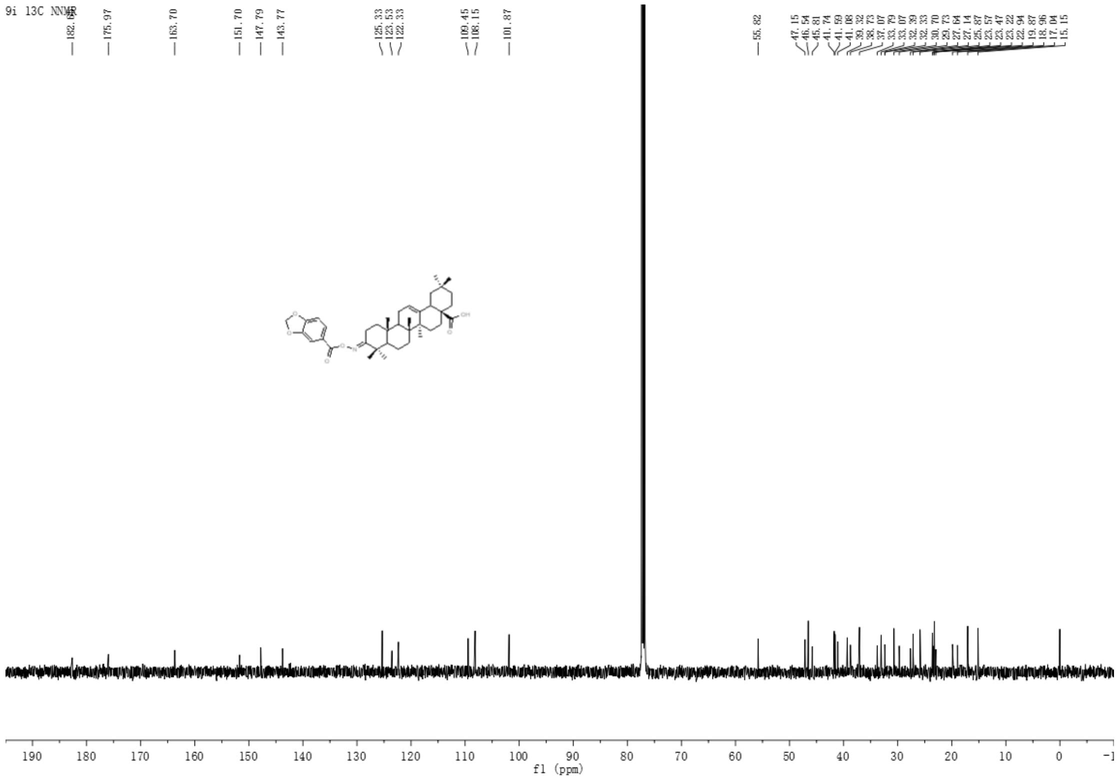

Fig.38 **3s (**^{13}C NMR)

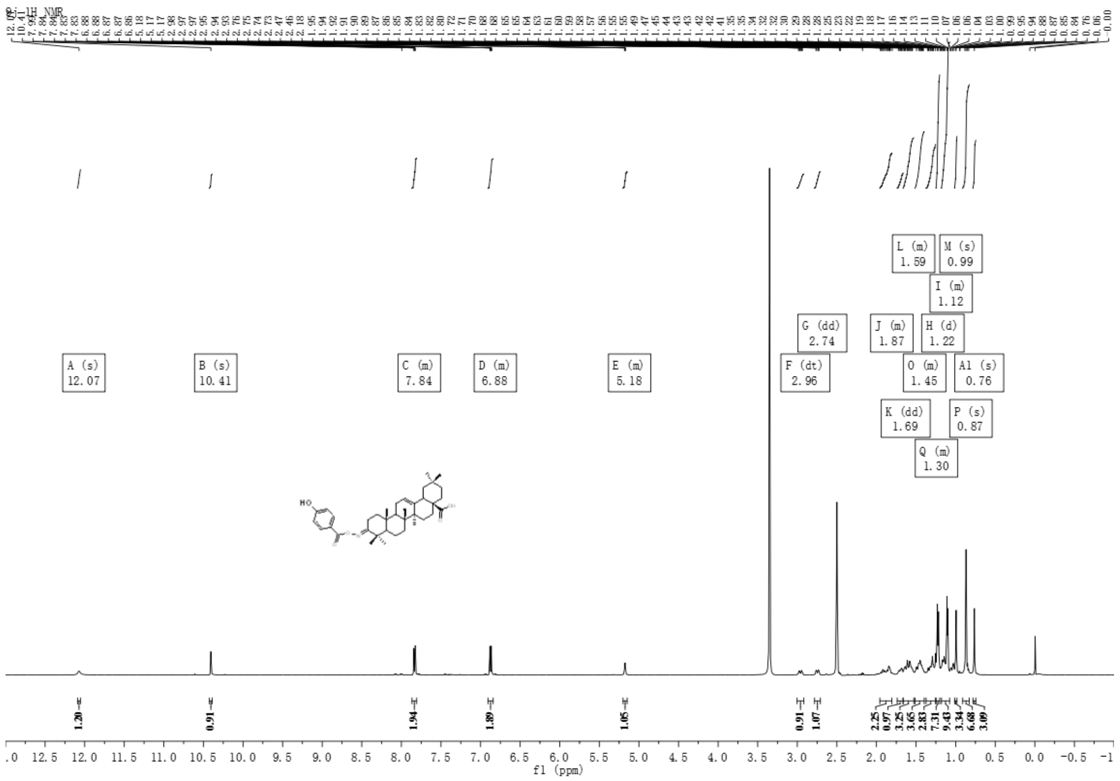

Fig.39 **3t (**^{1}H NMR)

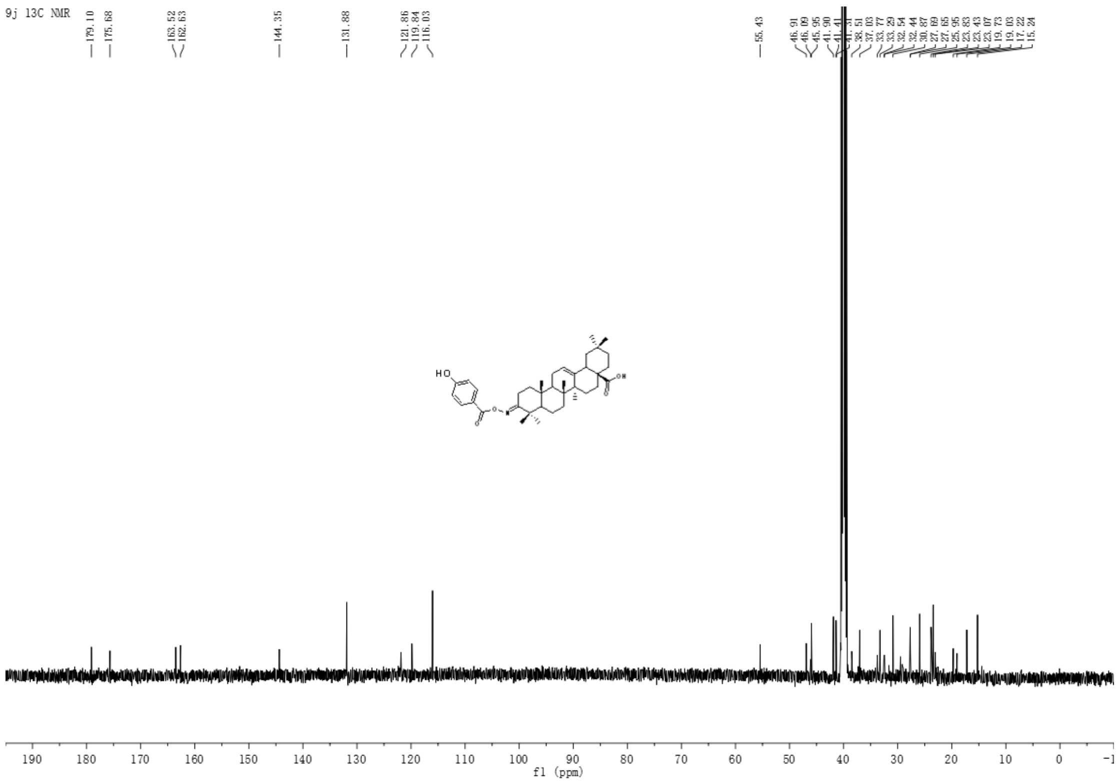

Fig.40 **3t (**^{13}C NMR)

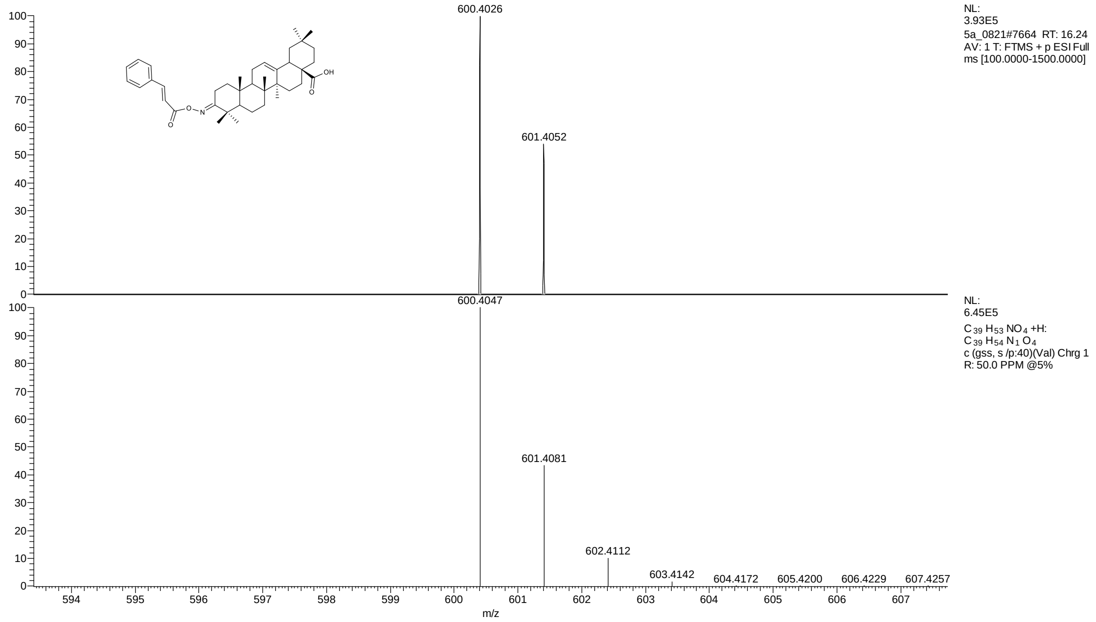

Fig.41 **3a (**HRMS)

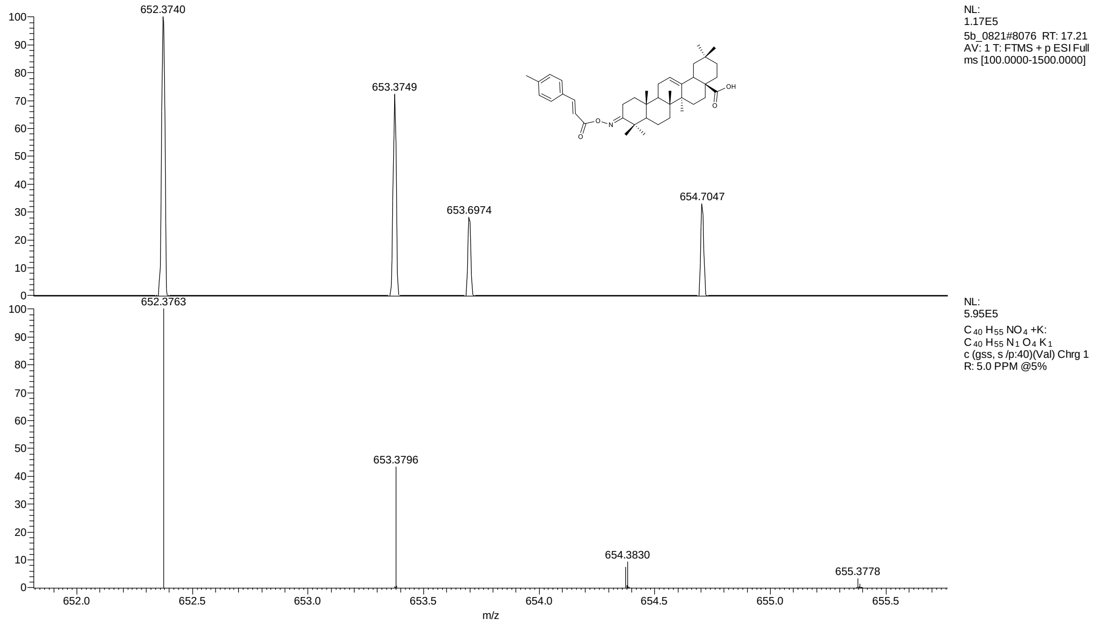

Fig.42 **3b (**HRMS)

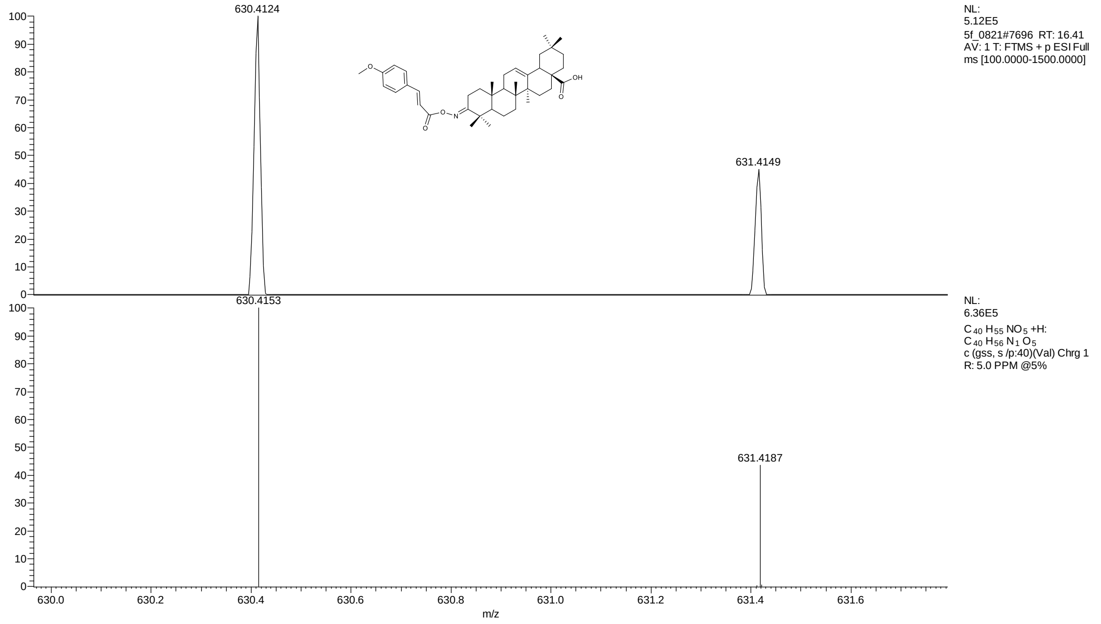

Fig.43 **3c (**HRMS)

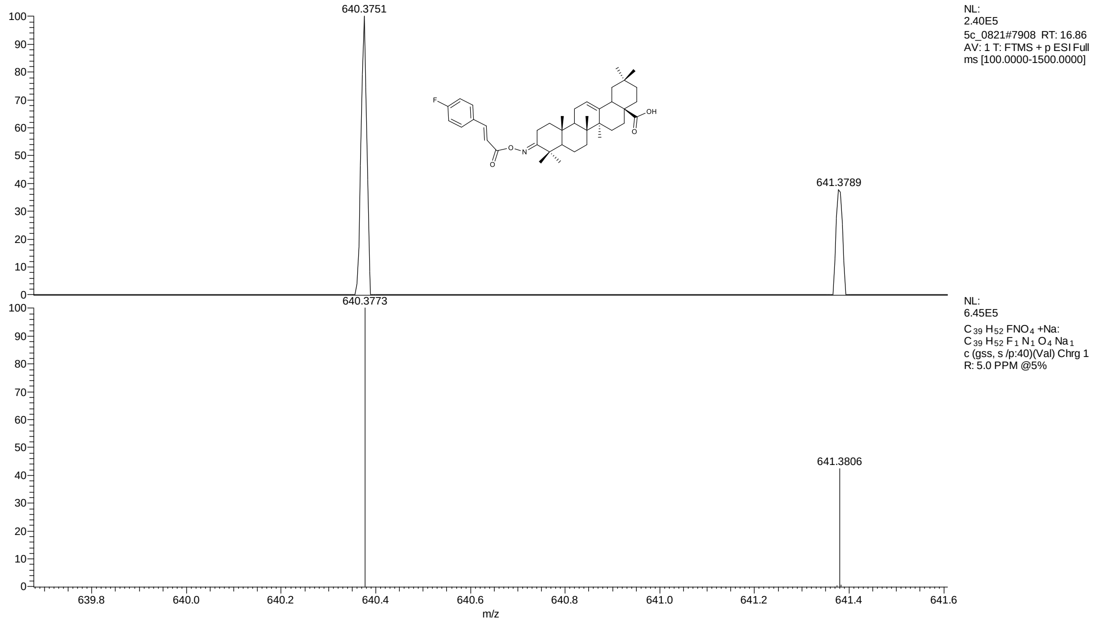

Fig.44 **3d (**HRMS)

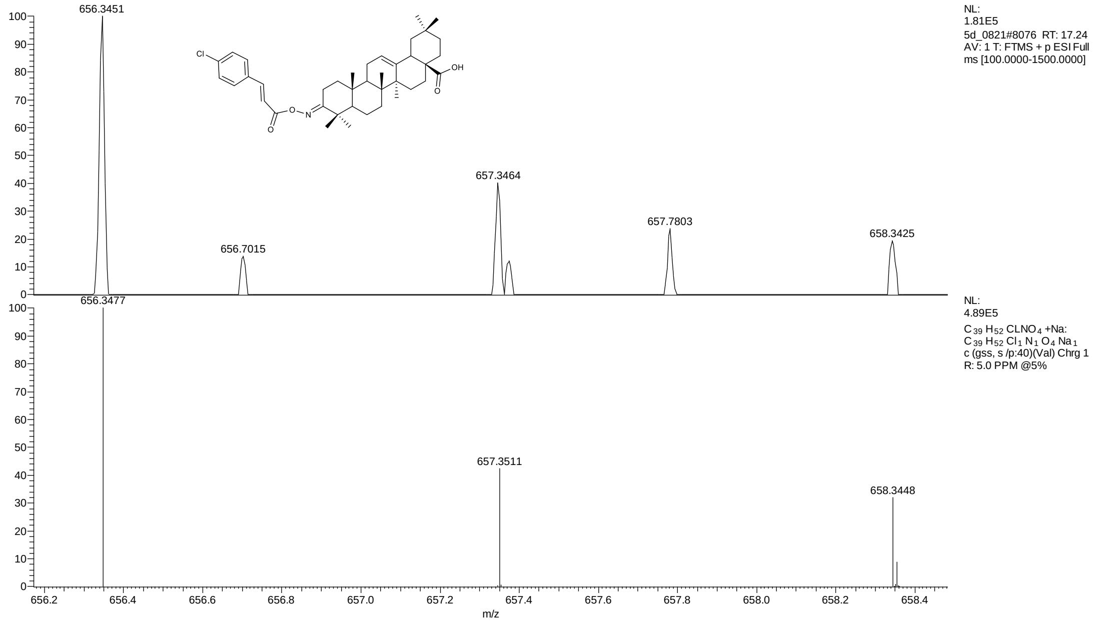

Fig.45 **3e (**HRMS)

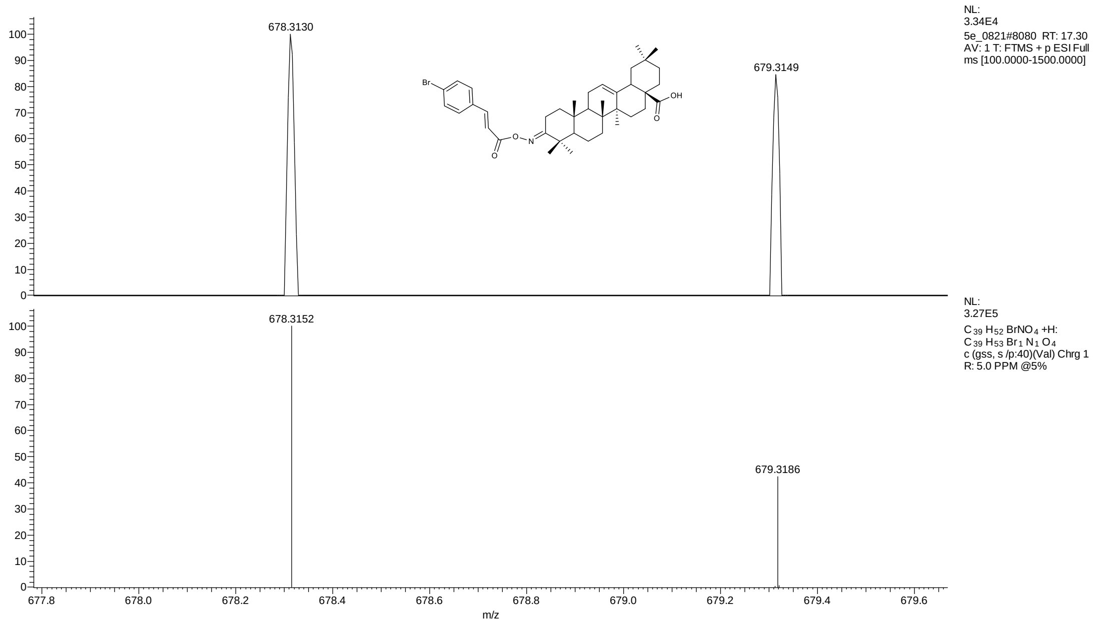

Fig.46 **3f (**HRMS)

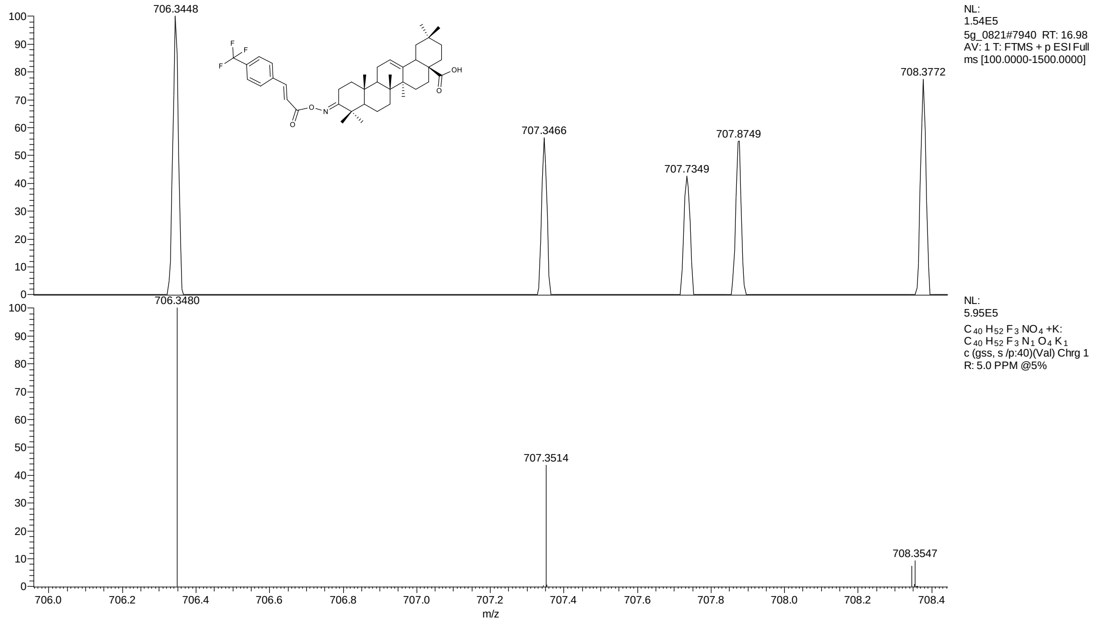

Fig.47 **3g (**HRMS)

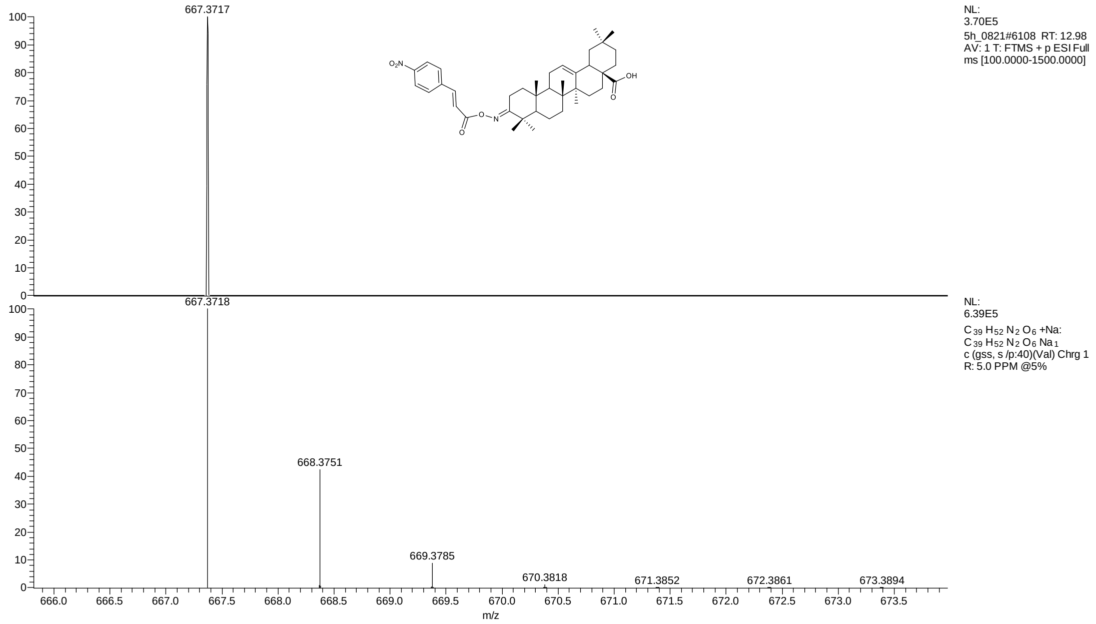

Fig.48 **3h (**HRMS)

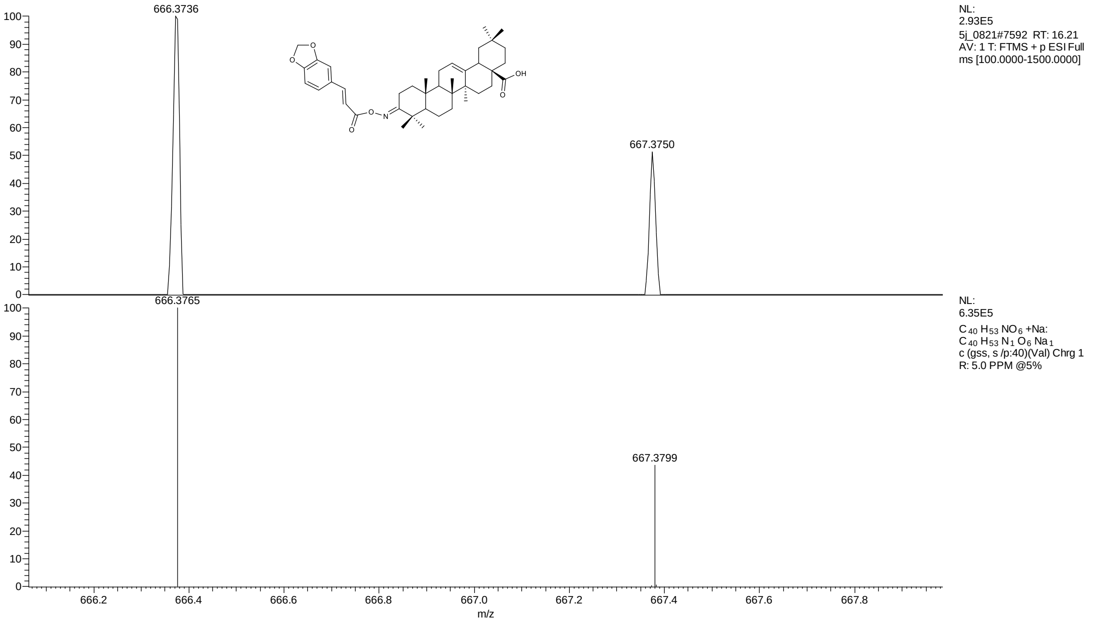

Fig.49 **3i (**HRMS)

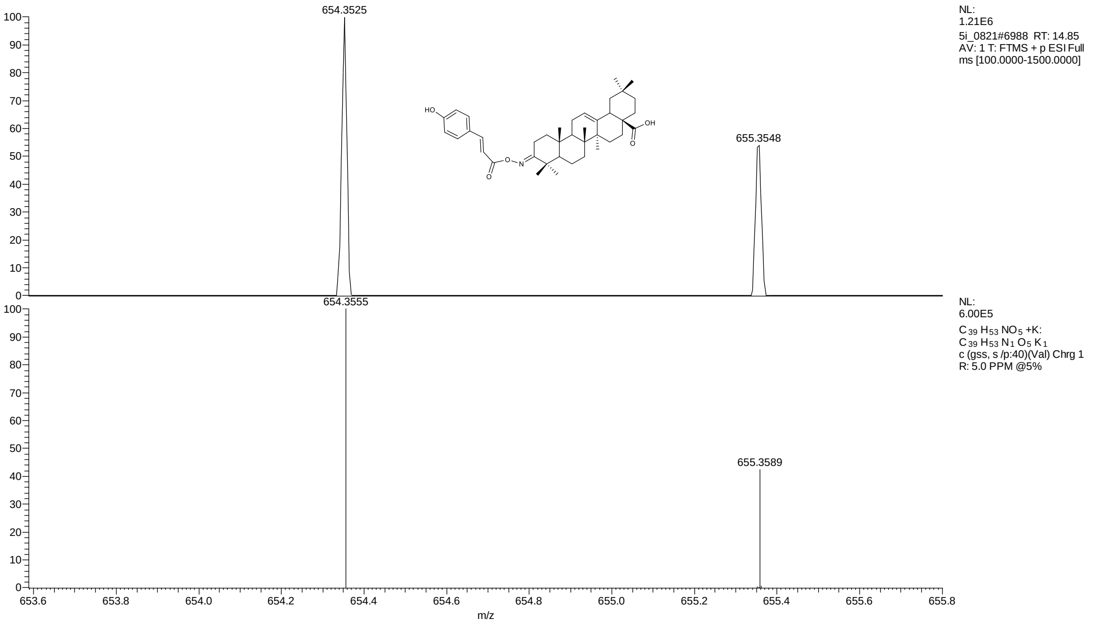

Fig.50 **3j (**HRMS)

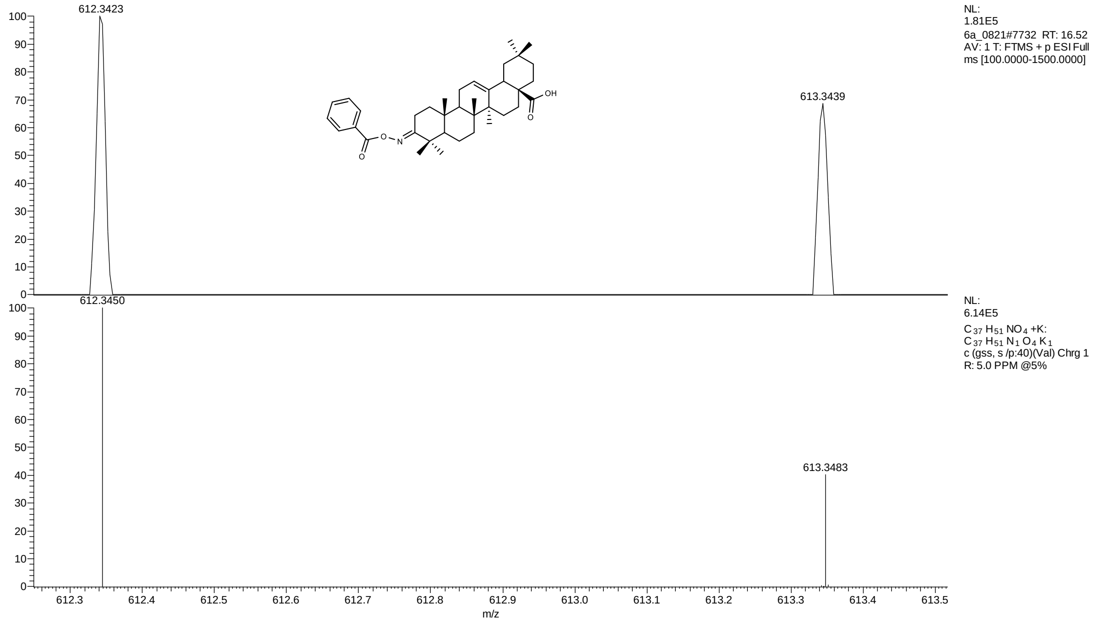

Fig.51 **3k (**HRMS)

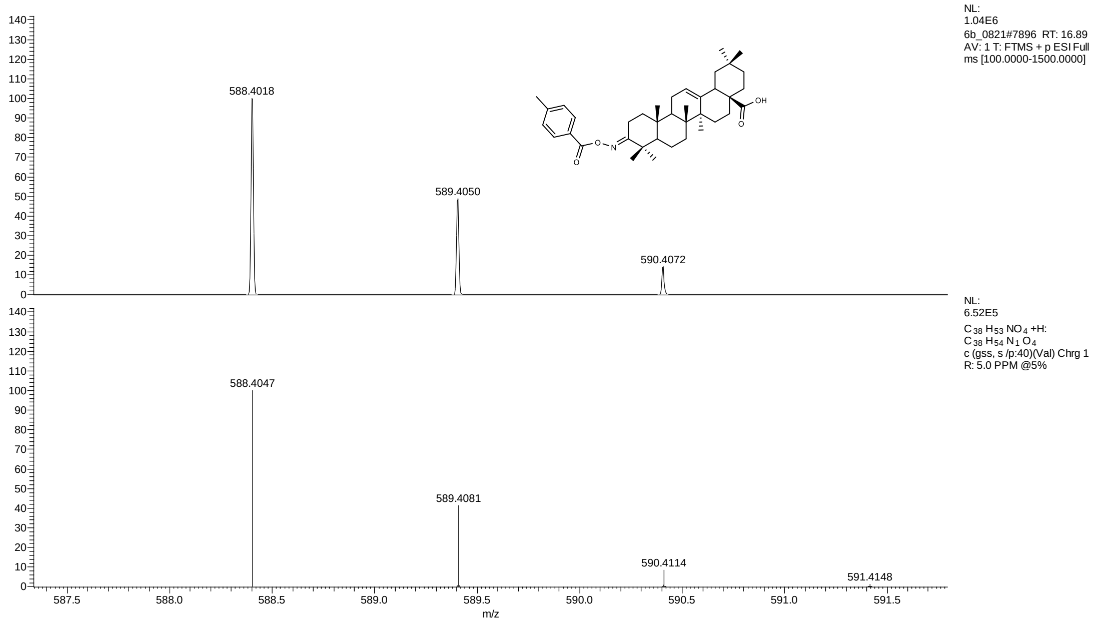

Fig.52 **3l (**HRMS)

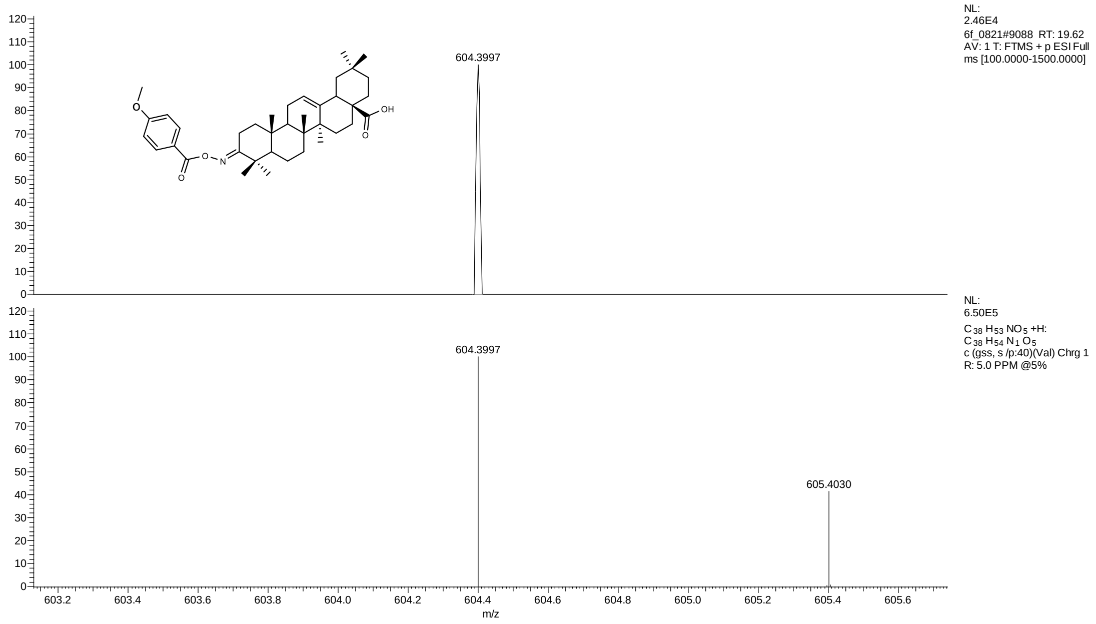

Fig.53 **3m (**HRMS)

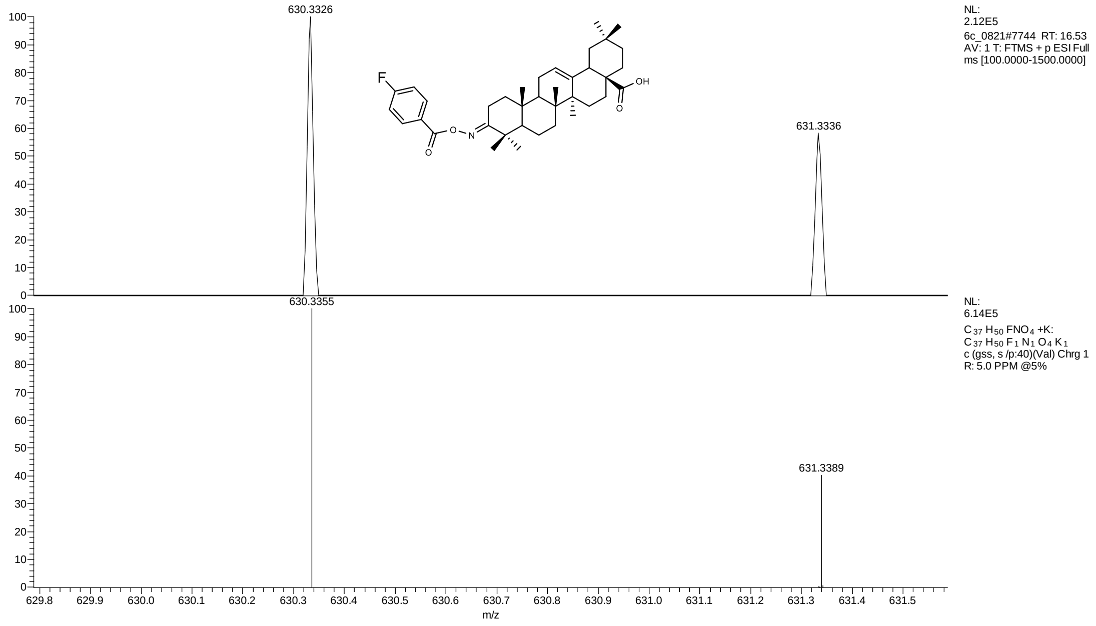

Fig.54 **3n (**HRMS)

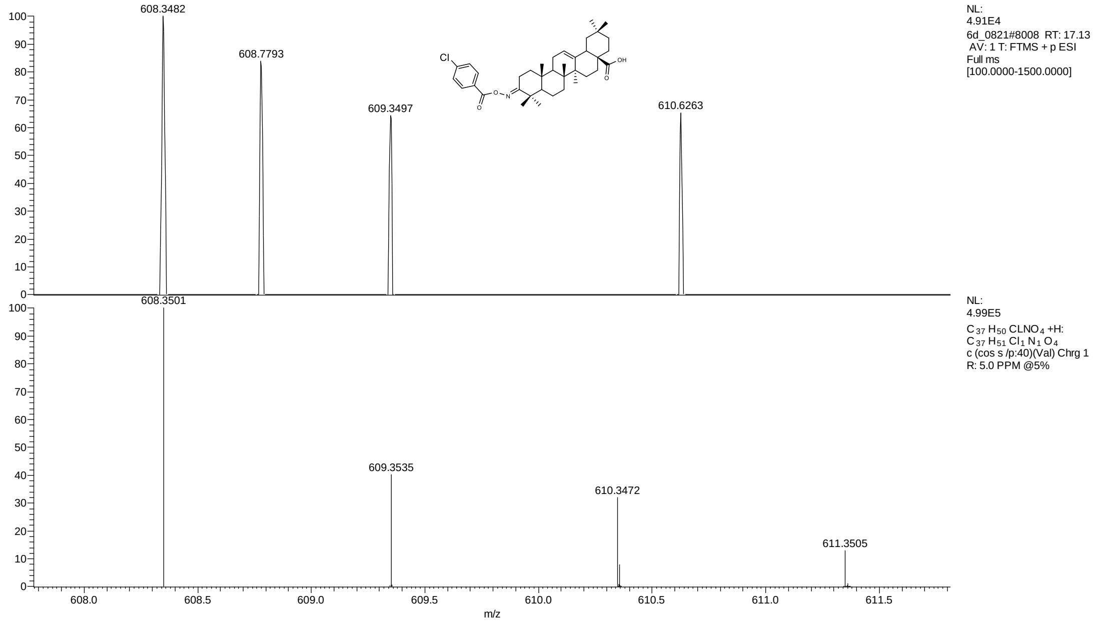

Fig.55 **3o (**HRMS)

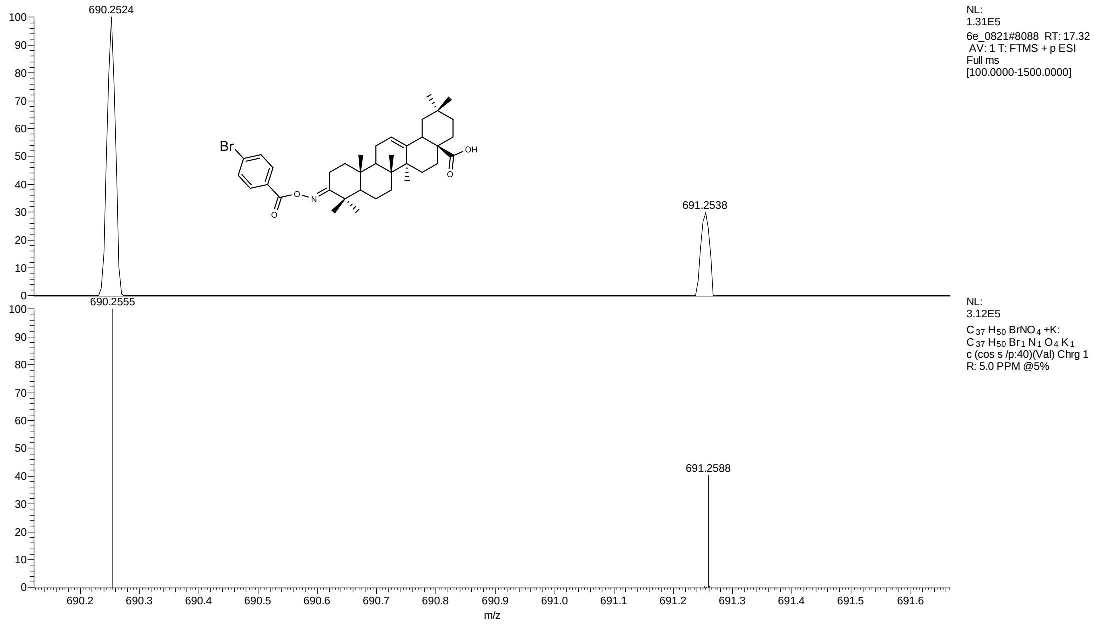

Fig.56 **3p (**HRMS)

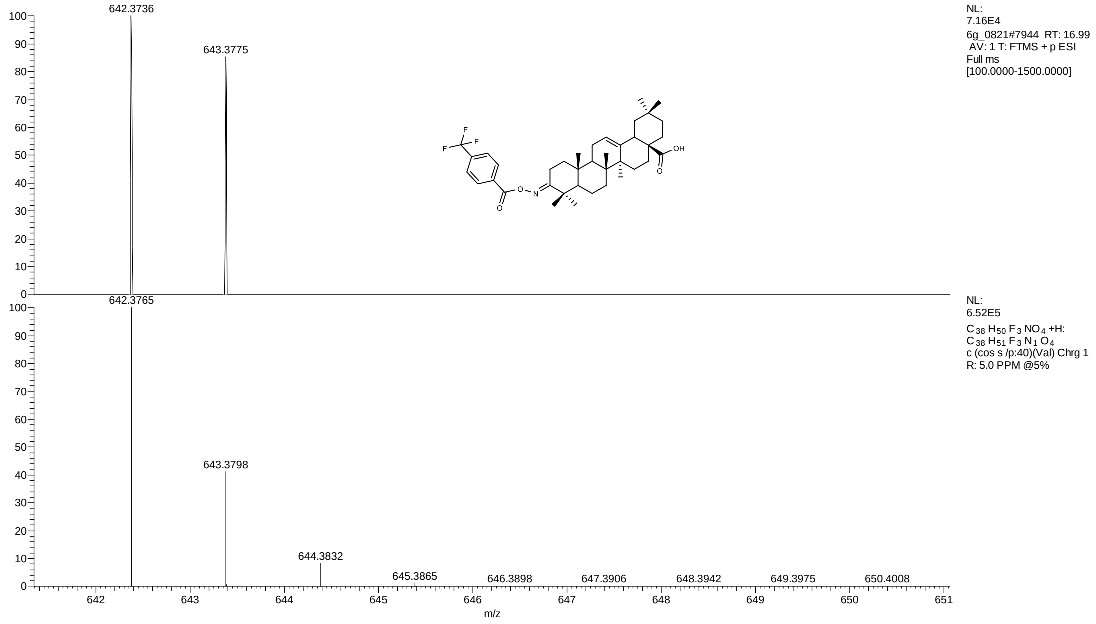

Fig.57 **3q (**HRMS)

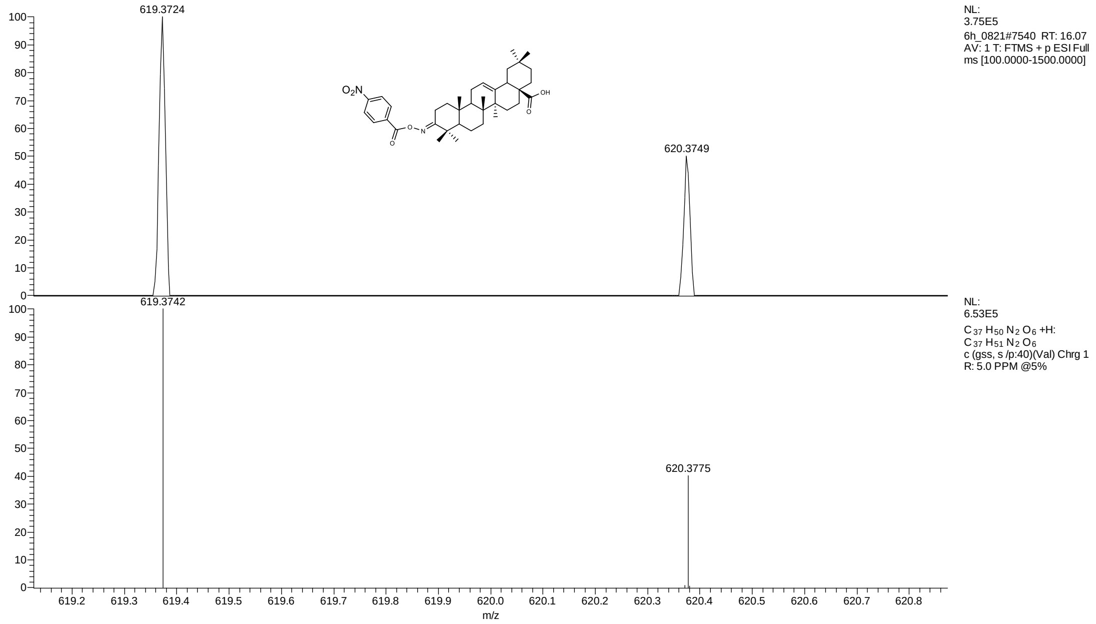

Fig.58 **3r (**HRMS)

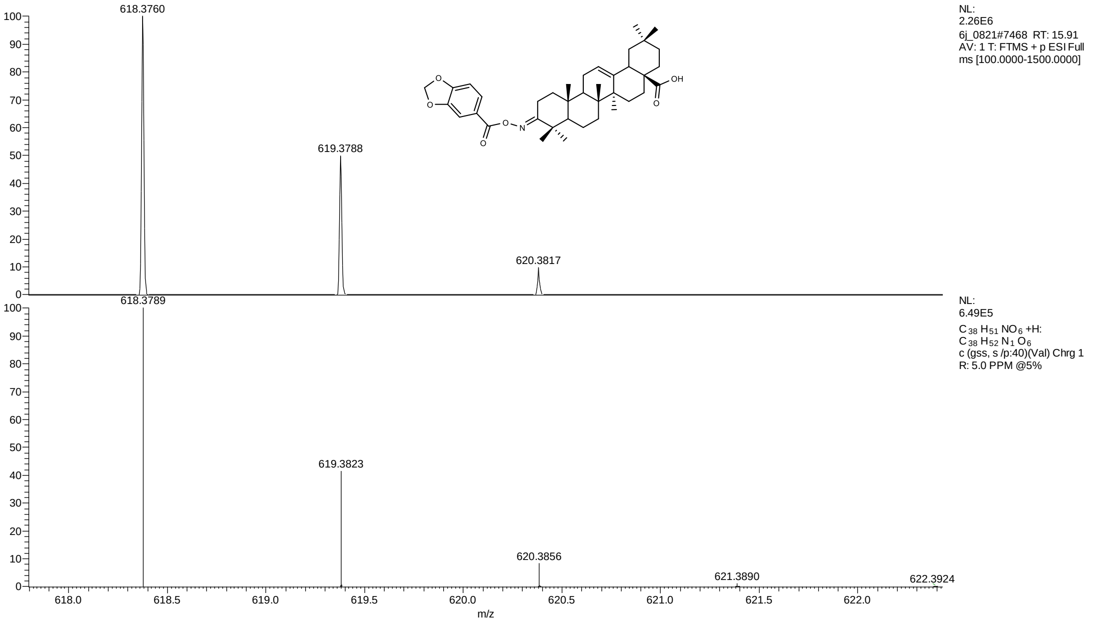

Fig.59 **3s (**HRMS)

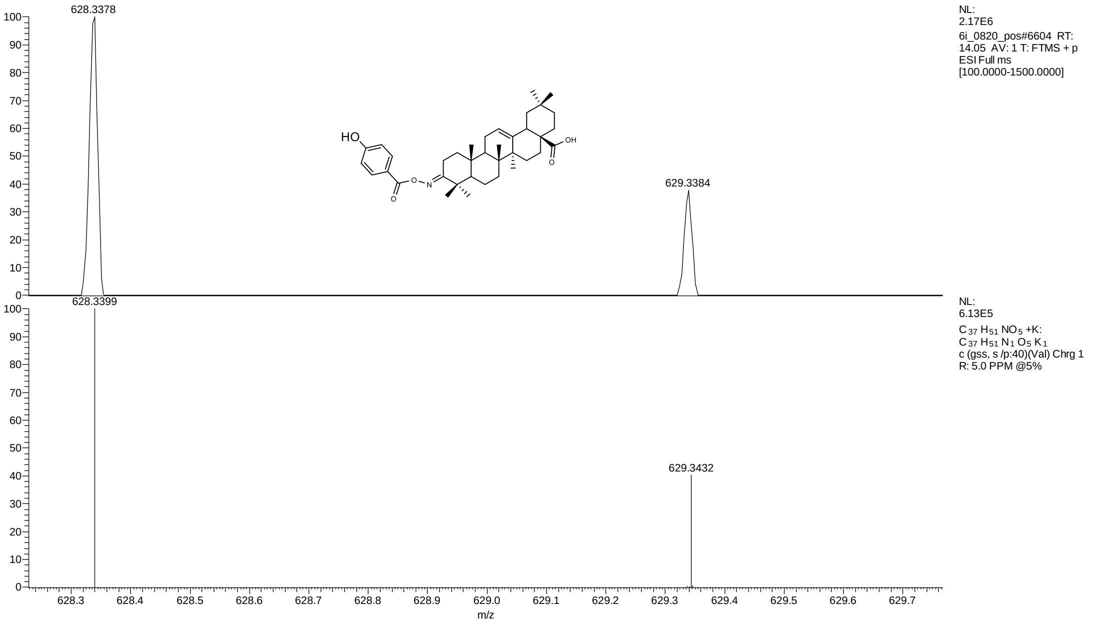

Fig.60 **3t (**HRMS)
